# Supplementary figures and images for: Acetylation of a fungal effector that translocates host PR1 facilitates virulence
Source: eLife. 2022 Nov 14;11:e82628. doi: 10.7554/eLife.82628 (PMC9681213; doi:10.7554/eLife.82628)

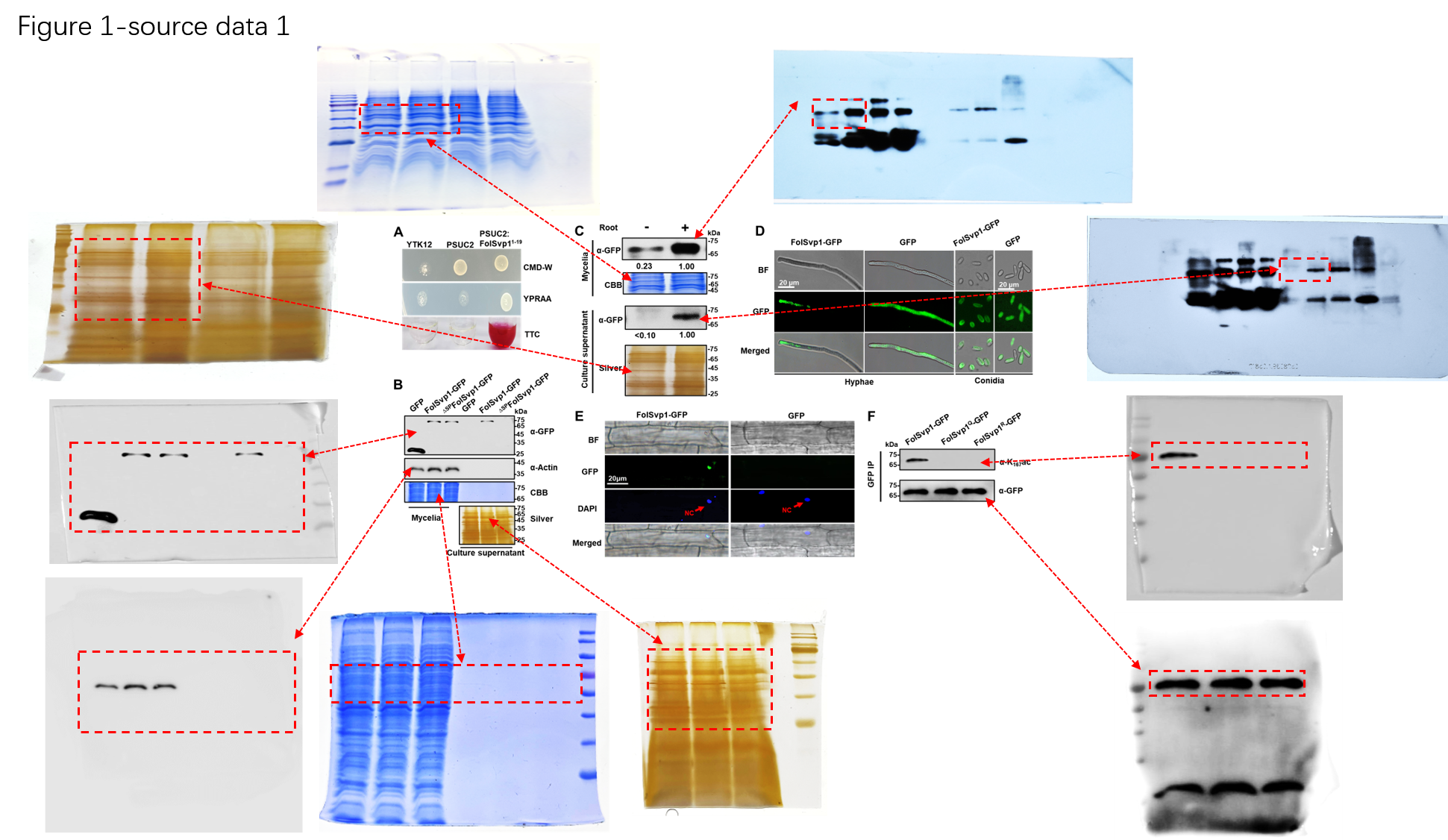

Supplement: Figure 1—source data 1. [file elife-82628-fig1-data1.zip › Figure 1-source data1/figures with the uncropped gels or blots.tif]

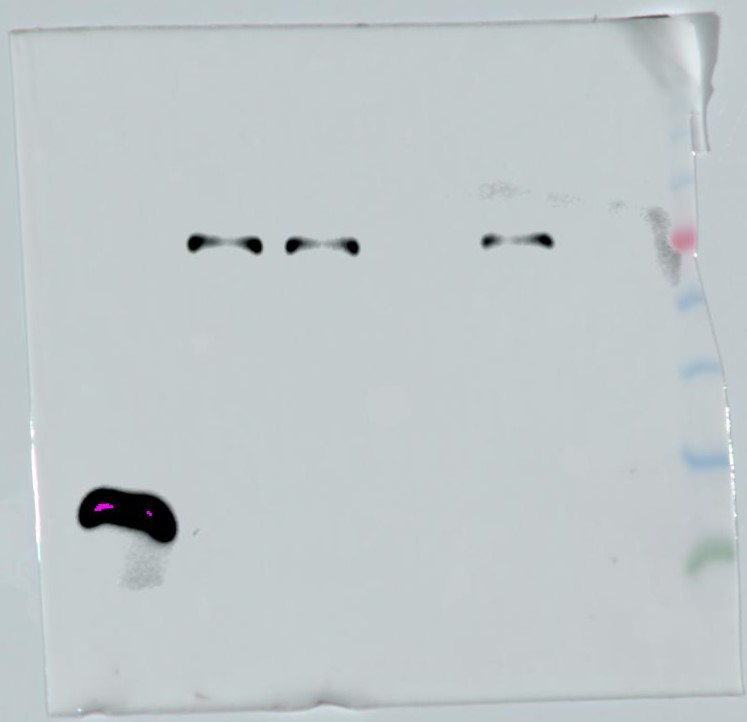

Supplement: Figure 1—source data 1. [file elife-82628-fig1-data1.zip › Figure 1-source data1/raw unedited gels or blots/Figure 1-source data 1-1.tif]

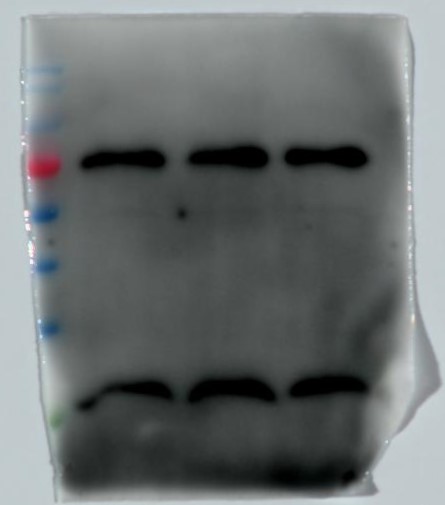

Supplement: Figure 1—source data 1. [file elife-82628-fig1-data1.zip › Figure 1-source data1/raw unedited gels or blots/Figure 1-source data 1-10.tif]

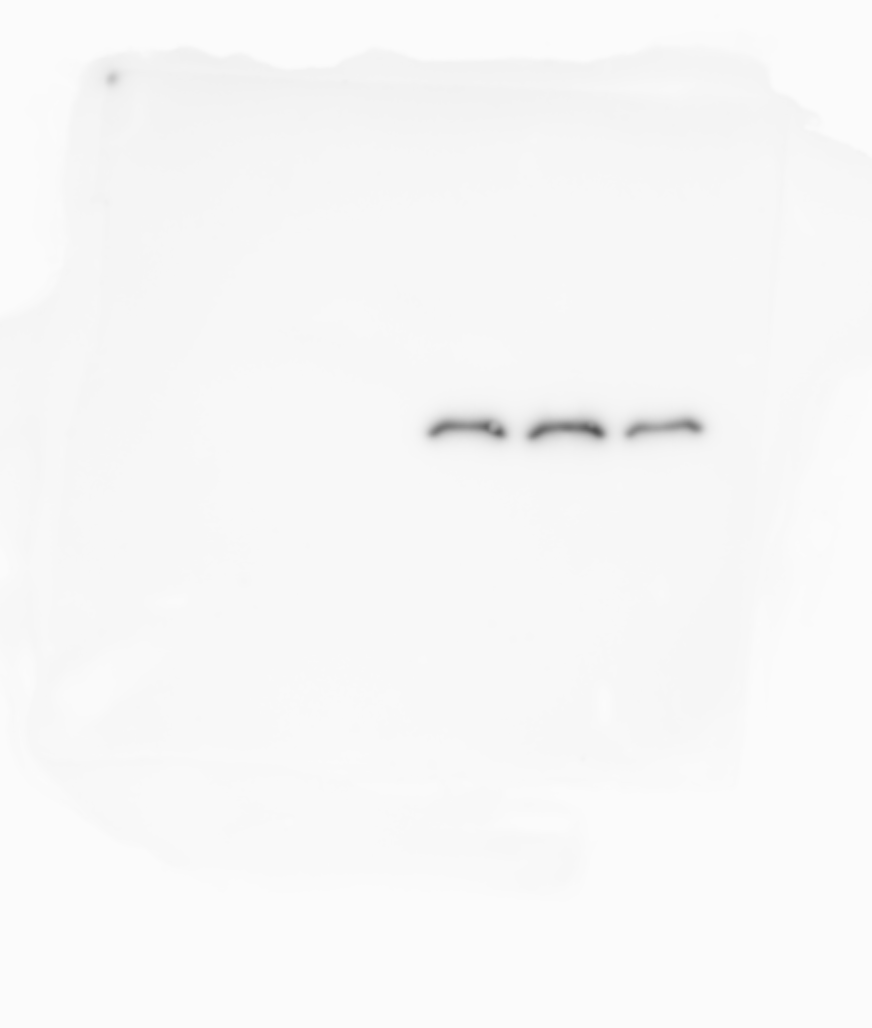

Supplement: Figure 1—source data 1. [file elife-82628-fig1-data1.zip › Figure 1-source data1/raw unedited gels or blots/Figure 1-source data 1-2.tif]

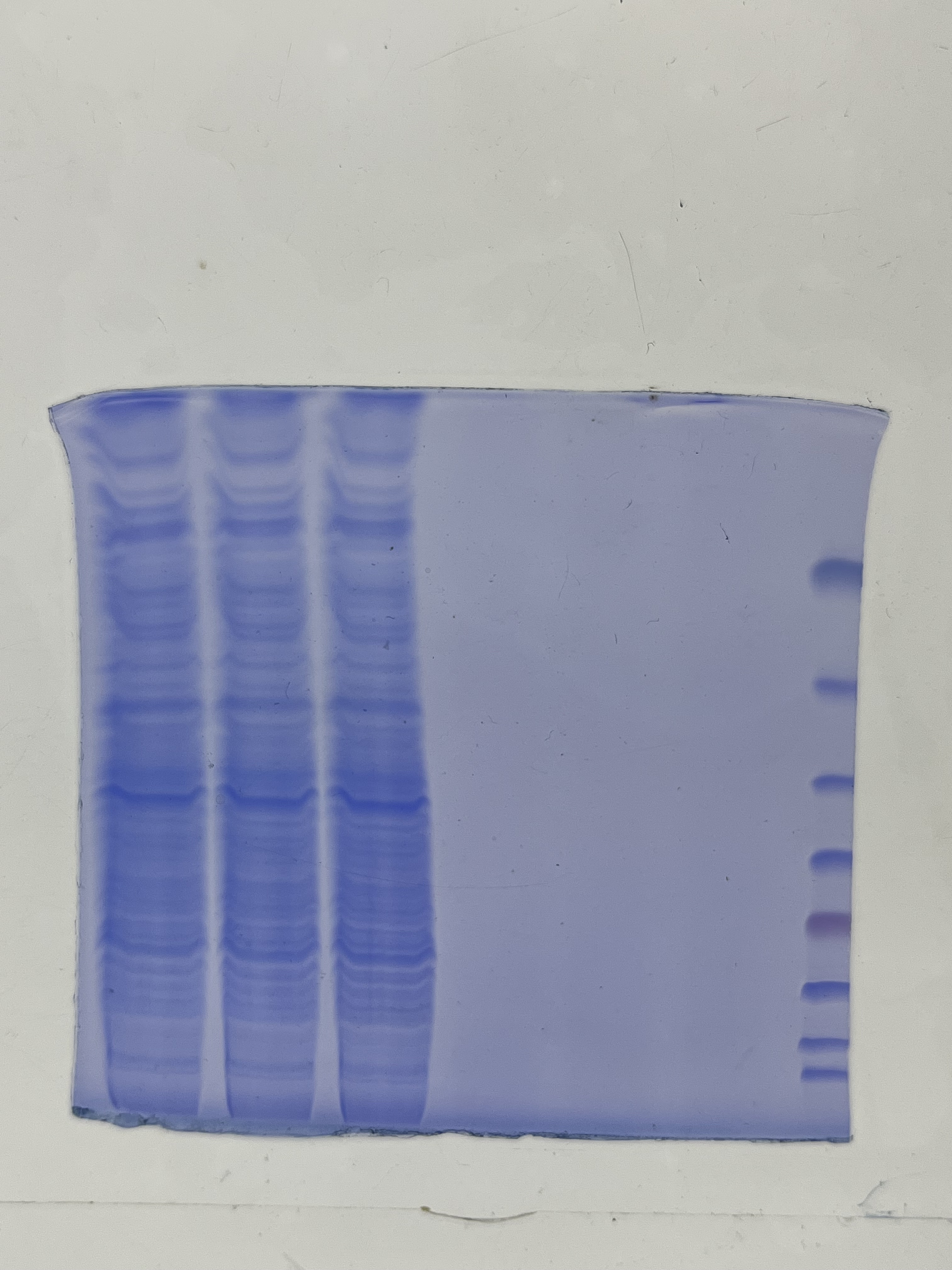

Supplement: Figure 1—source data 1. [file elife-82628-fig1-data1.zip › Figure 1-source data1/raw unedited gels or blots/Figure 1-source data 1-3.tif]

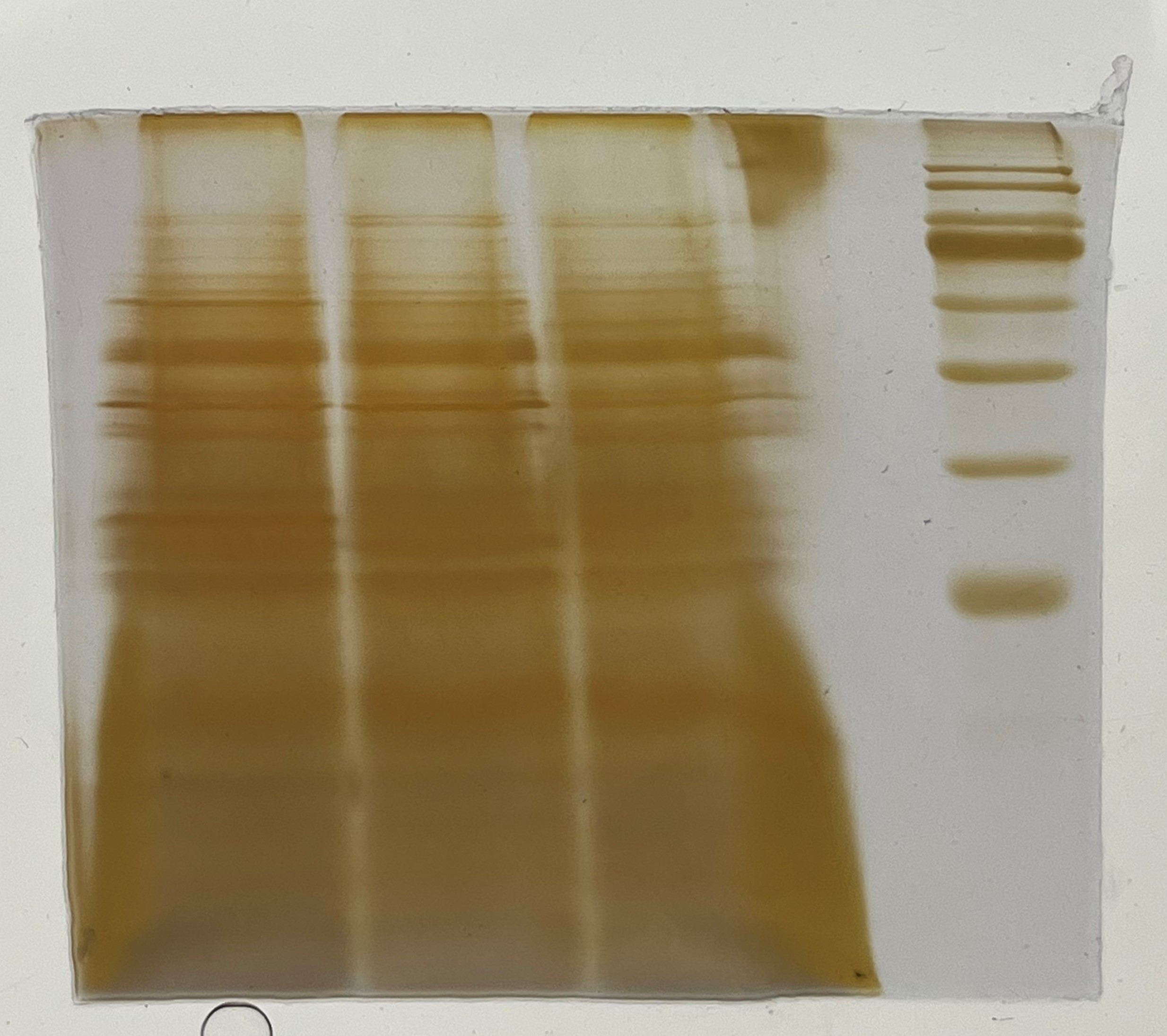

Supplement: Figure 1—source data 1. [file elife-82628-fig1-data1.zip › Figure 1-source data1/raw unedited gels or blots/Figure 1-source data 1-4.tif]

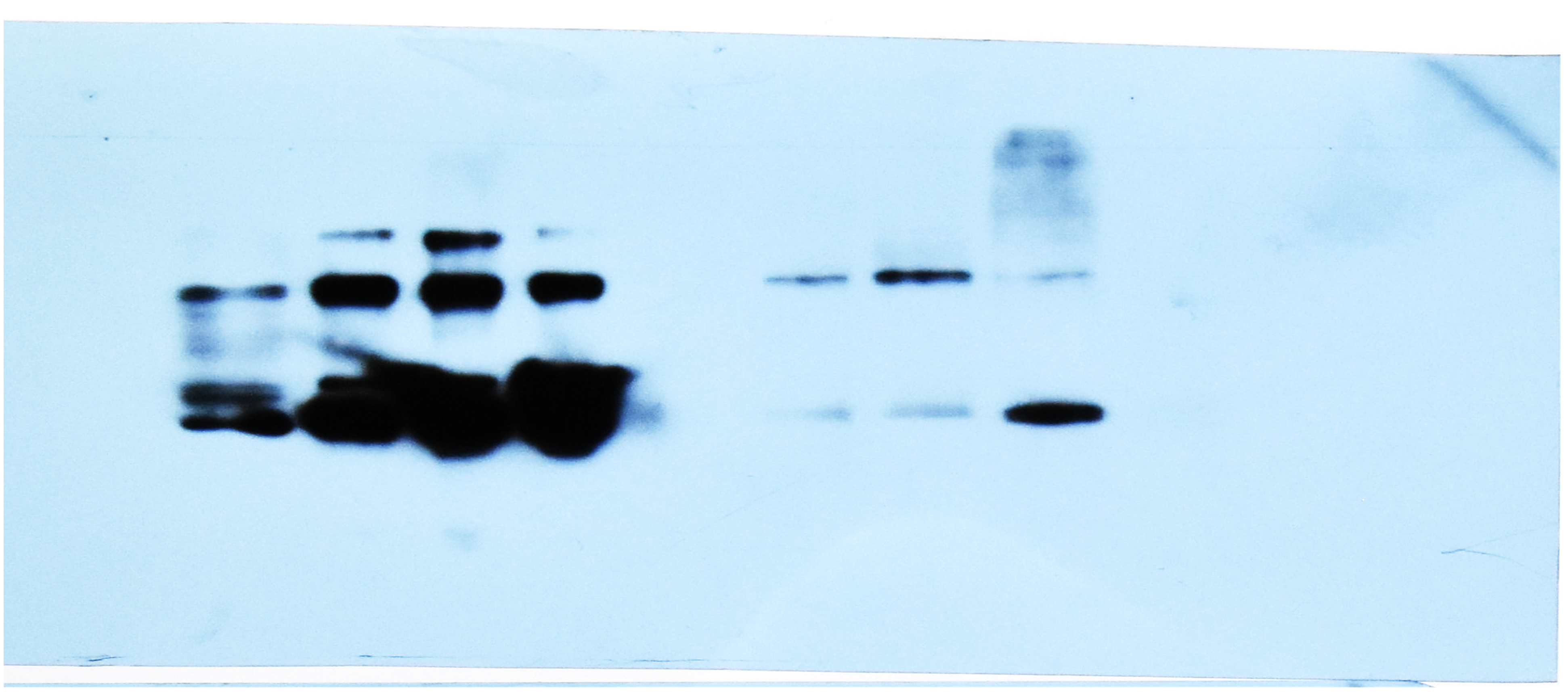

Supplement: Figure 1—source data 1. [file elife-82628-fig1-data1.zip › Figure 1-source data1/raw unedited gels or blots/Figure 1-source data 1-5.tif]

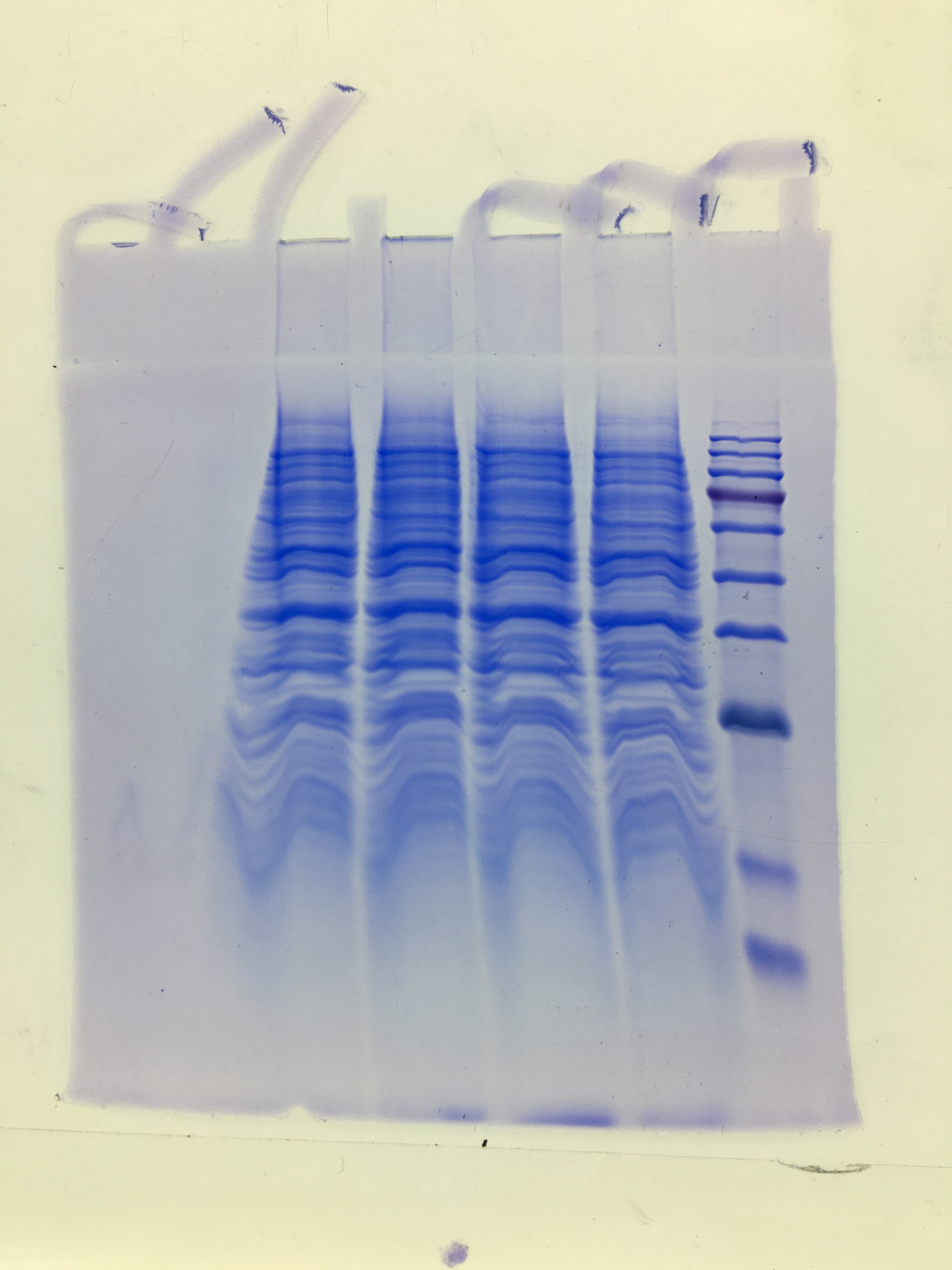

Supplement: Figure 1—source data 1. [file elife-82628-fig1-data1.zip › Figure 1-source data1/raw unedited gels or blots/Figure 1-source data 1-6.tif]

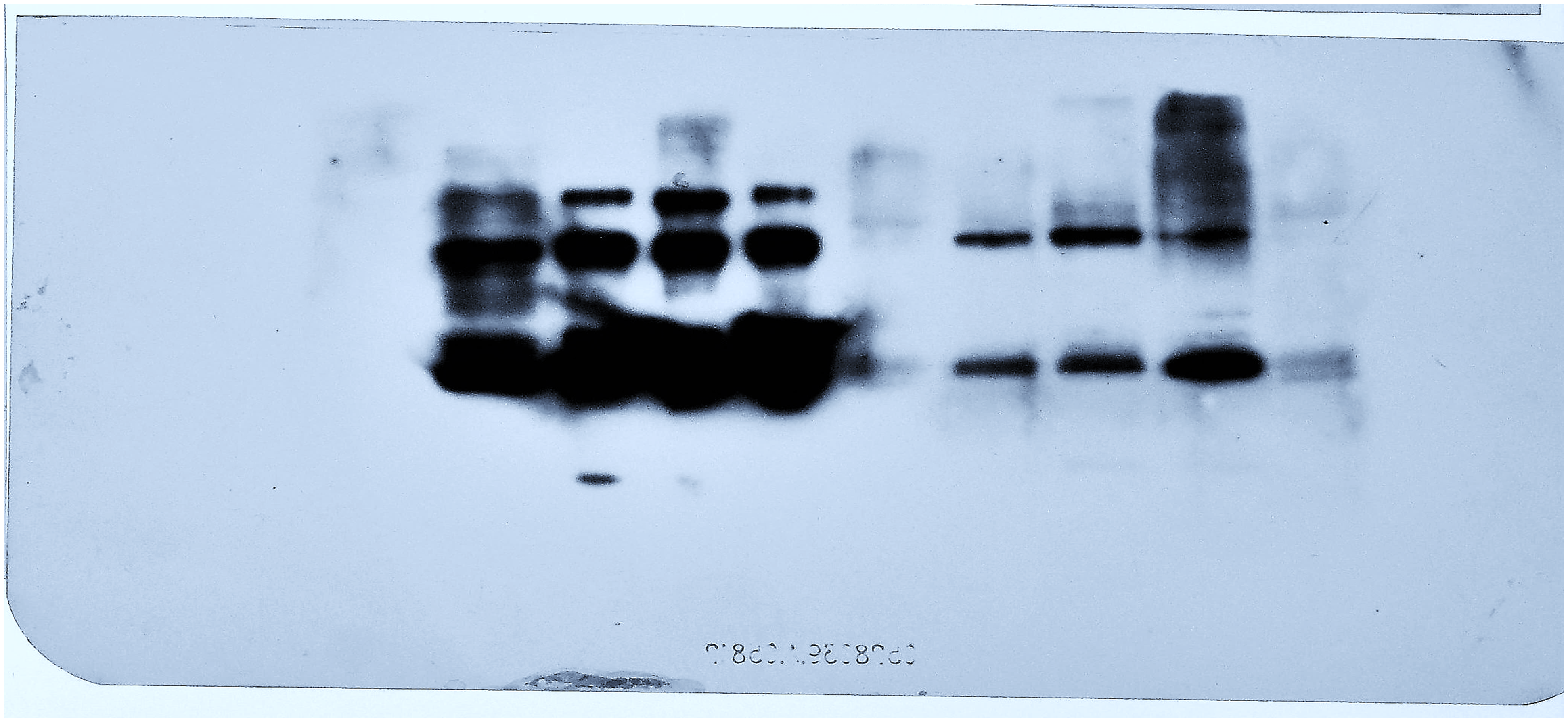

Supplement: Figure 1—source data 1. [file elife-82628-fig1-data1.zip › Figure 1-source data1/raw unedited gels or blots/Figure 1-source data 1-7.tif]

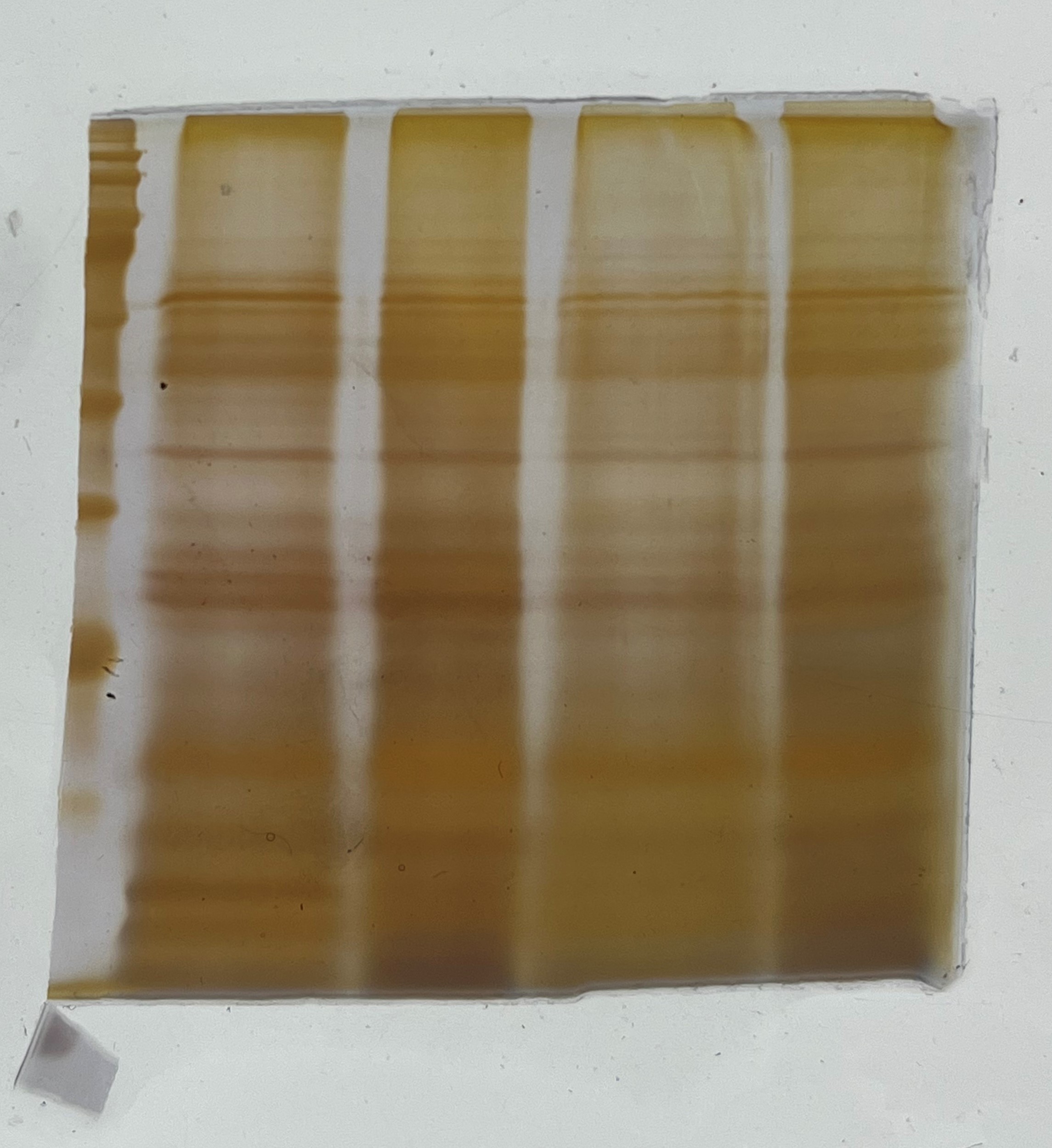

Supplement: Figure 1—source data 1. [file elife-82628-fig1-data1.zip › Figure 1-source data1/raw unedited gels or blots/Figure 1-source data 1-8.tif]

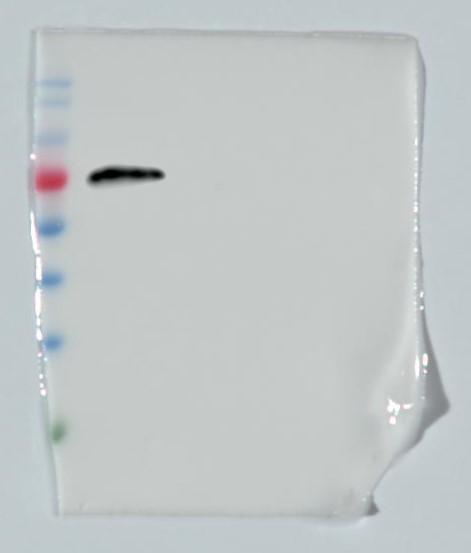

Supplement: Figure 1—source data 1. [file elife-82628-fig1-data1.zip › Figure 1-source data1/raw unedited gels or blots/Figure 1-source data 1-9.tif]

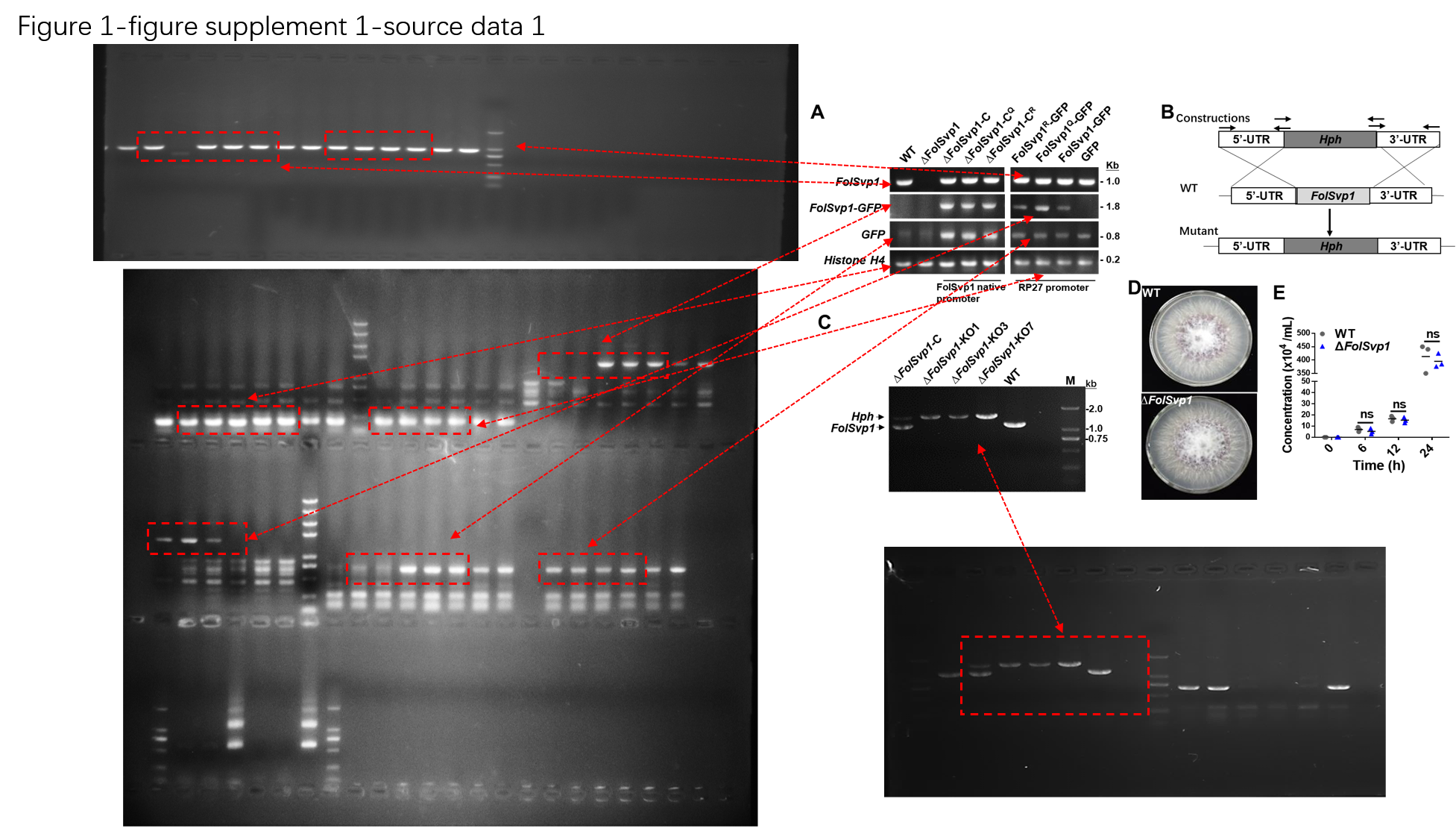

Supplement: Figure 1—figure supplement 1—source data 1. [file elife-82628-fig1-figsupp1-data1.zip › Figure 1-figure supplement 1-source data 1/figures with uncropped gels or blots.tif]

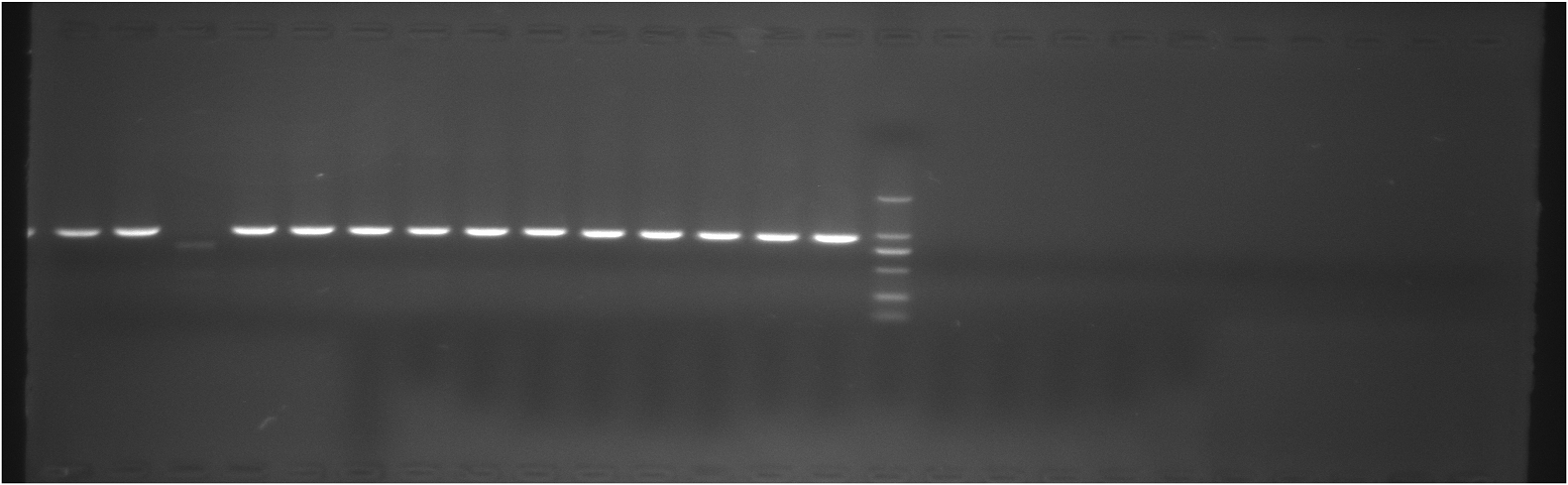

Supplement: Figure 1—figure supplement 1—source data 1. [file elife-82628-fig1-figsupp1-data1.zip › Figure 1-figure supplement 1-source data 1/raw unedited gels or blots/Figure 1-figure supplement 1-source data 1-1.tif]

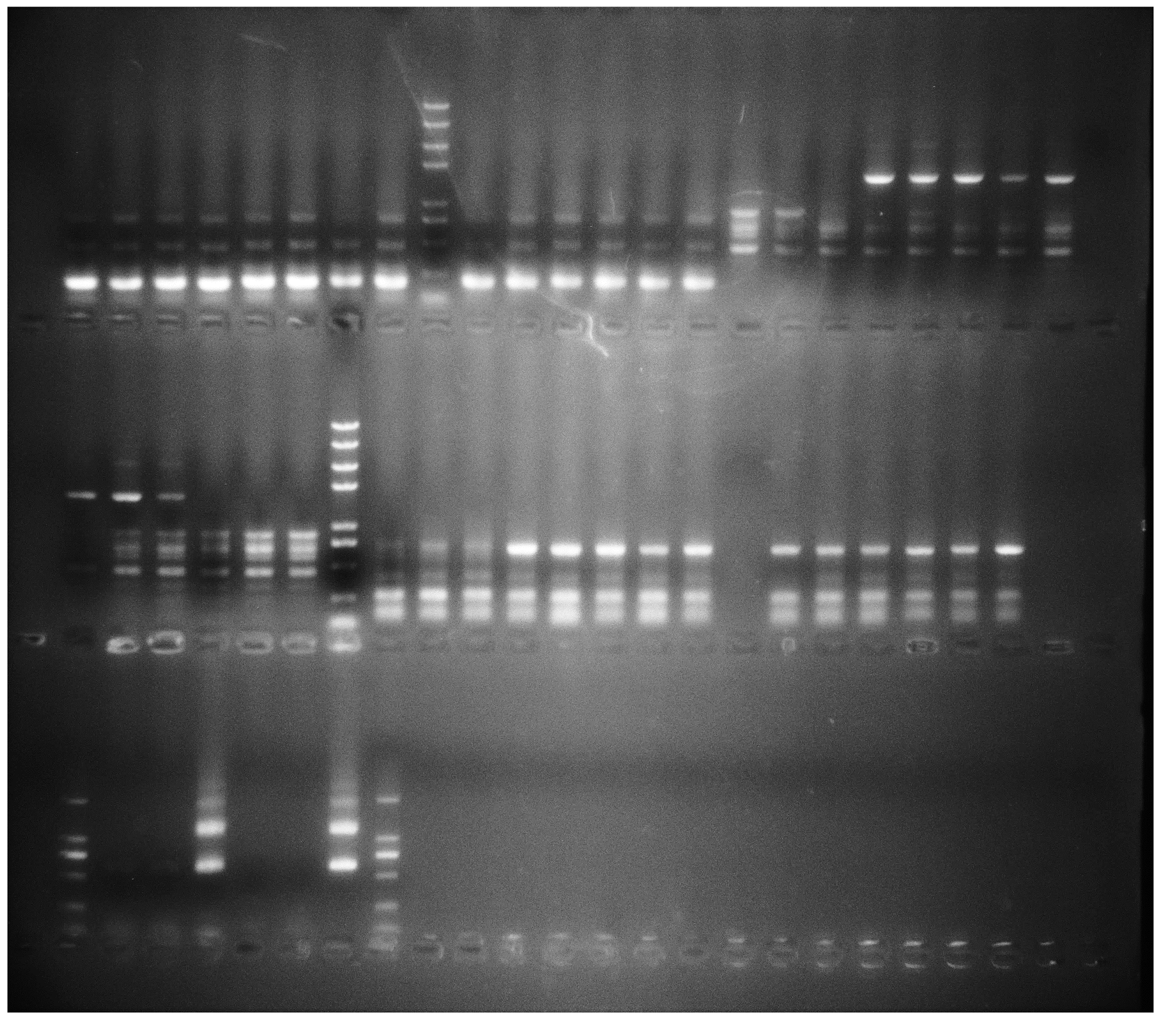

Supplement: Figure 1—figure supplement 1—source data 1. [file elife-82628-fig1-figsupp1-data1.zip › Figure 1-figure supplement 1-source data 1/raw unedited gels or blots/Figure 1-figure supplement 1-source data 1-2.tif]

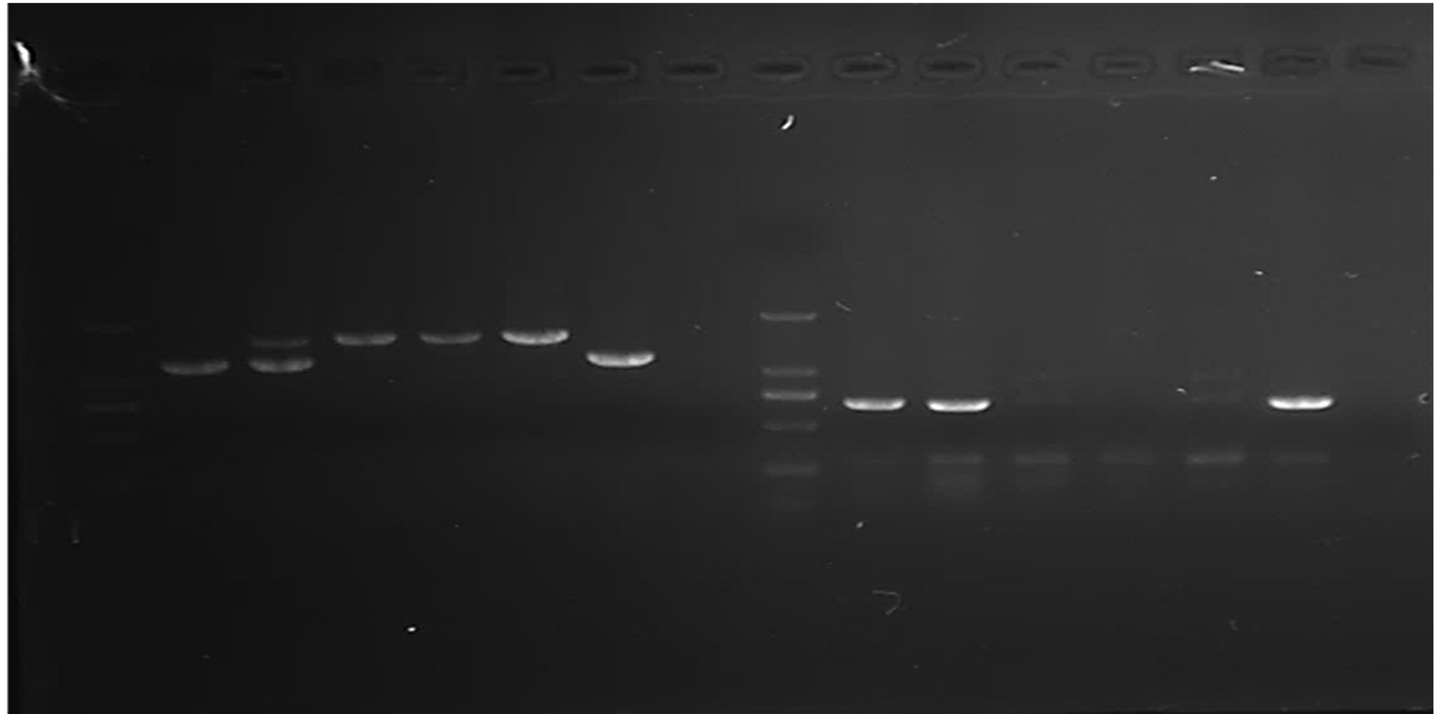

Supplement: Figure 1—figure supplement 1—source data 1. [file elife-82628-fig1-figsupp1-data1.zip › Figure 1-figure supplement 1-source data 1/raw unedited gels or blots/Figure 1-figure supplement 1-source data 1-3.tif]

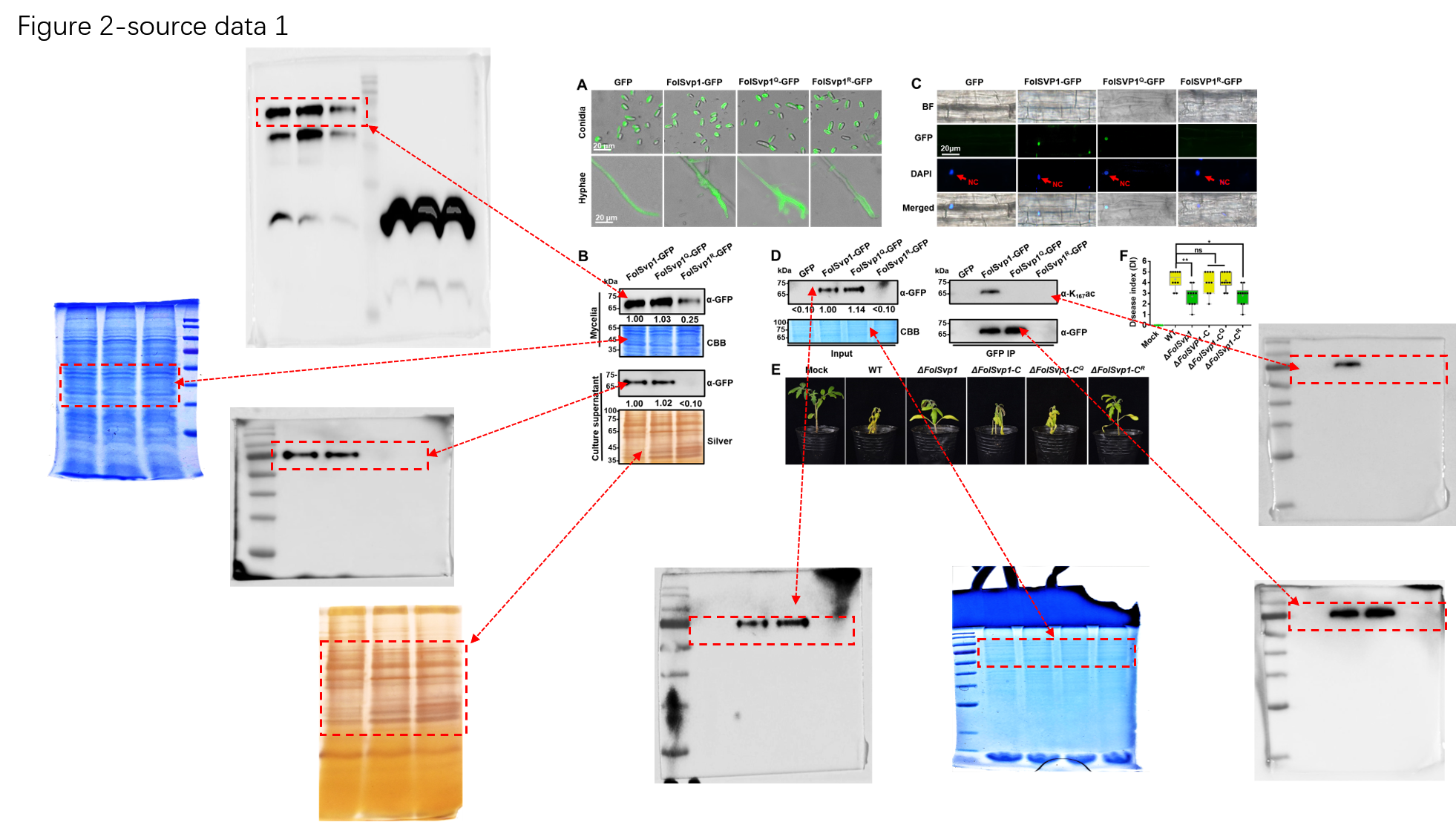

Supplement: Figure 2—source data 1. [file elife-82628-fig2-data1.zip › Figure 2-source data 1/figures with uncropped gels or blots.tif]

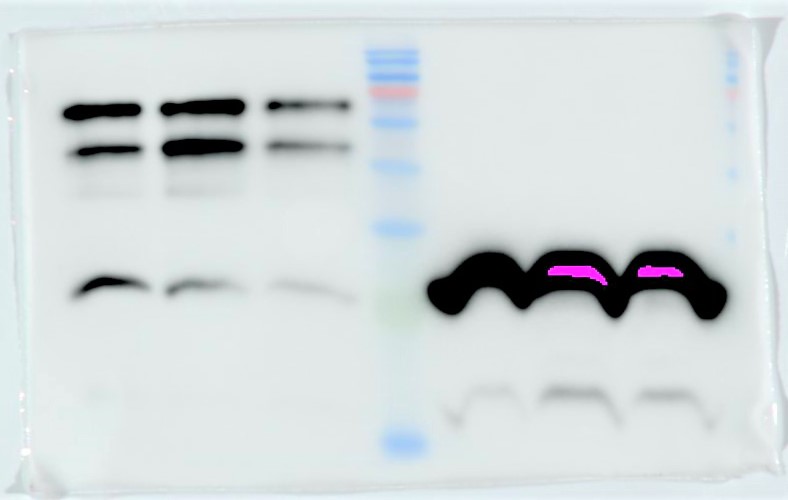

Supplement: Figure 2—source data 1. [file elife-82628-fig2-data1.zip › Figure 2-source data 1/raw unedited gels or blots/Figure 2-source data 1-1.tif]

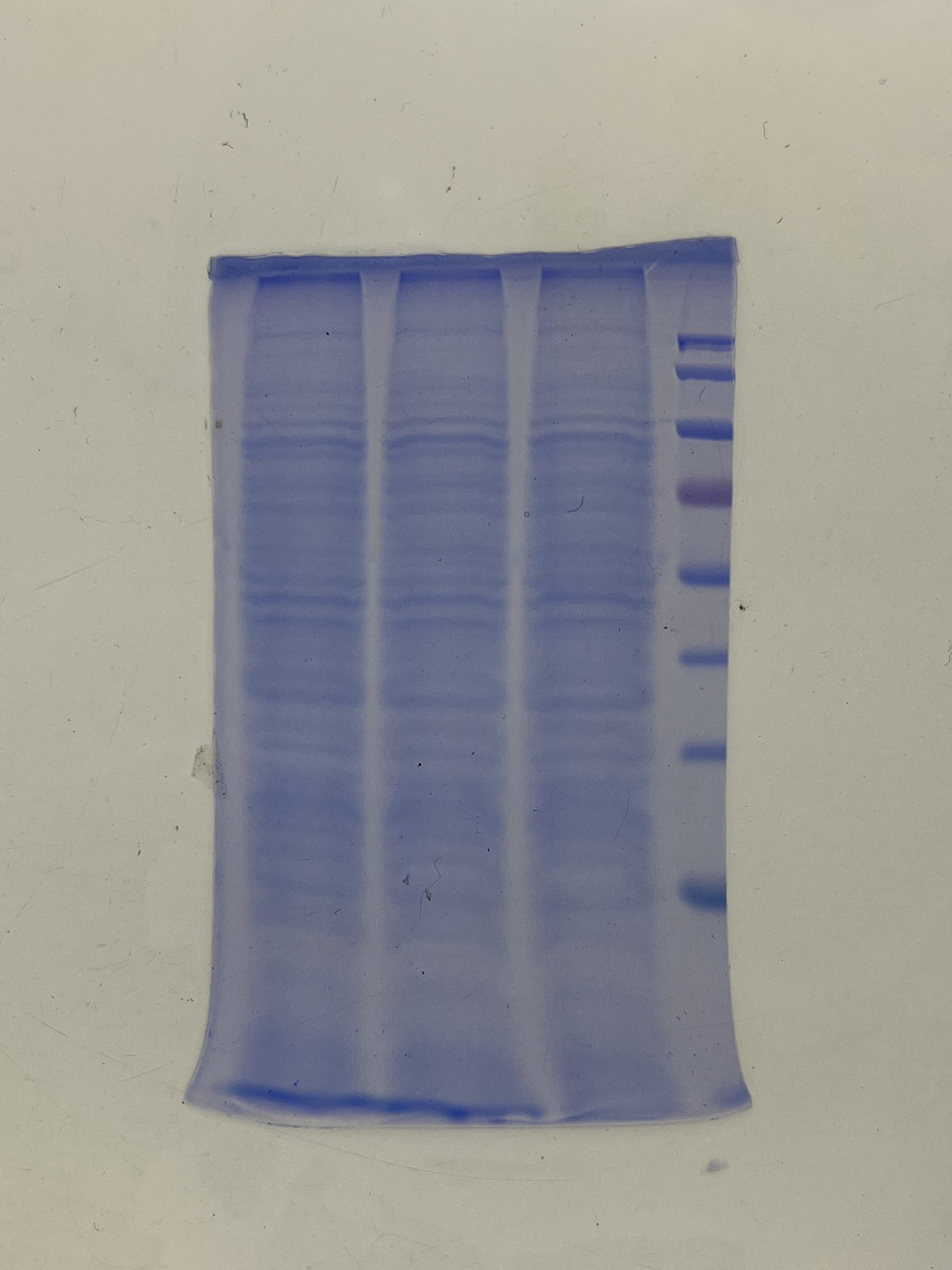

Supplement: Figure 2—source data 1. [file elife-82628-fig2-data1.zip › Figure 2-source data 1/raw unedited gels or blots/Figure 2-source data 1-2.tif]

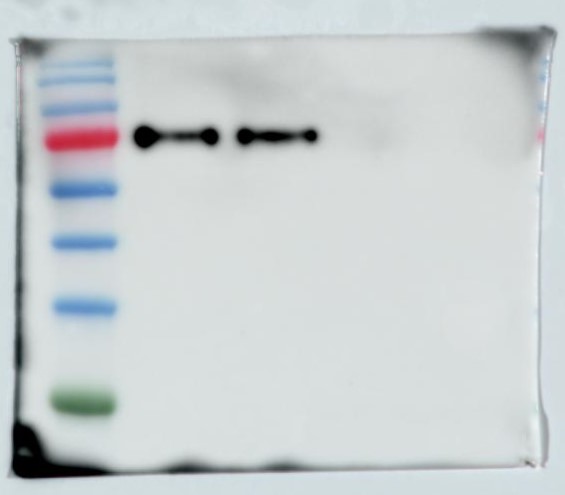

Supplement: Figure 2—source data 1. [file elife-82628-fig2-data1.zip › Figure 2-source data 1/raw unedited gels or blots/Figure 2-source data 1-3.tif]

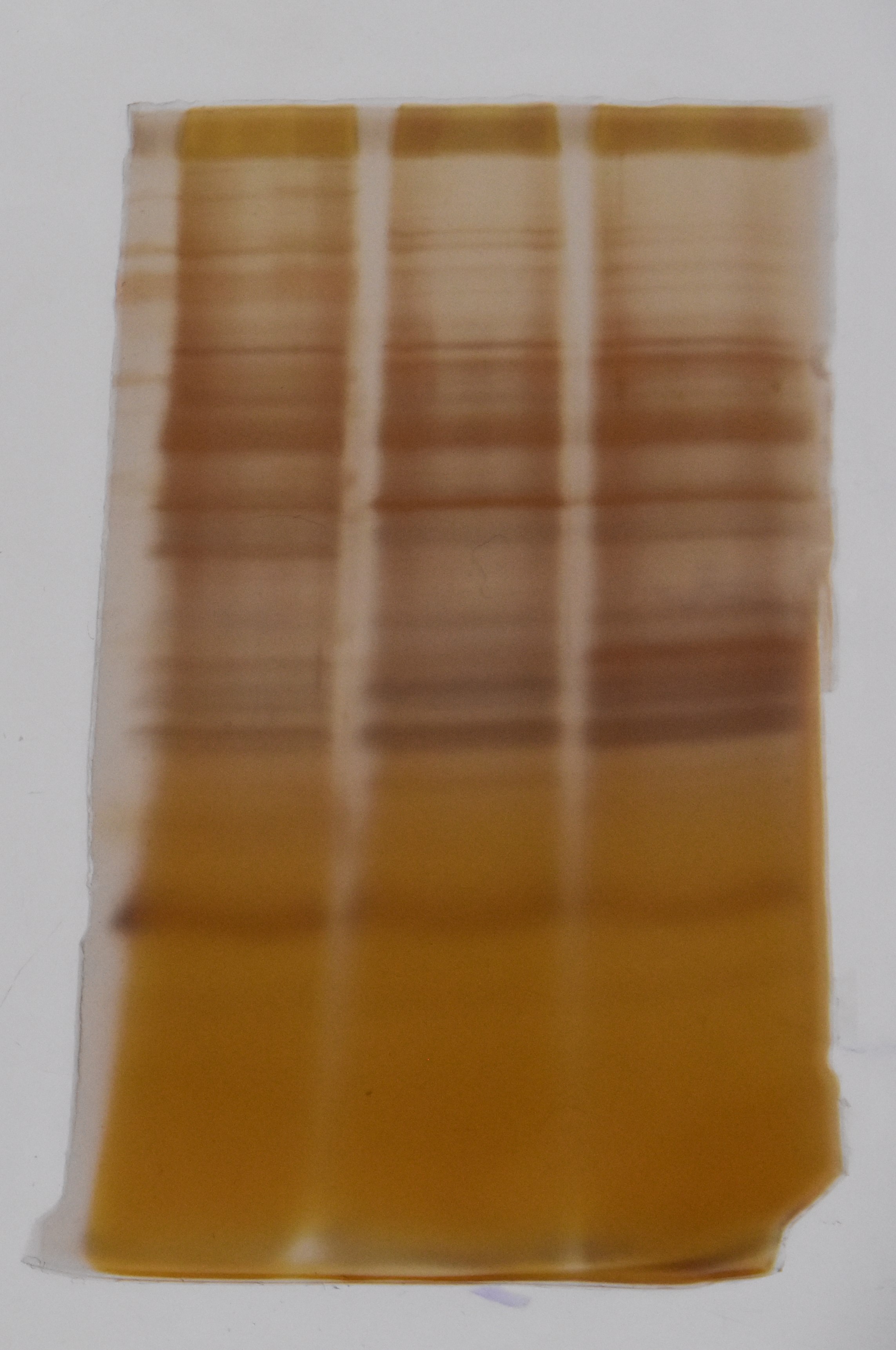

Supplement: Figure 2—source data 1. [file elife-82628-fig2-data1.zip › Figure 2-source data 1/raw unedited gels or blots/Figure 2-source data 1-4.tif]

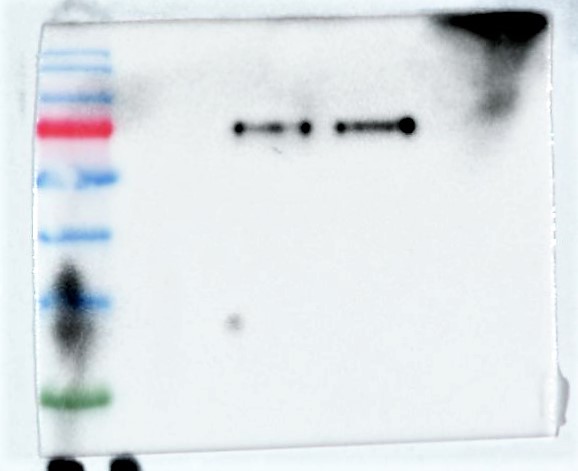

Supplement: Figure 2—source data 1. [file elife-82628-fig2-data1.zip › Figure 2-source data 1/raw unedited gels or blots/Figure 2-source data 1-5.tif]

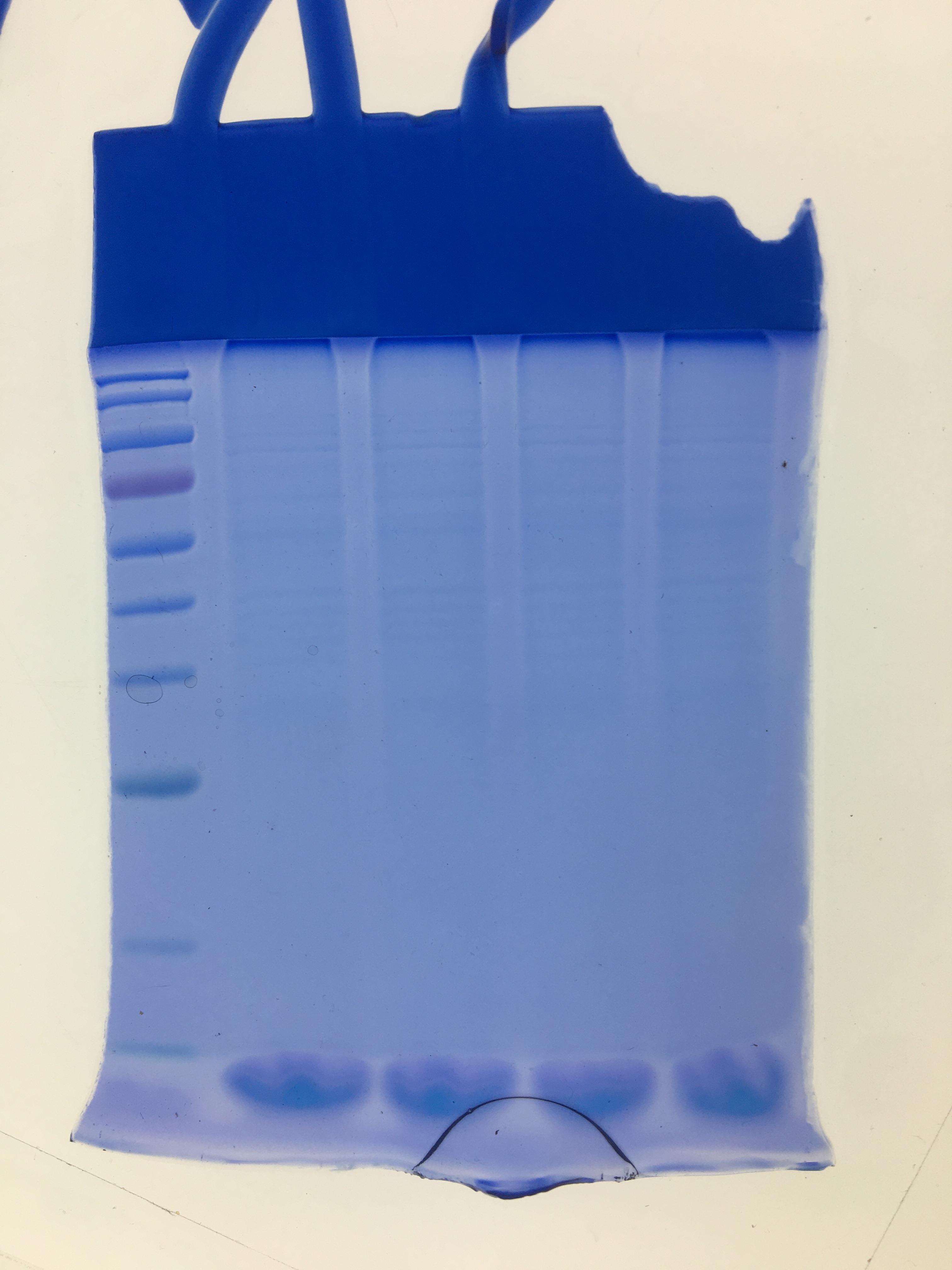

Supplement: Figure 2—source data 1. [file elife-82628-fig2-data1.zip › Figure 2-source data 1/raw unedited gels or blots/Figure 2-source data 1-6.tif]

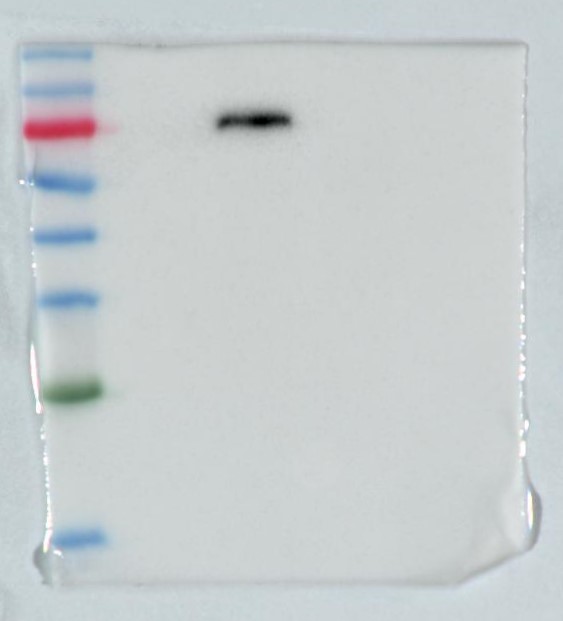

Supplement: Figure 2—source data 1. [file elife-82628-fig2-data1.zip › Figure 2-source data 1/raw unedited gels or blots/Figure 2-source data 1-7.tif]

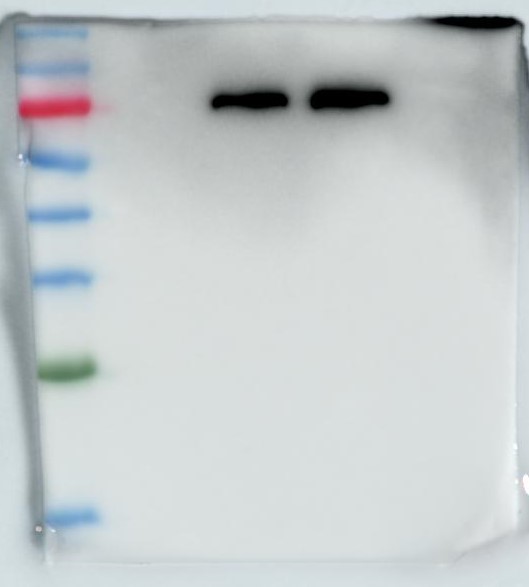

Supplement: Figure 2—source data 1. [file elife-82628-fig2-data1.zip › Figure 2-source data 1/raw unedited gels or blots/Figure 2-source data 1-8.tif]

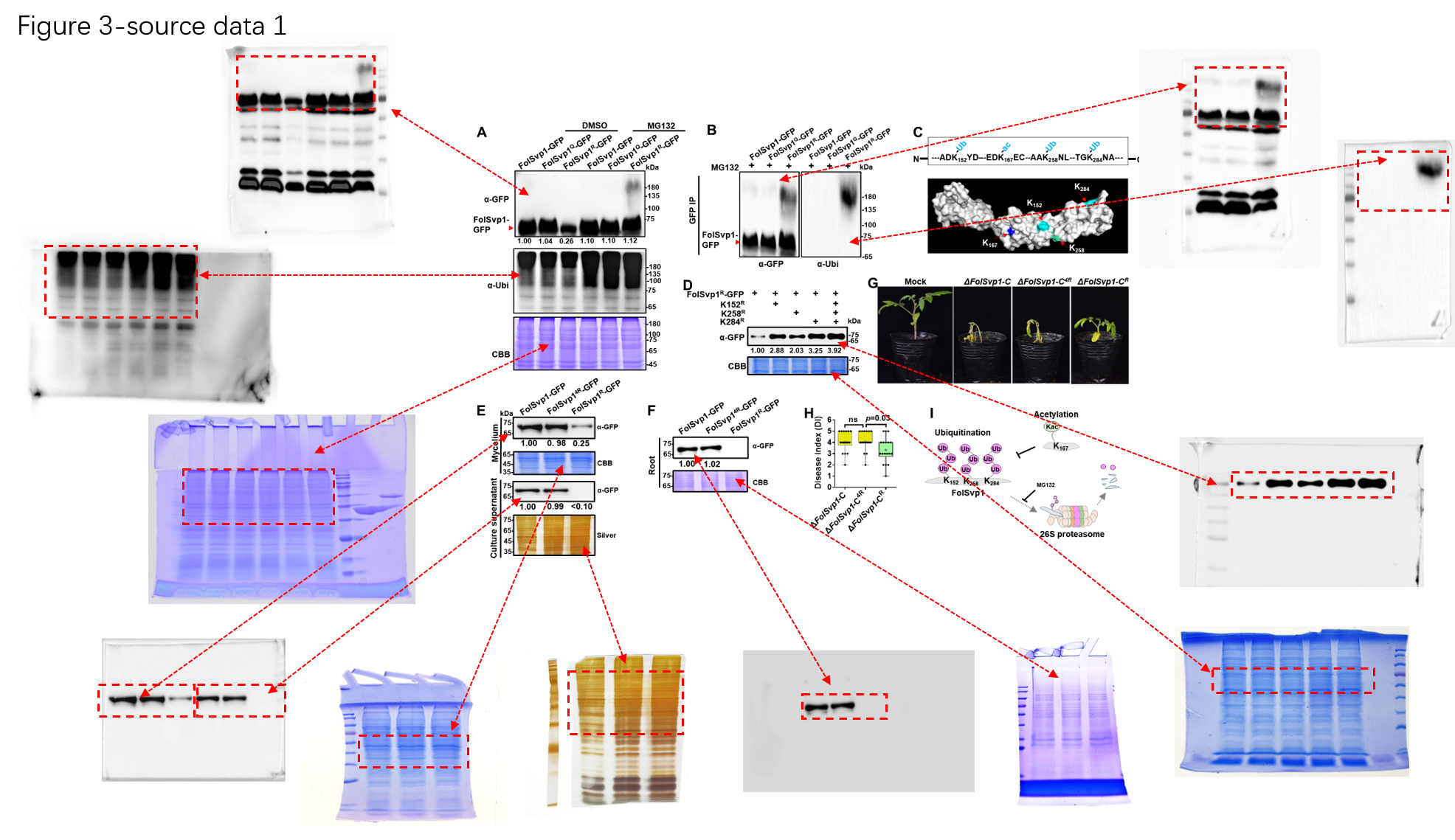

Supplement: Figure 3—source data 1. [file elife-82628-fig3-data1.zip › Figure 3-source data 1/figures with uncropped gels or blots.tif]

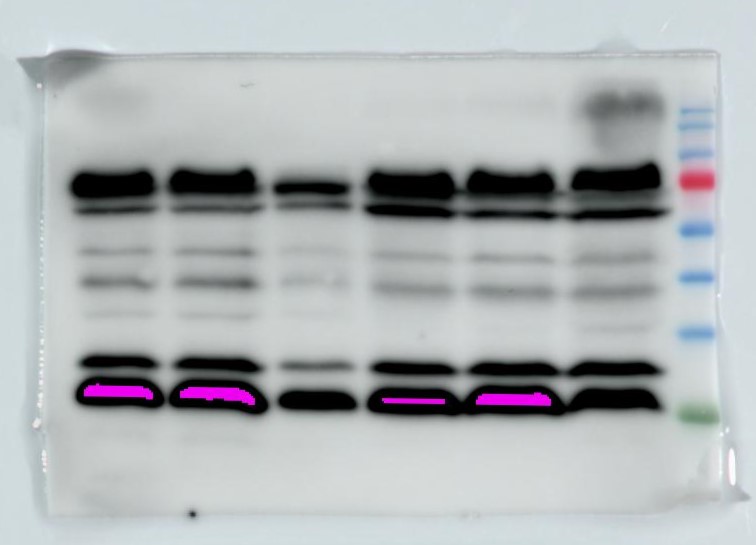

Supplement: Figure 3—source data 1. [file elife-82628-fig3-data1.zip › Figure 3-source data 1/raw unedited gels or blots/Figure 3-source data 1-1.tif]

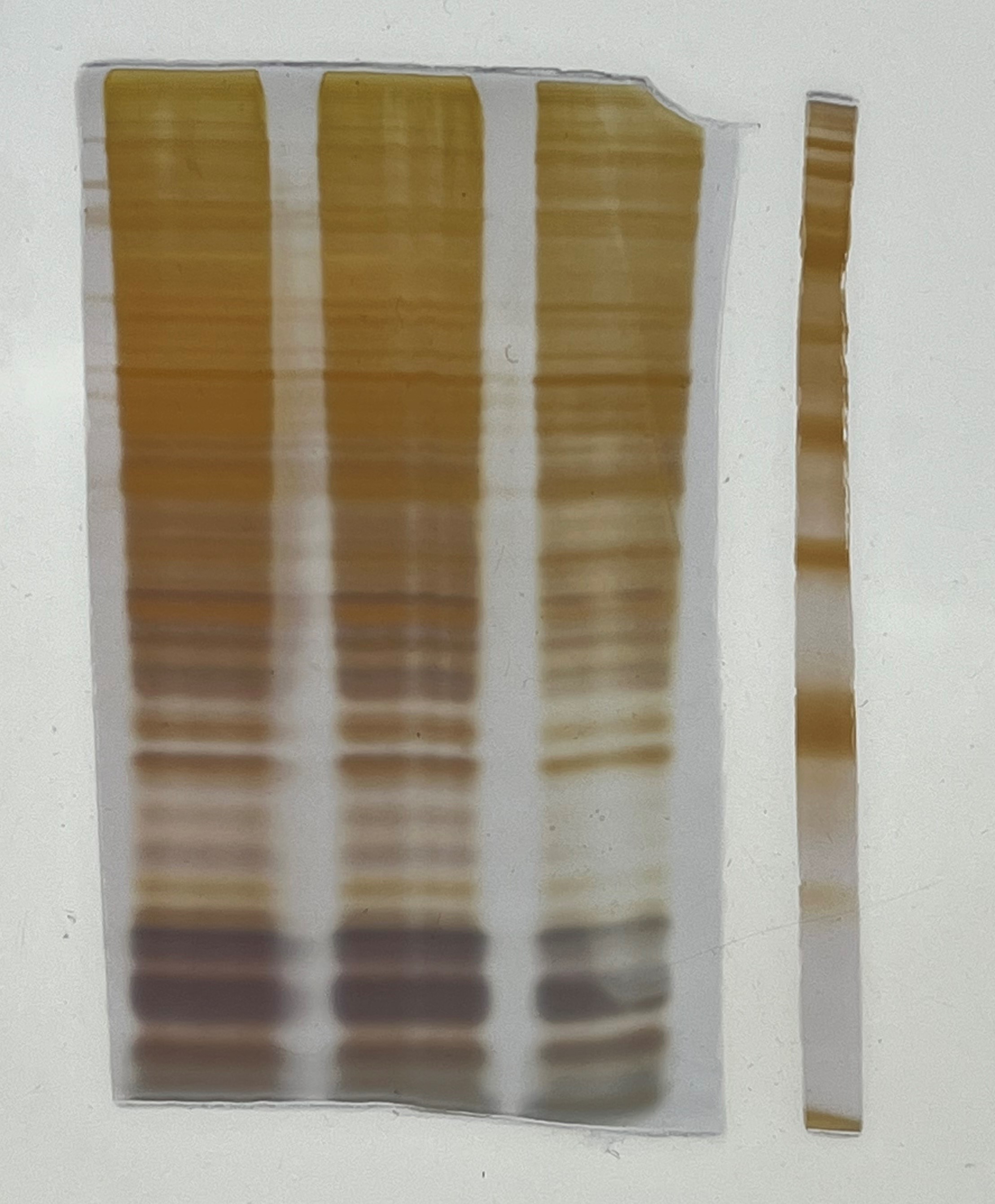

Supplement: Figure 3—source data 1. [file elife-82628-fig3-data1.zip › Figure 3-source data 1/raw unedited gels or blots/Figure 3-source data 1-10.tif]

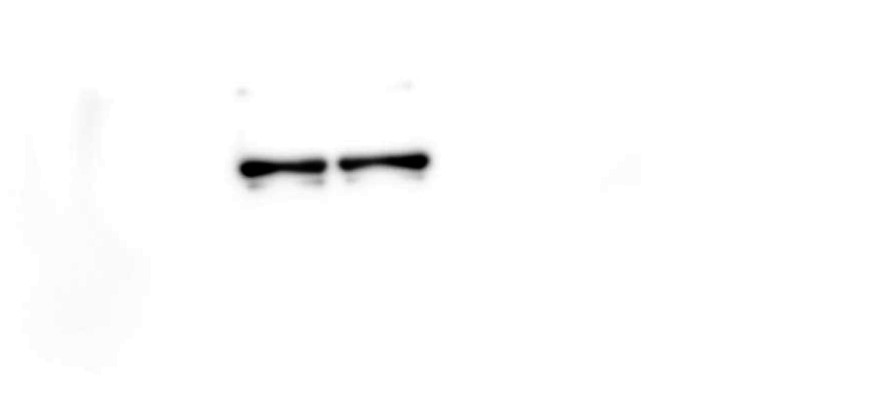

Supplement: Figure 3—source data 1. [file elife-82628-fig3-data1.zip › Figure 3-source data 1/raw unedited gels or blots/Figure 3-source data 1-11.tif]

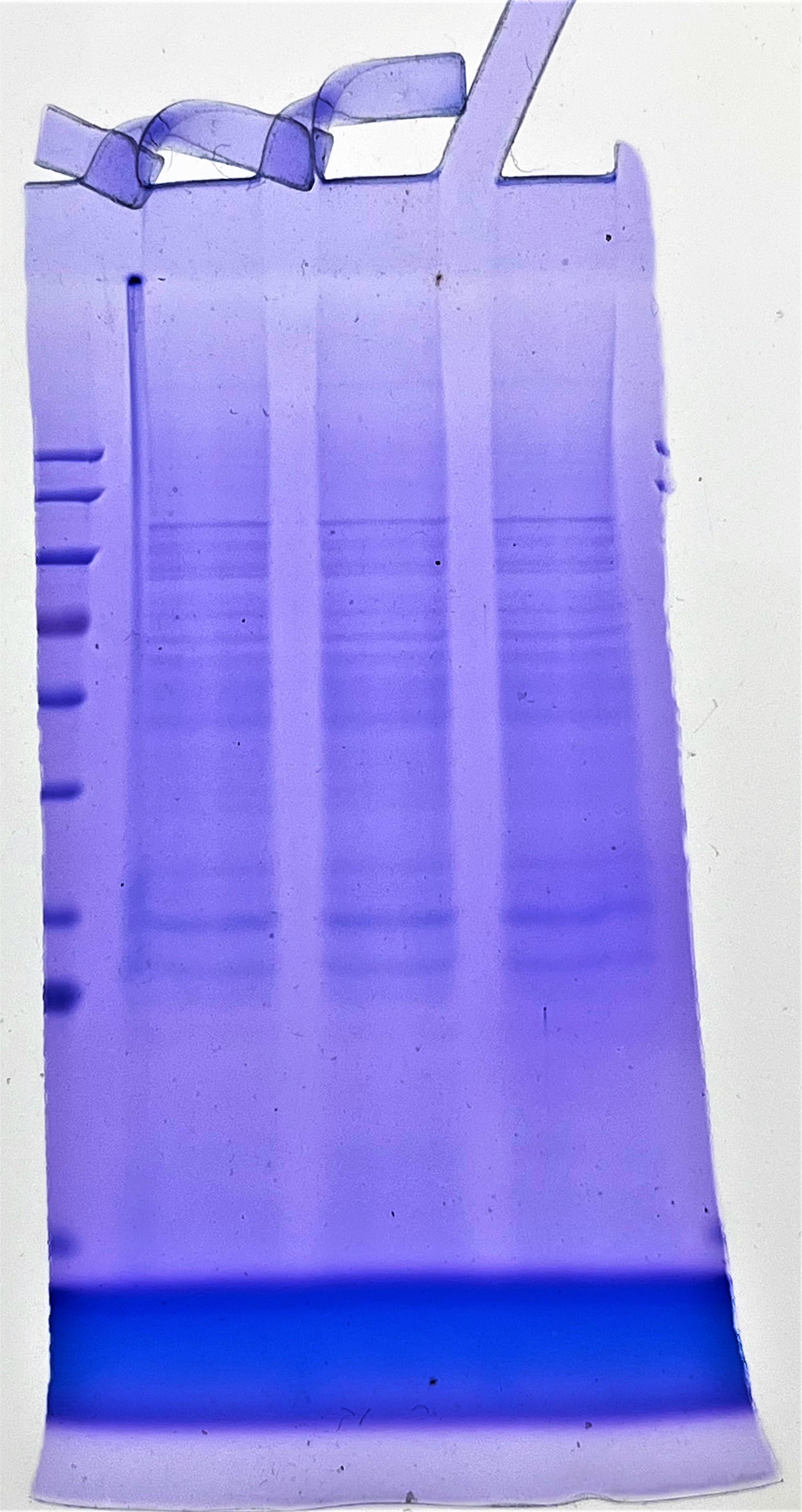

Supplement: Figure 3—source data 1. [file elife-82628-fig3-data1.zip › Figure 3-source data 1/raw unedited gels or blots/Figure 3-source data 1-12.tif]

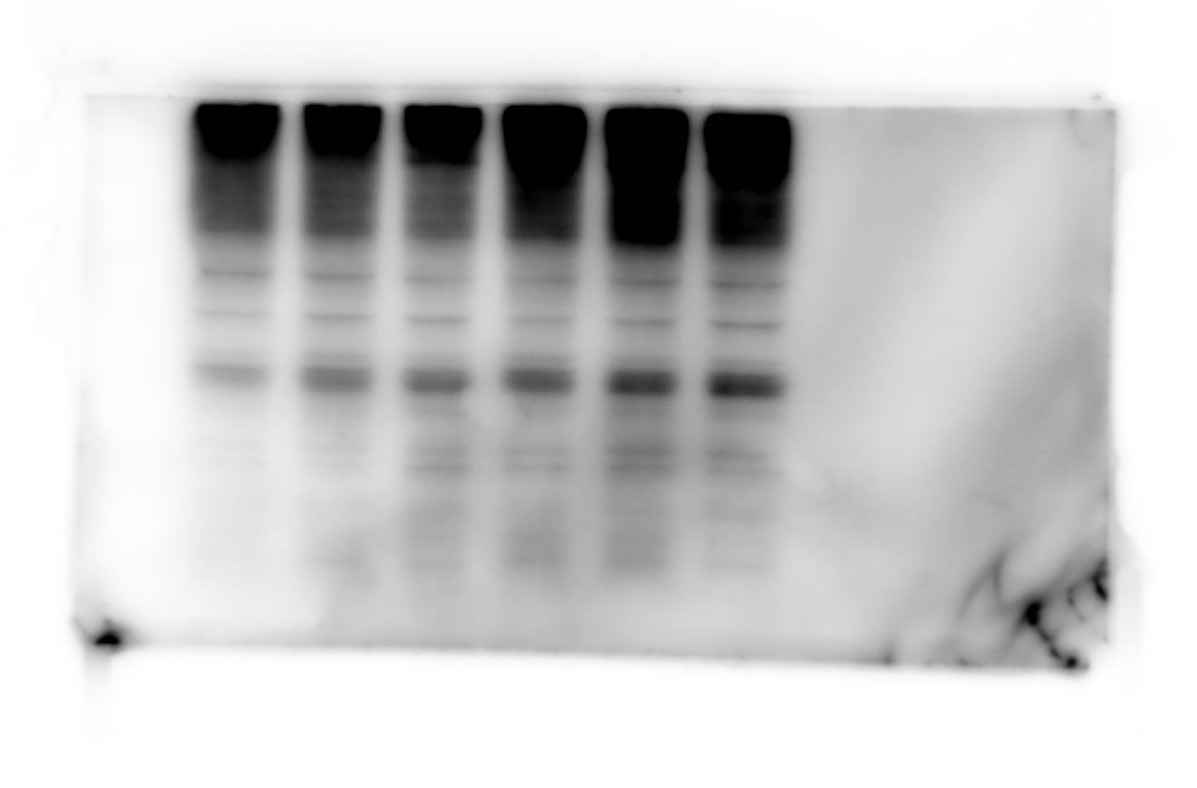

Supplement: Figure 3—source data 1. [file elife-82628-fig3-data1.zip › Figure 3-source data 1/raw unedited gels or blots/Figure 3-source data 1-2.tif]

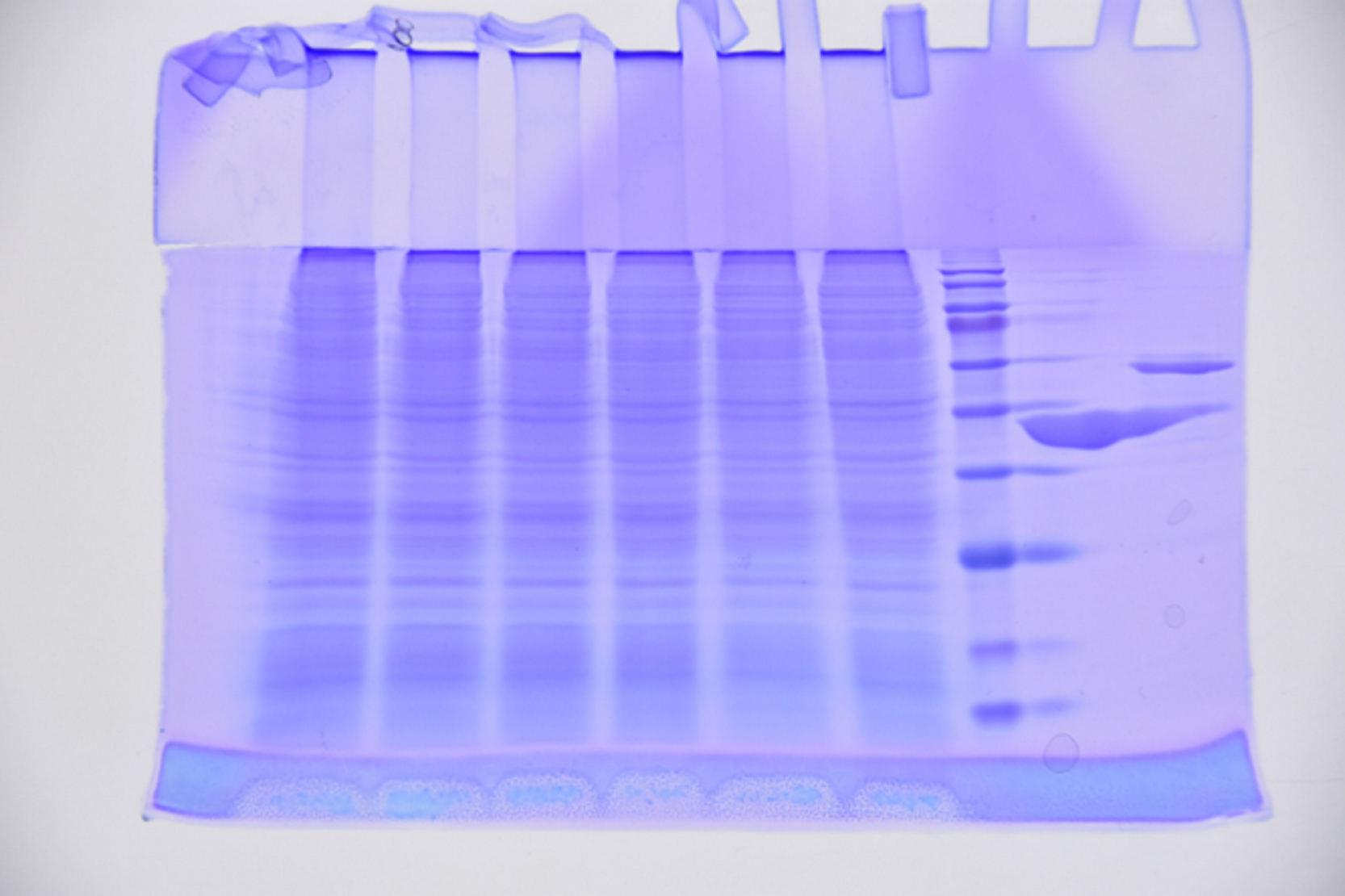

Supplement: Figure 3—source data 1. [file elife-82628-fig3-data1.zip › Figure 3-source data 1/raw unedited gels or blots/Figure 3-source data 1-3.tif]

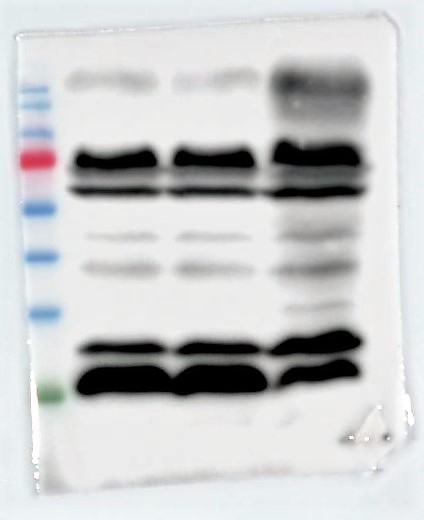

Supplement: Figure 3—source data 1. [file elife-82628-fig3-data1.zip › Figure 3-source data 1/raw unedited gels or blots/Figure 3-source data 1-4.tif]

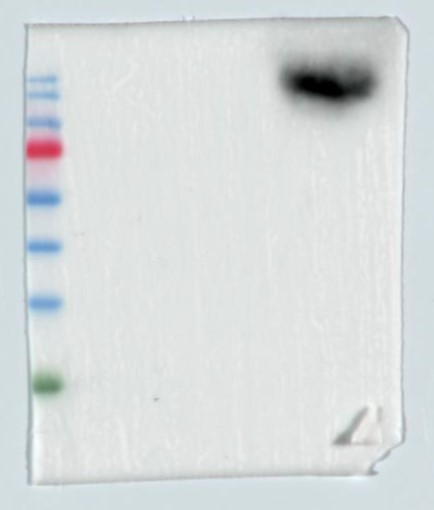

Supplement: Figure 3—source data 1. [file elife-82628-fig3-data1.zip › Figure 3-source data 1/raw unedited gels or blots/Figure 3-source data 1-5.tif]

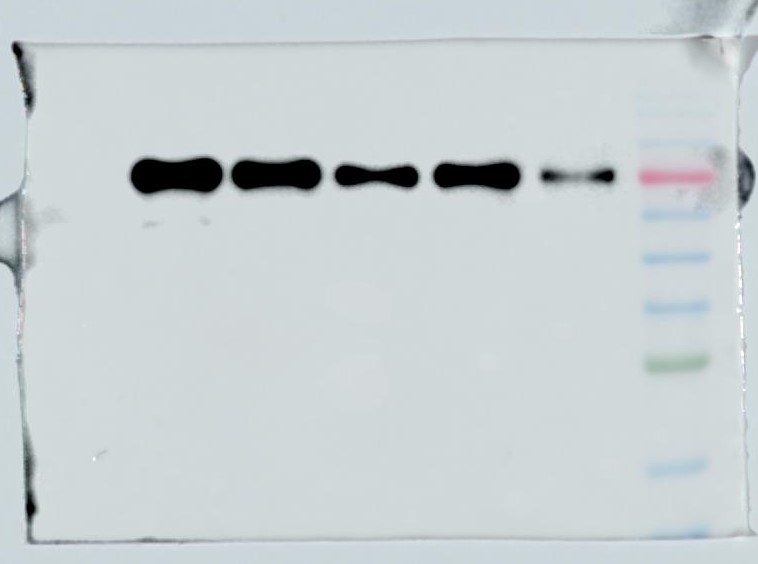

Supplement: Figure 3—source data 1. [file elife-82628-fig3-data1.zip › Figure 3-source data 1/raw unedited gels or blots/Figure 3-source data 1-6.tif]

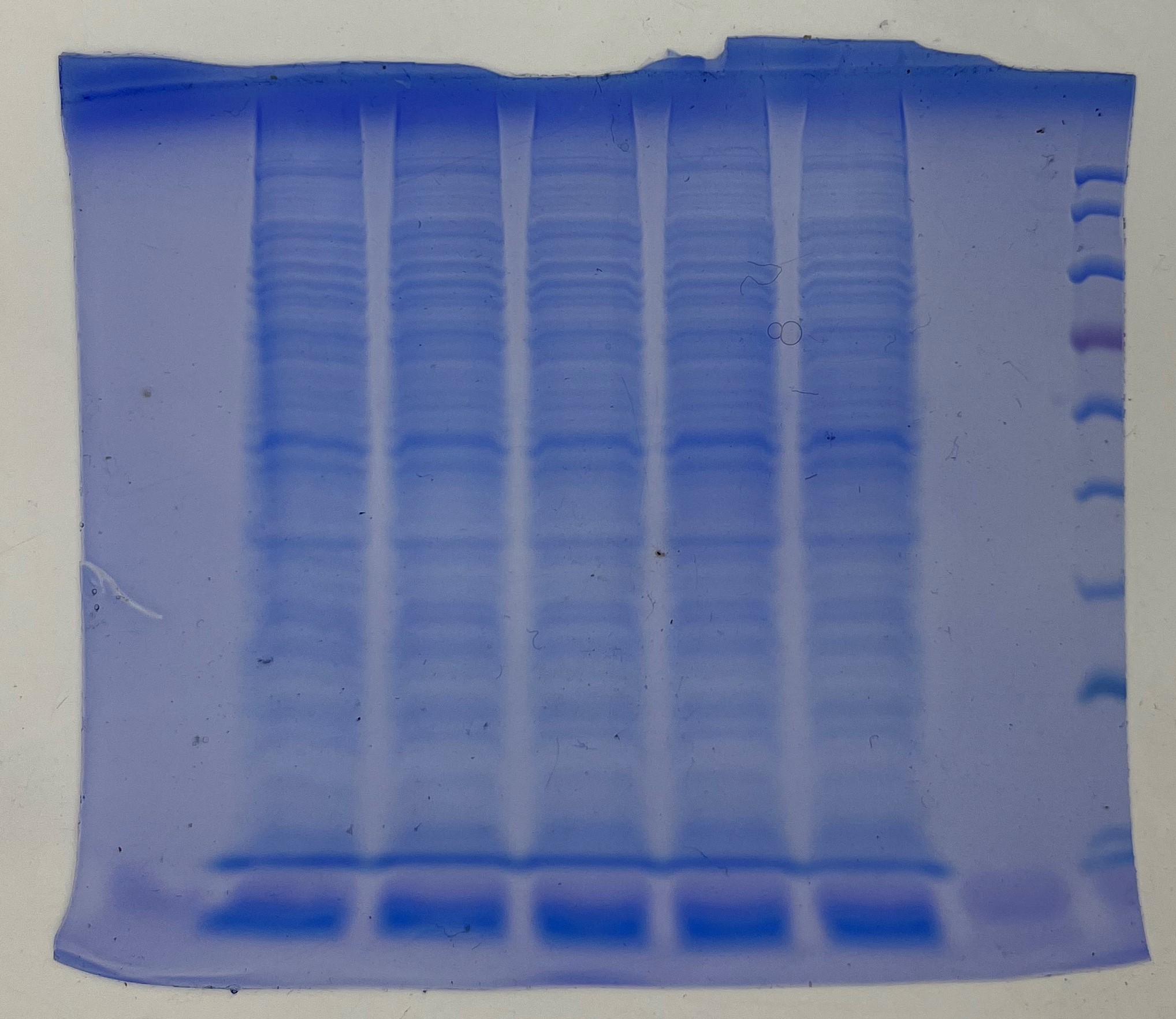

Supplement: Figure 3—source data 1. [file elife-82628-fig3-data1.zip › Figure 3-source data 1/raw unedited gels or blots/Figure 3-source data 1-7.tif]

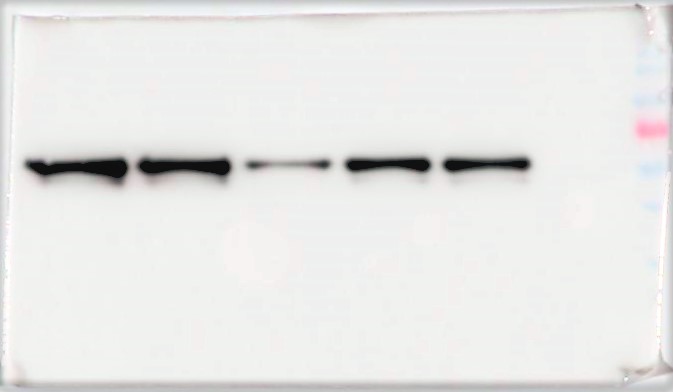

Supplement: Figure 3—source data 1. [file elife-82628-fig3-data1.zip › Figure 3-source data 1/raw unedited gels or blots/Figure 3-source data 1-8.tif]

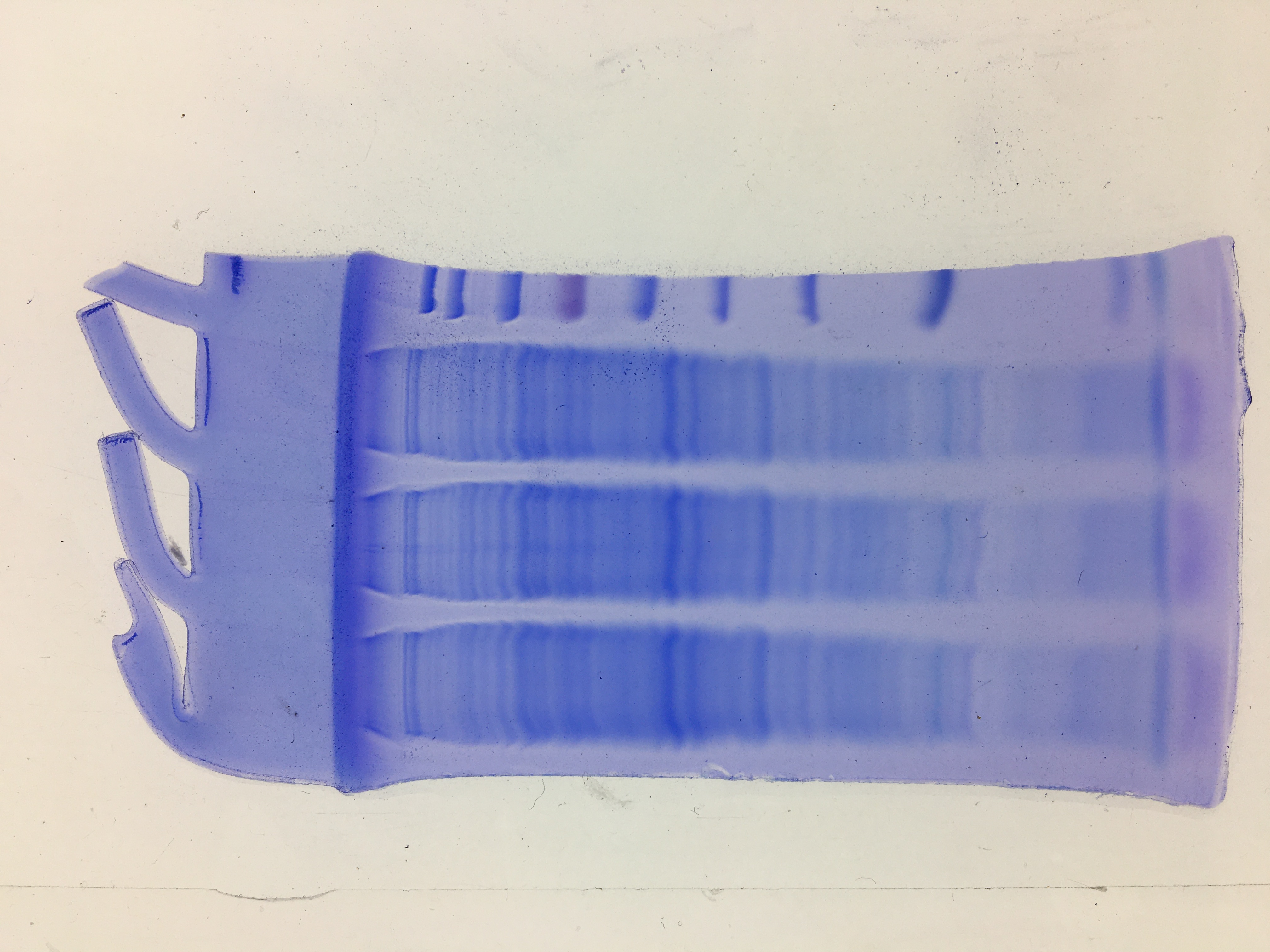

Supplement: Figure 3—source data 1. [file elife-82628-fig3-data1.zip › Figure 3-source data 1/raw unedited gels or blots/Figure 3-source data 1-9.tif]

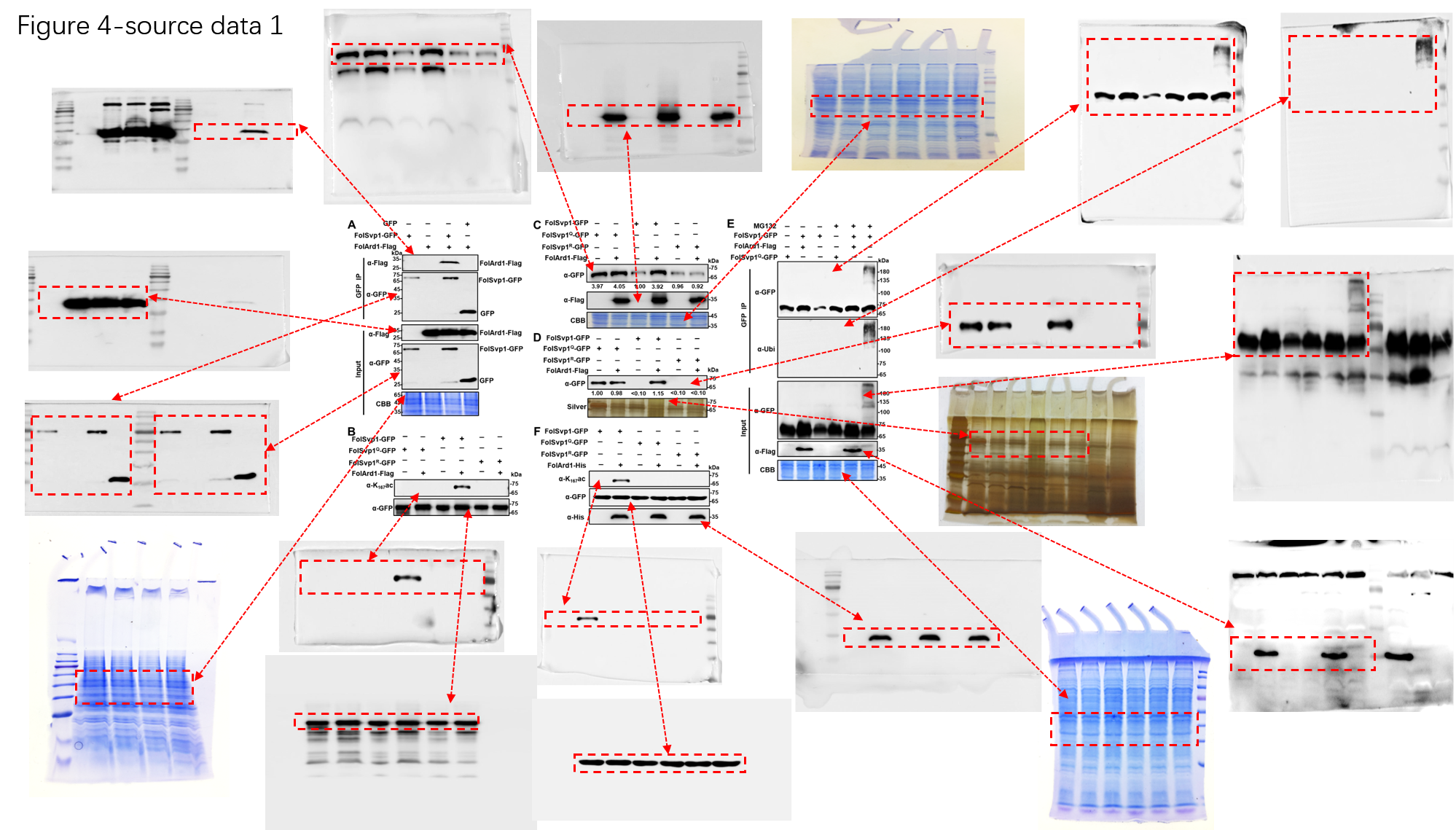

Supplement: Figure 4—source data 1. [file elife-82628-fig4-data1.zip › Figure 4-source data 1/figures with uncropped gels or blots.tif]

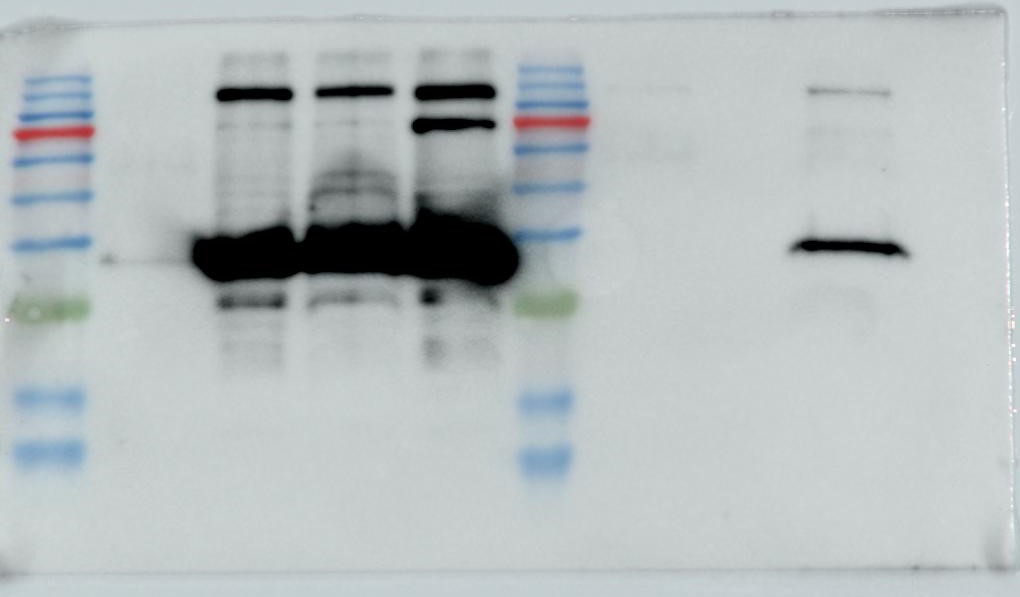

Supplement: Figure 4—source data 1. [file elife-82628-fig4-data1.zip › Figure 4-source data 1/raw unedited gels or blots/Figure 4-source data 1-1.tif]

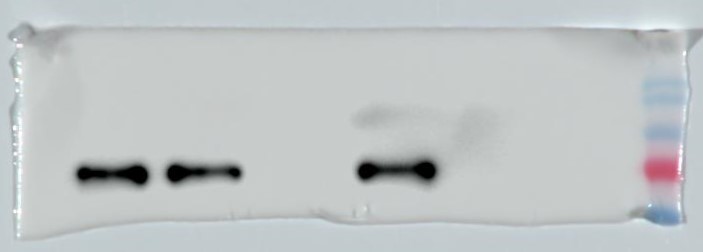

Supplement: Figure 4—source data 1. [file elife-82628-fig4-data1.zip › Figure 4-source data 1/raw unedited gels or blots/Figure 4-source data 1-10.tif]

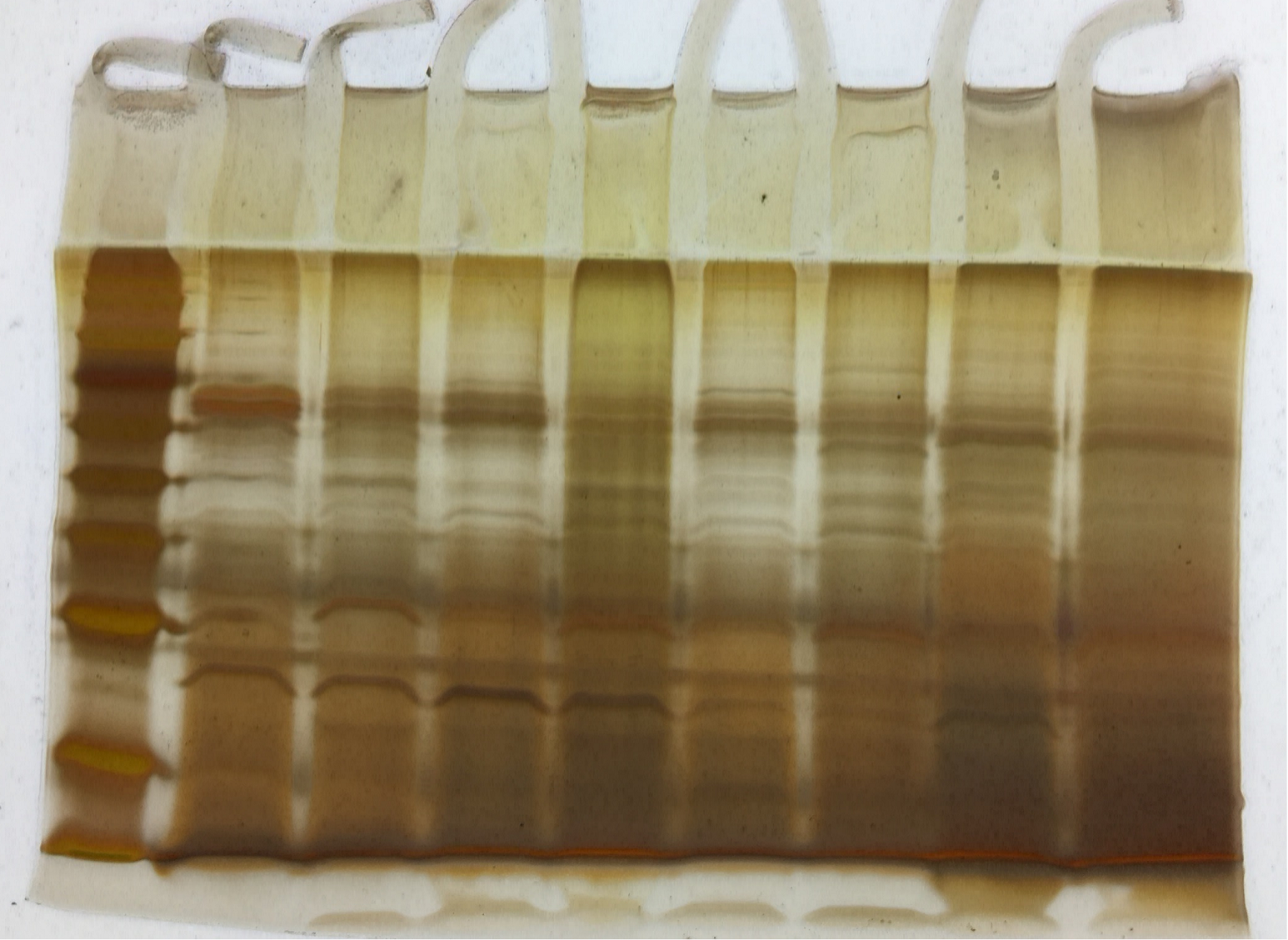

Supplement: Figure 4—source data 1. [file elife-82628-fig4-data1.zip › Figure 4-source data 1/raw unedited gels or blots/Figure 4-source data 1-11.tif]

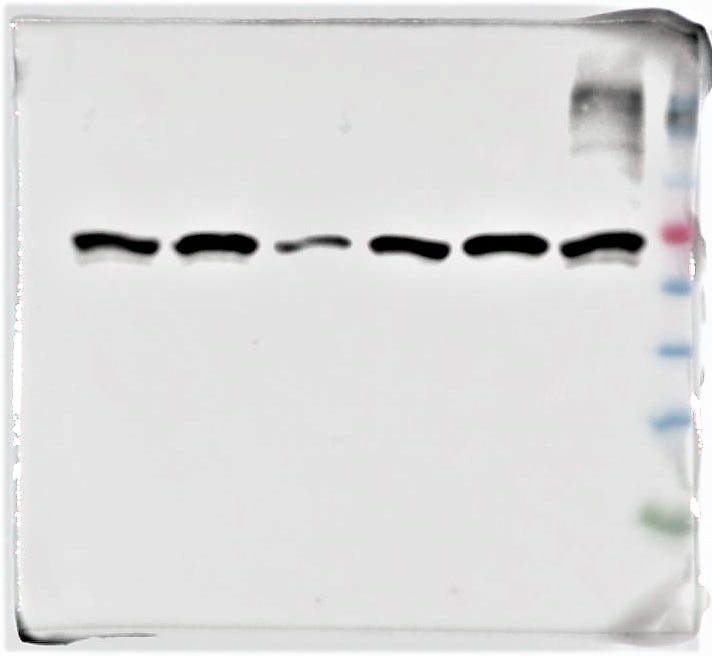

Supplement: Figure 4—source data 1. [file elife-82628-fig4-data1.zip › Figure 4-source data 1/raw unedited gels or blots/Figure 4-source data 1-12.tif]

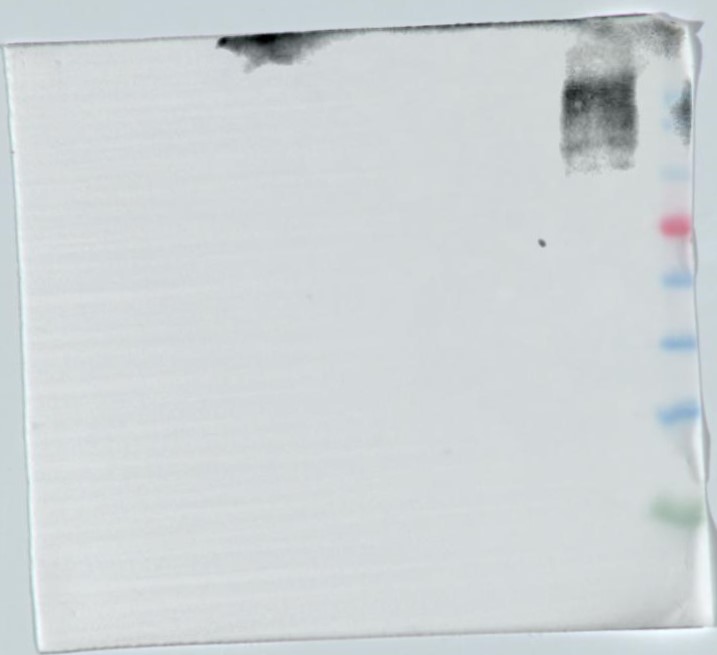

Supplement: Figure 4—source data 1. [file elife-82628-fig4-data1.zip › Figure 4-source data 1/raw unedited gels or blots/Figure 4-source data 1-13.tif]

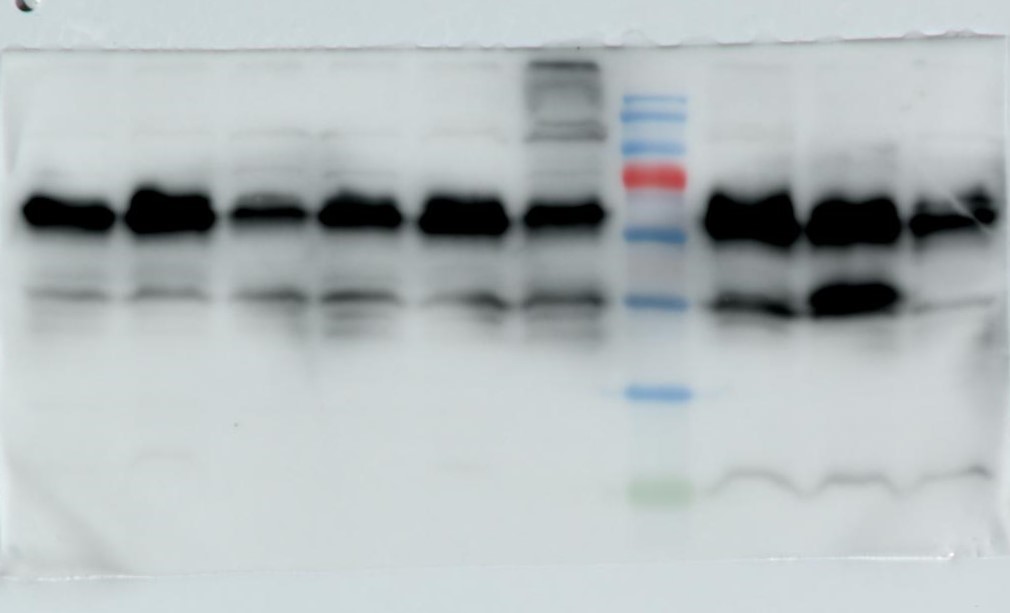

Supplement: Figure 4—source data 1. [file elife-82628-fig4-data1.zip › Figure 4-source data 1/raw unedited gels or blots/Figure 4-source data 1-14.tif]

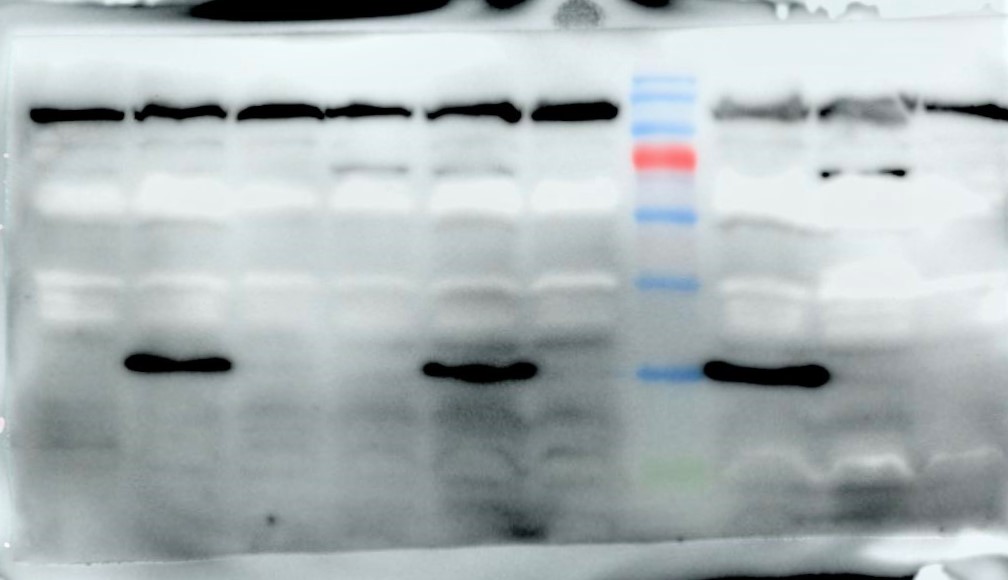

Supplement: Figure 4—source data 1. [file elife-82628-fig4-data1.zip › Figure 4-source data 1/raw unedited gels or blots/Figure 4-source data 1-15.tif]

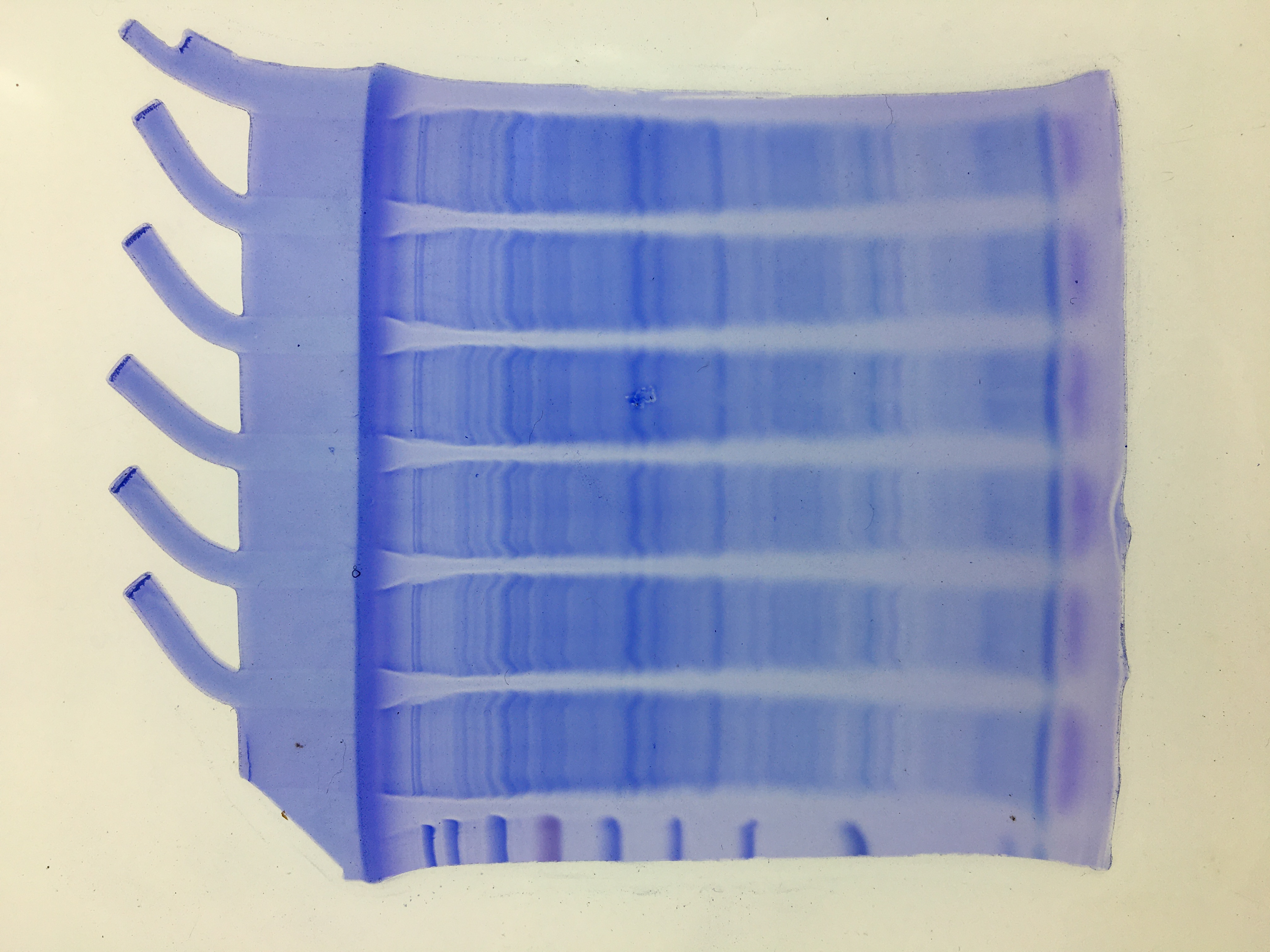

Supplement: Figure 4—source data 1. [file elife-82628-fig4-data1.zip › Figure 4-source data 1/raw unedited gels or blots/Figure 4-source data 1-16.tif]

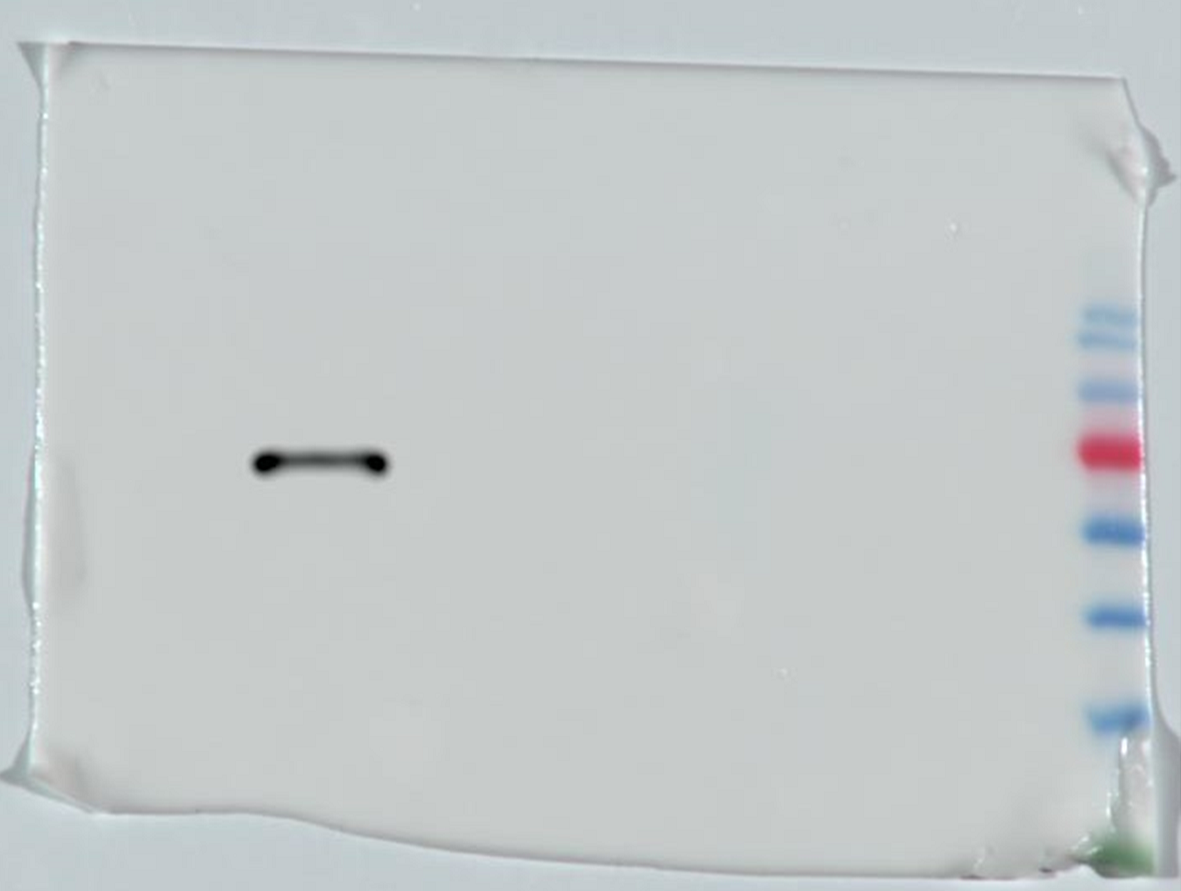

Supplement: Figure 4—source data 1. [file elife-82628-fig4-data1.zip › Figure 4-source data 1/raw unedited gels or blots/Figure 4-source data 1-17.tif]

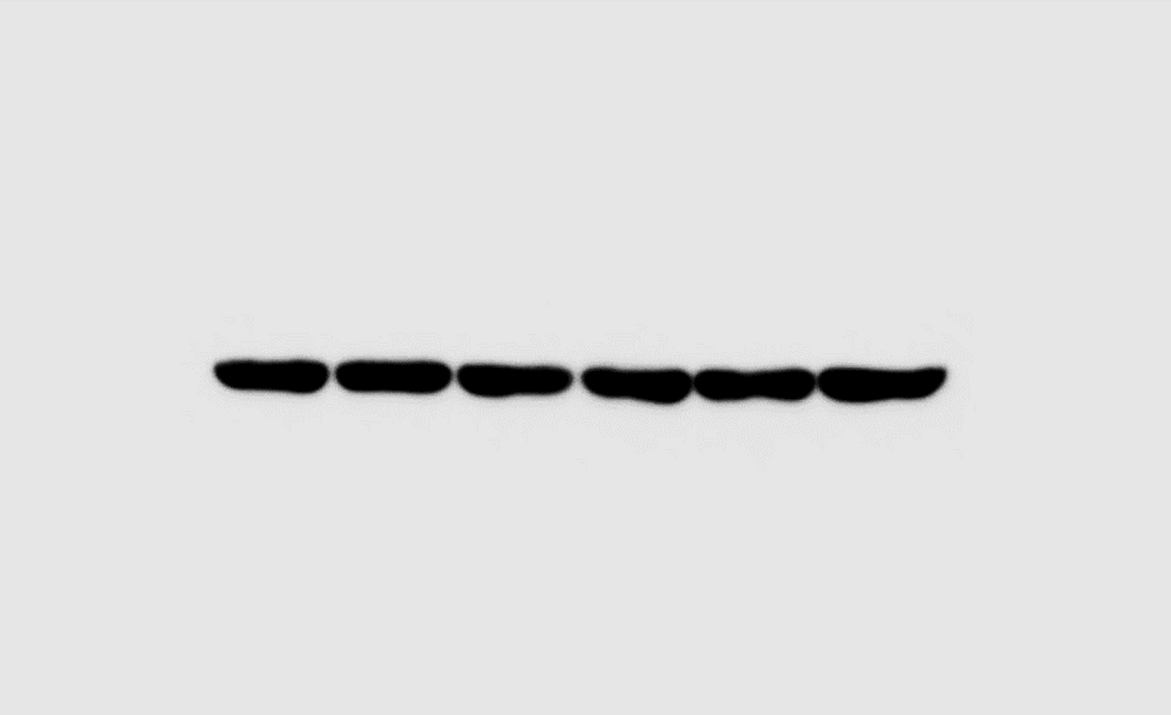

Supplement: Figure 4—source data 1. [file elife-82628-fig4-data1.zip › Figure 4-source data 1/raw unedited gels or blots/Figure 4-source data 1-18.tif]

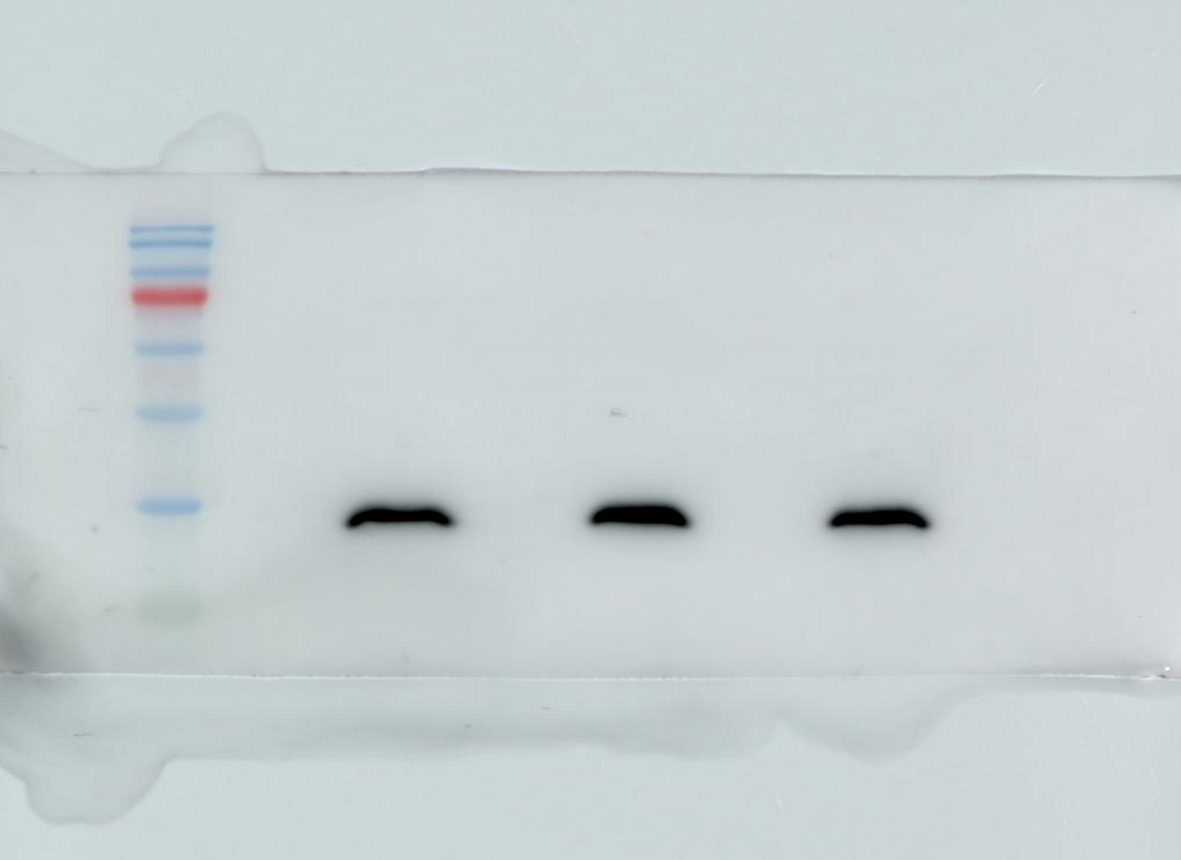

Supplement: Figure 4—source data 1. [file elife-82628-fig4-data1.zip › Figure 4-source data 1/raw unedited gels or blots/Figure 4-source data 1-19.tif]

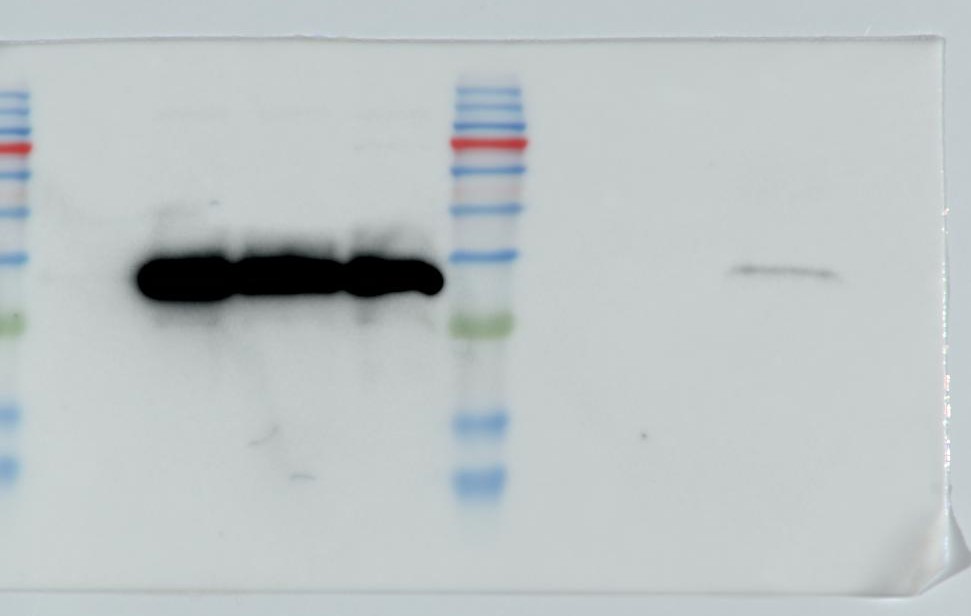

Supplement: Figure 4—source data 1. [file elife-82628-fig4-data1.zip › Figure 4-source data 1/raw unedited gels or blots/Figure 4-source data 1-2.tif]

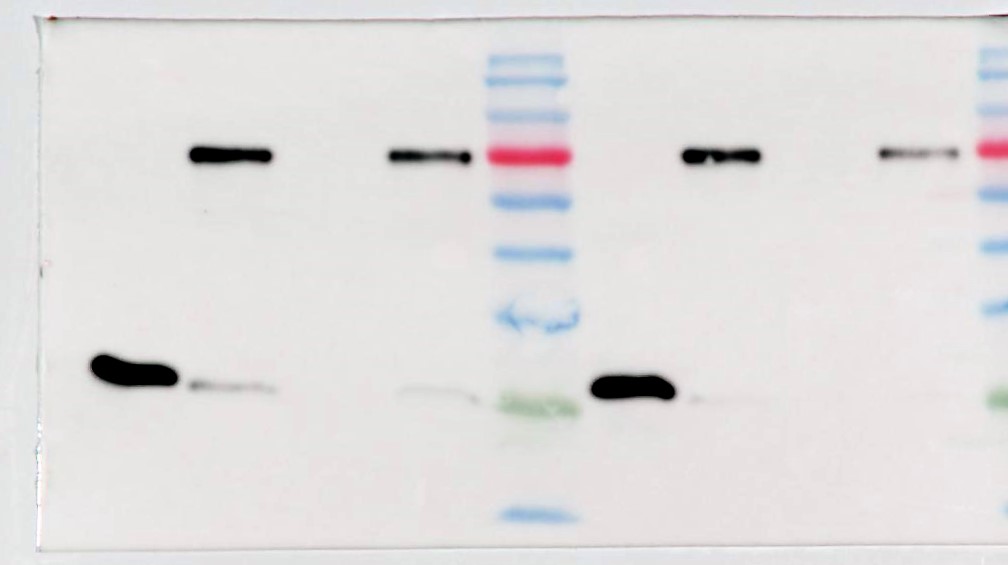

Supplement: Figure 4—source data 1. [file elife-82628-fig4-data1.zip › Figure 4-source data 1/raw unedited gels or blots/Figure 4-source data 1-3.tif]

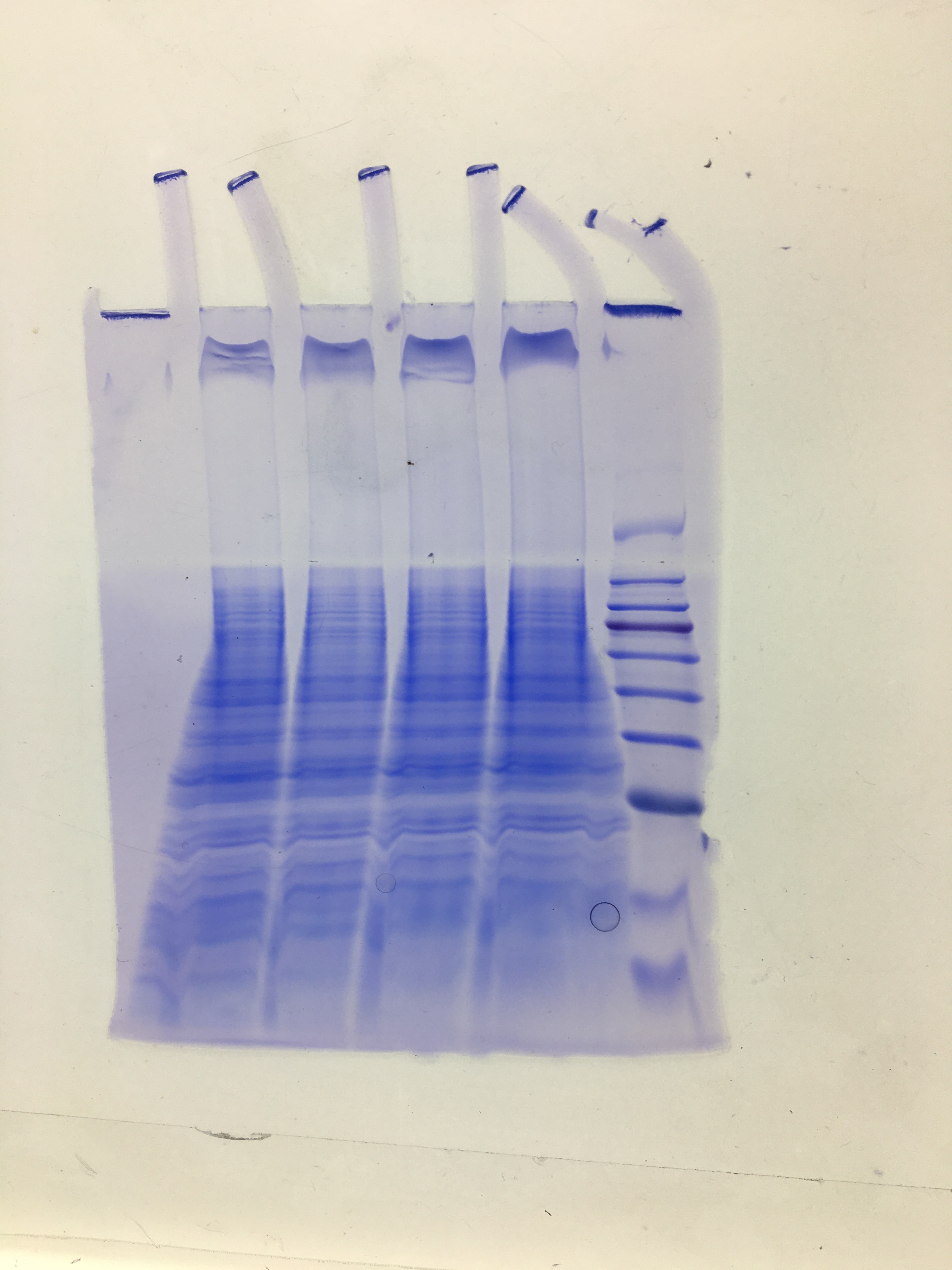

Supplement: Figure 4—source data 1. [file elife-82628-fig4-data1.zip › Figure 4-source data 1/raw unedited gels or blots/Figure 4-source data 1-4.tif]

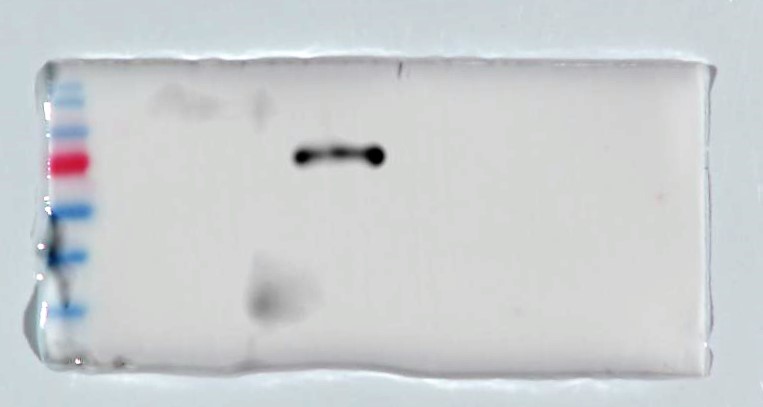

Supplement: Figure 4—source data 1. [file elife-82628-fig4-data1.zip › Figure 4-source data 1/raw unedited gels or blots/Figure 4-source data 1-5.tif]

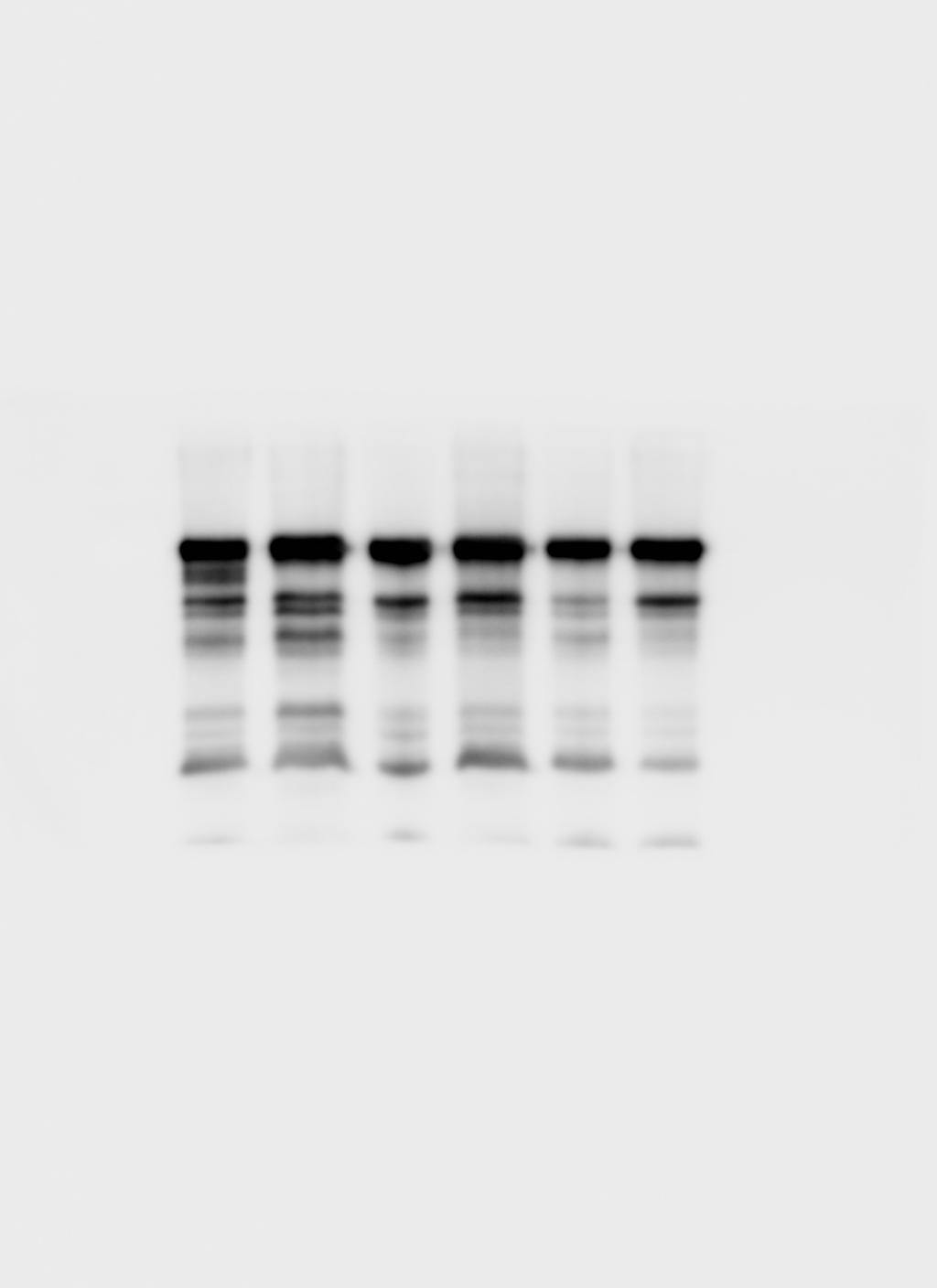

Supplement: Figure 4—source data 1. [file elife-82628-fig4-data1.zip › Figure 4-source data 1/raw unedited gels or blots/Figure 4-source data 1-6.tif]

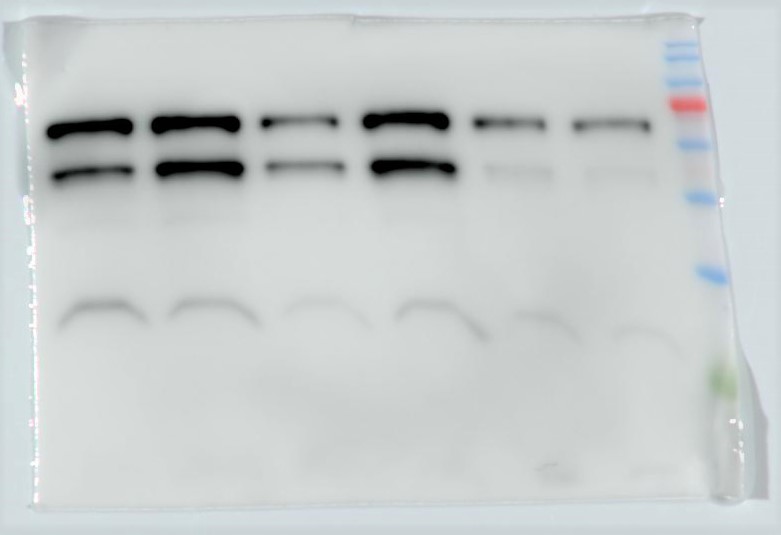

Supplement: Figure 4—source data 1. [file elife-82628-fig4-data1.zip › Figure 4-source data 1/raw unedited gels or blots/Figure 4-source data 1-7.tif]

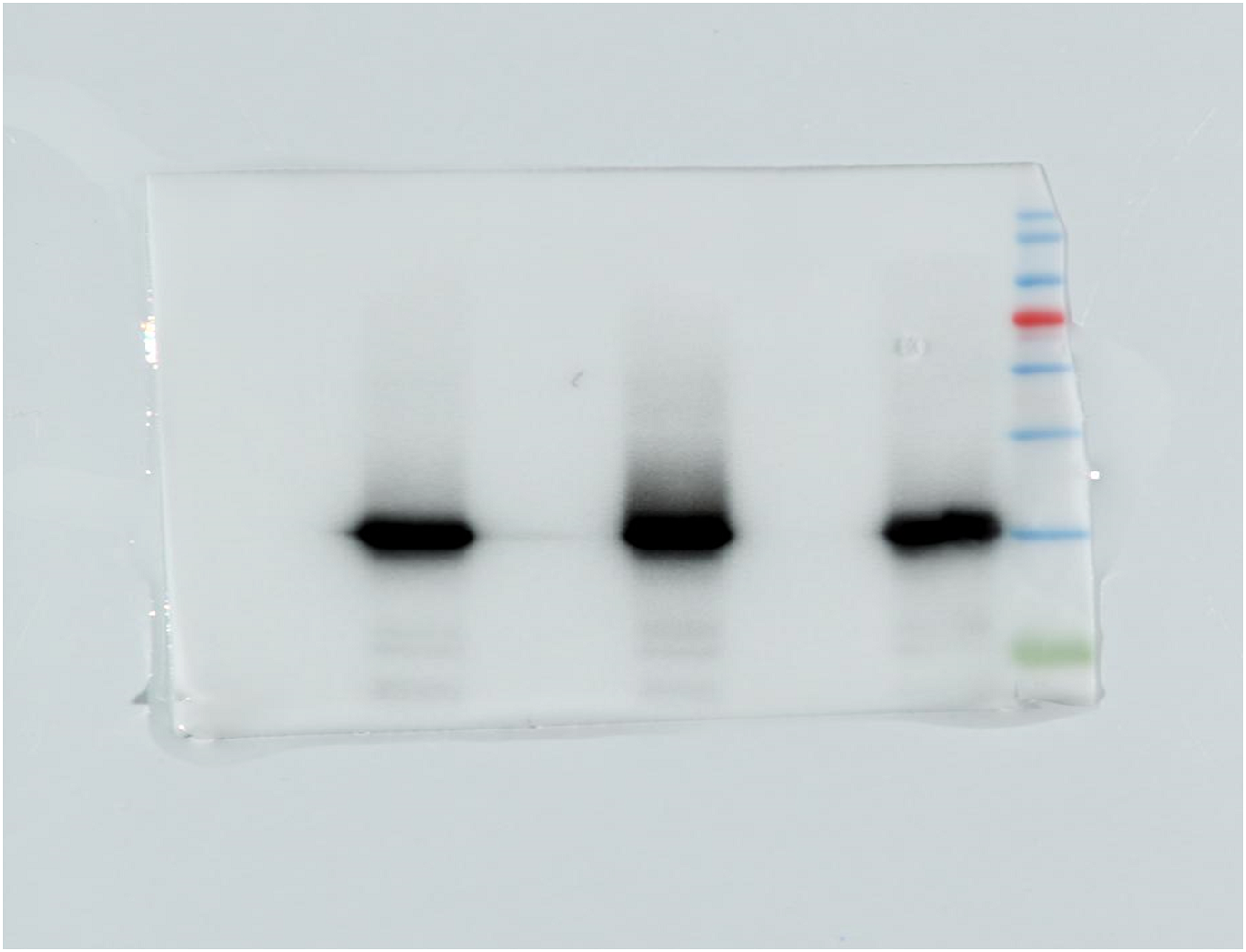

Supplement: Figure 4—source data 1. [file elife-82628-fig4-data1.zip › Figure 4-source data 1/raw unedited gels or blots/Figure 4-source data 1-8.tif]

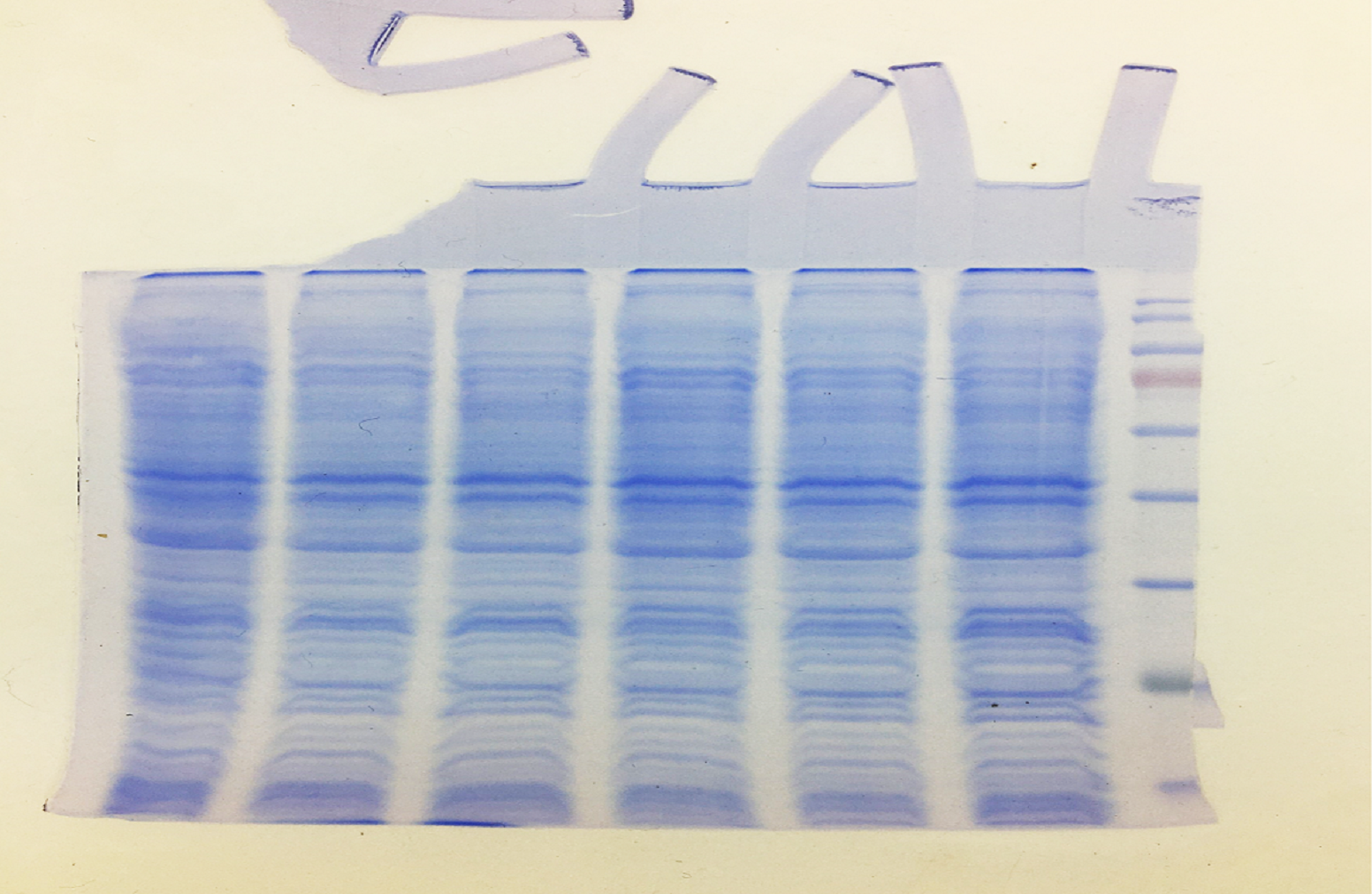

Supplement: Figure 4—source data 1. [file elife-82628-fig4-data1.zip › Figure 4-source data 1/raw unedited gels or blots/Figure 4-source data 1-9.tif]

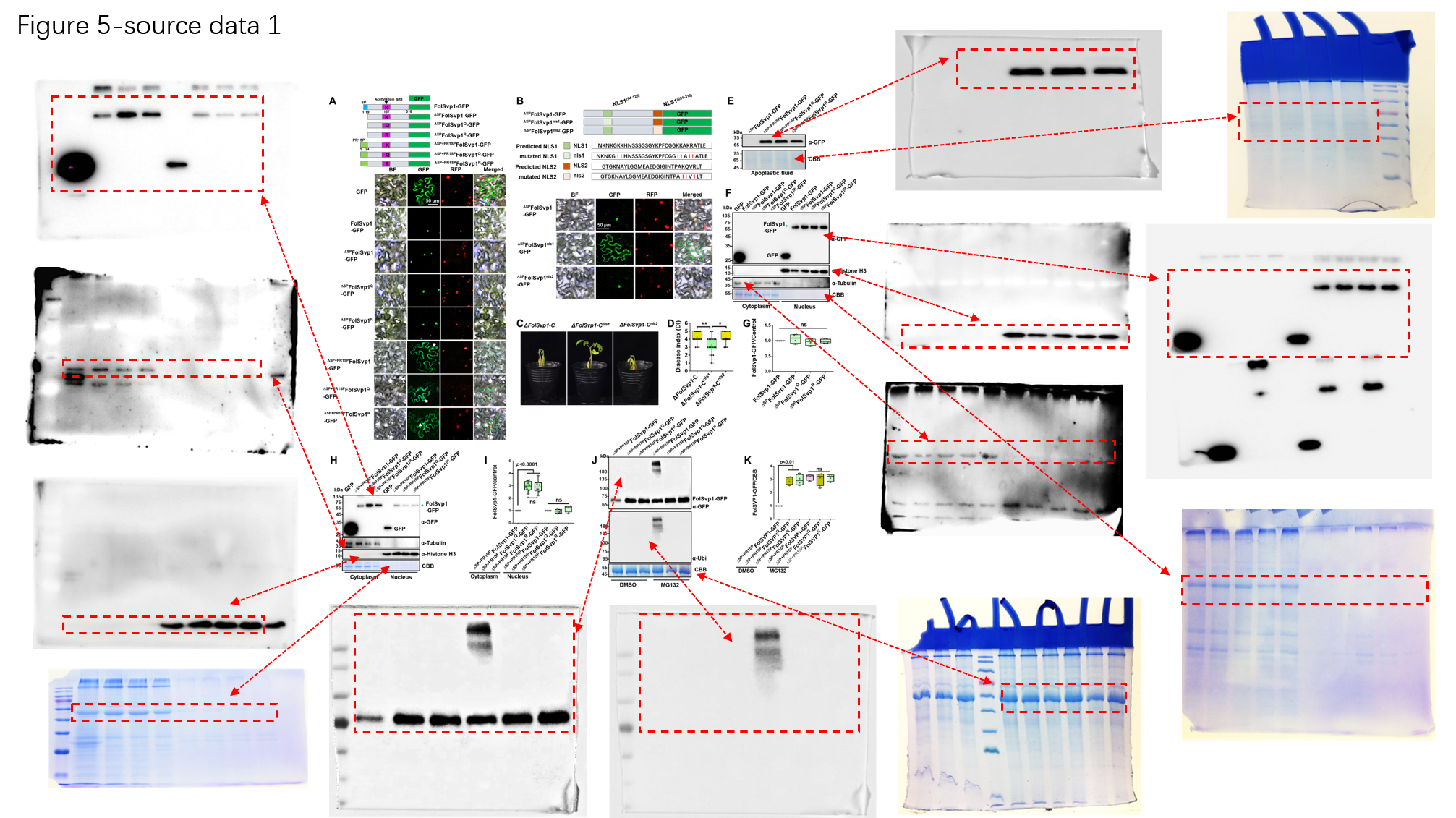

Supplement: Figure 5—source data 1. [file elife-82628-fig5-data1.zip › Figure 5-source data 1/figures with uncropped gels or blots.tif]

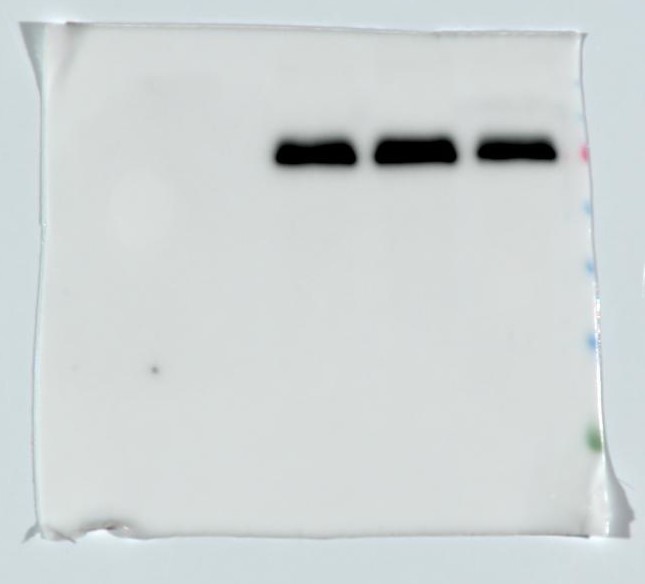

Supplement: Figure 5—source data 1. [file elife-82628-fig5-data1.zip › Figure 5-source data 1/raw unedited gels or blots/Figure 5-source data 1-1.tif]

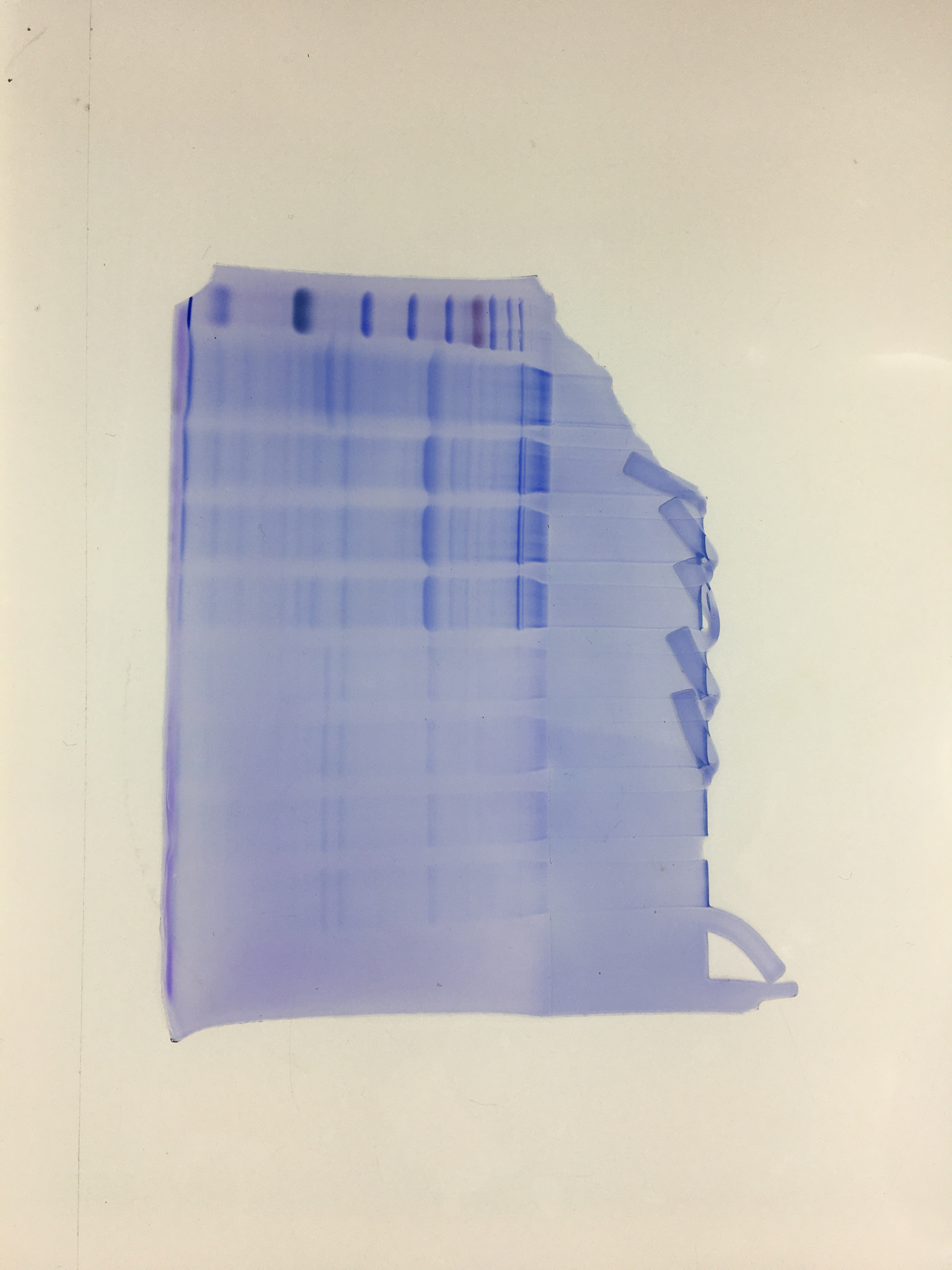

Supplement: Figure 5—source data 1. [file elife-82628-fig5-data1.zip › Figure 5-source data 1/raw unedited gels or blots/Figure 5-source data 1-10.tif]

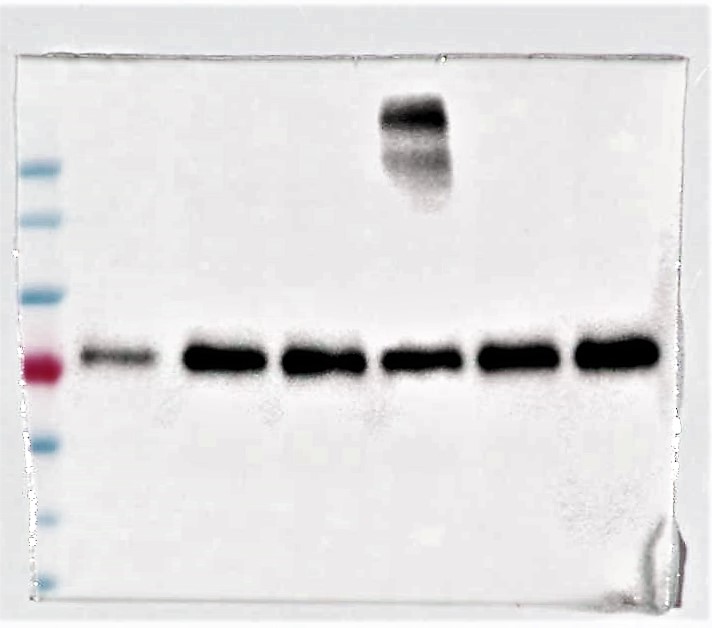

Supplement: Figure 5—source data 1. [file elife-82628-fig5-data1.zip › Figure 5-source data 1/raw unedited gels or blots/Figure 5-source data 1-11.tif]

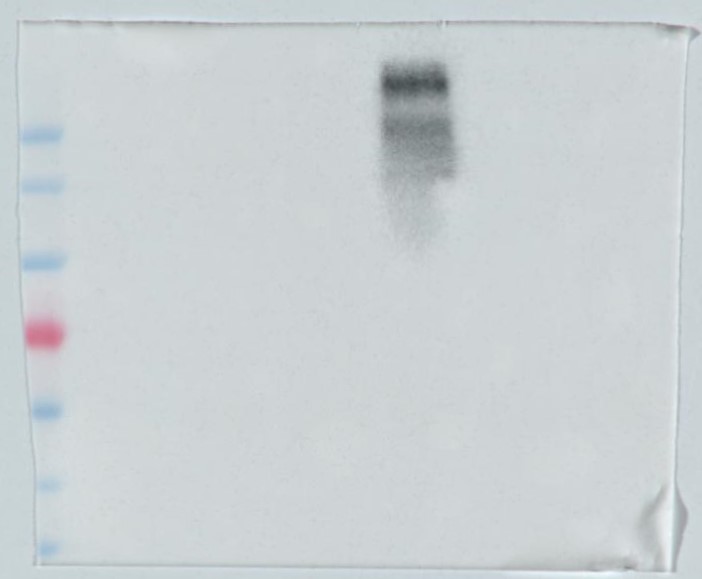

Supplement: Figure 5—source data 1. [file elife-82628-fig5-data1.zip › Figure 5-source data 1/raw unedited gels or blots/Figure 5-source data 1-12.tif]

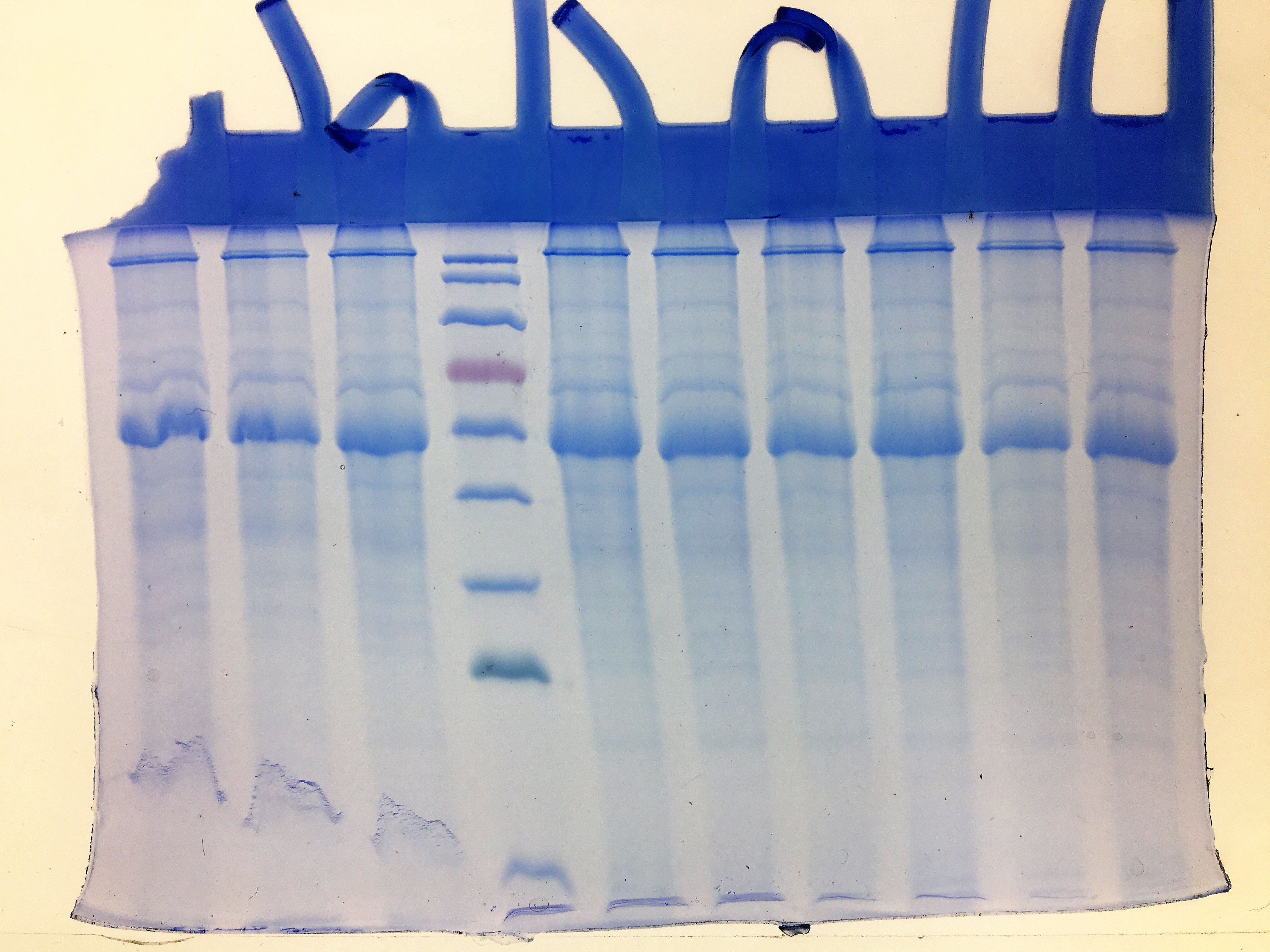

Supplement: Figure 5—source data 1. [file elife-82628-fig5-data1.zip › Figure 5-source data 1/raw unedited gels or blots/Figure 5-source data 1-13.tif]

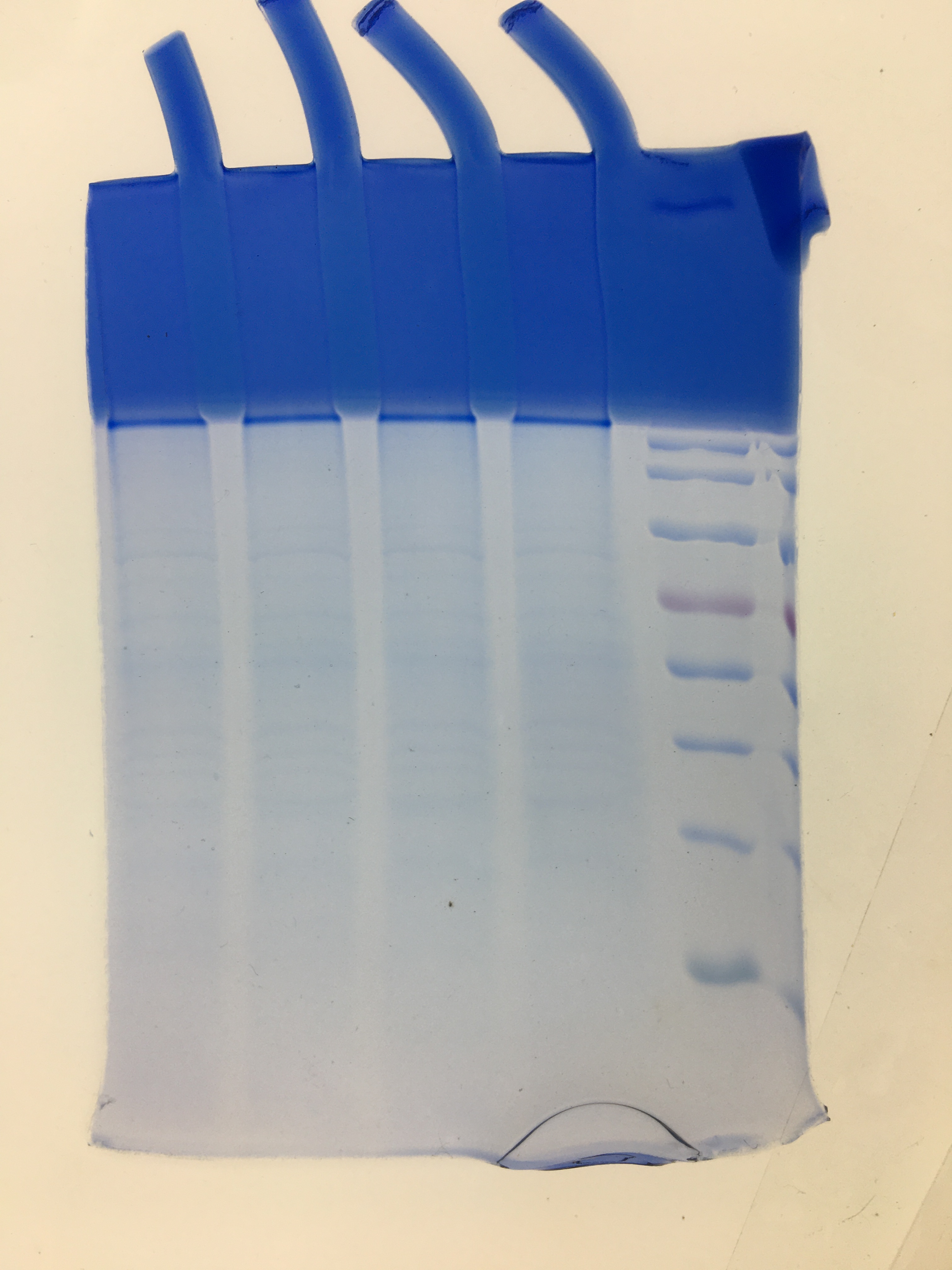

Supplement: Figure 5—source data 1. [file elife-82628-fig5-data1.zip › Figure 5-source data 1/raw unedited gels or blots/Figure 5-source data 1-2.tif]

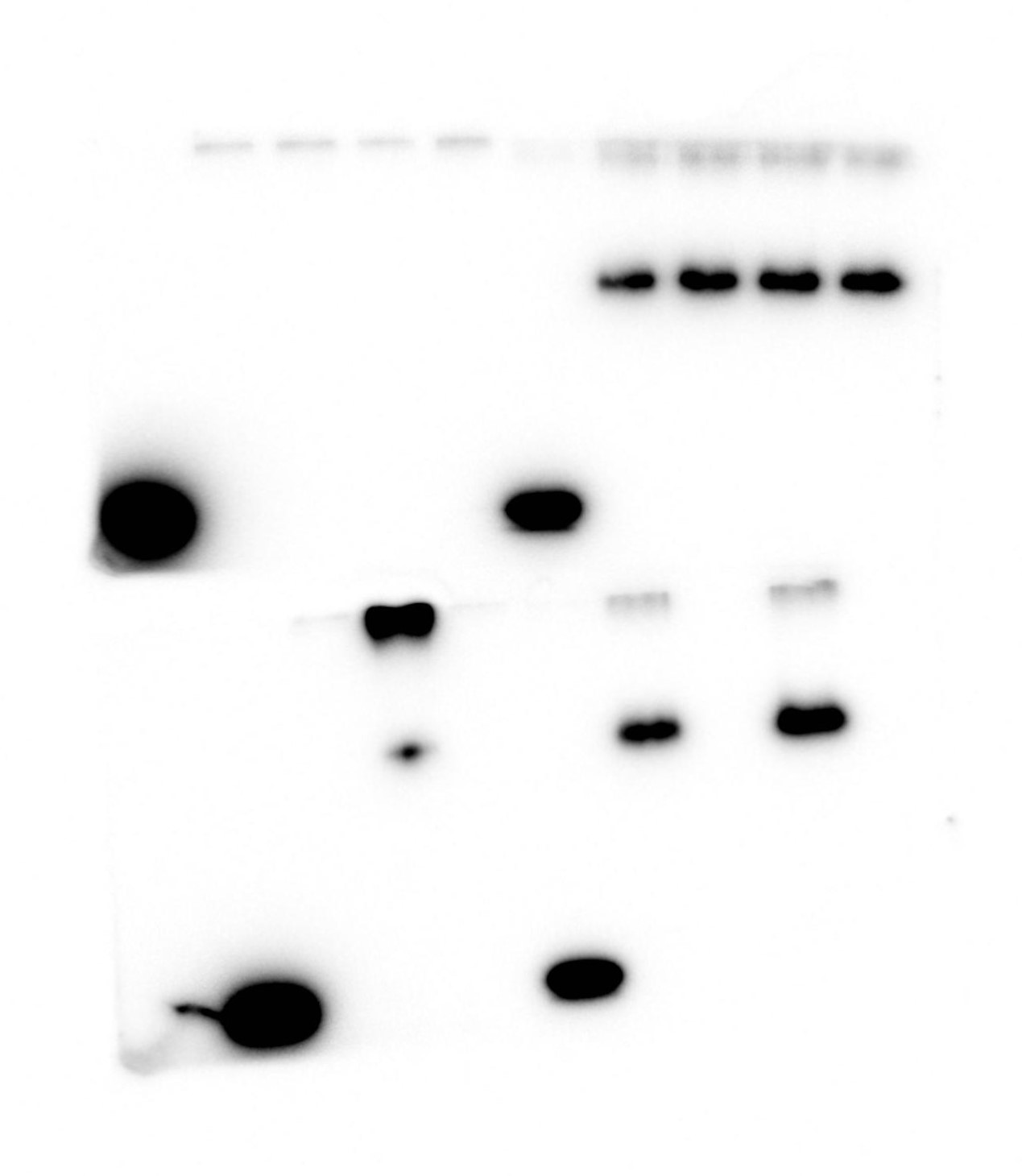

Supplement: Figure 5—source data 1. [file elife-82628-fig5-data1.zip › Figure 5-source data 1/raw unedited gels or blots/Figure 5-source data 1-3.tif]

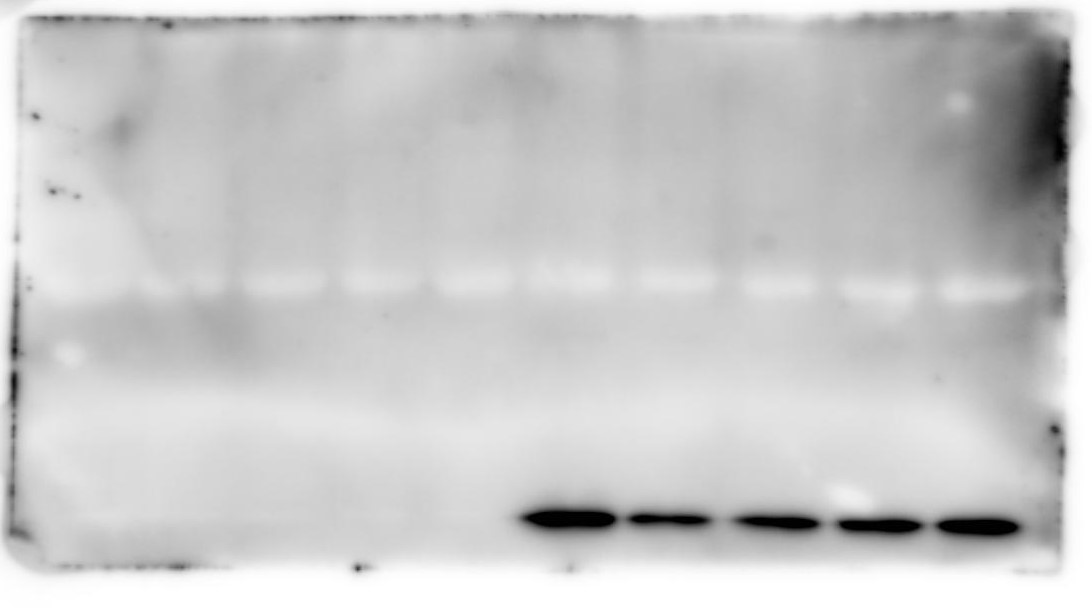

Supplement: Figure 5—source data 1. [file elife-82628-fig5-data1.zip › Figure 5-source data 1/raw unedited gels or blots/Figure 5-source data 1-4.tif]

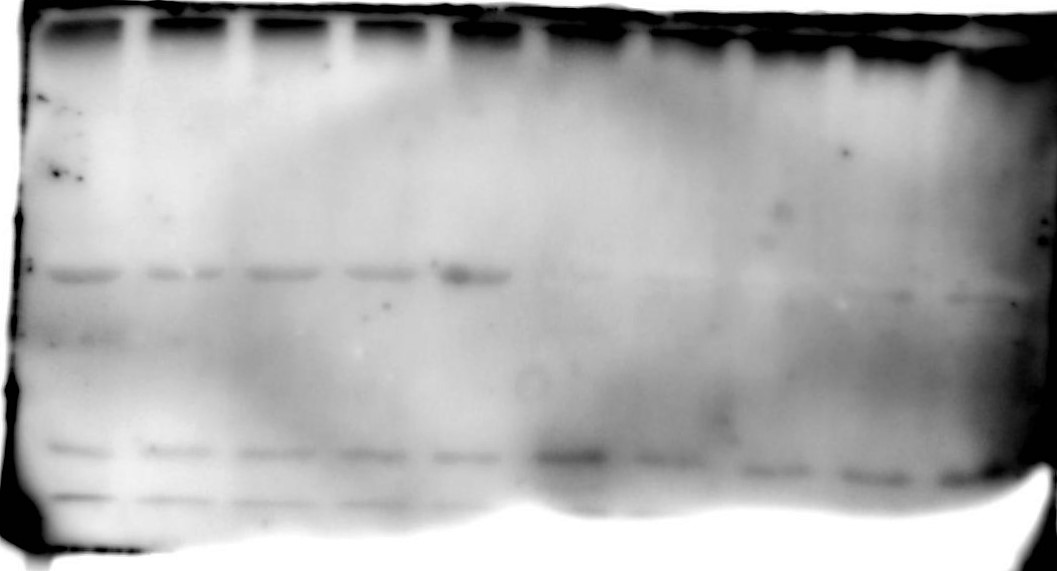

Supplement: Figure 5—source data 1. [file elife-82628-fig5-data1.zip › Figure 5-source data 1/raw unedited gels or blots/Figure 5-source data 1-5.tif]

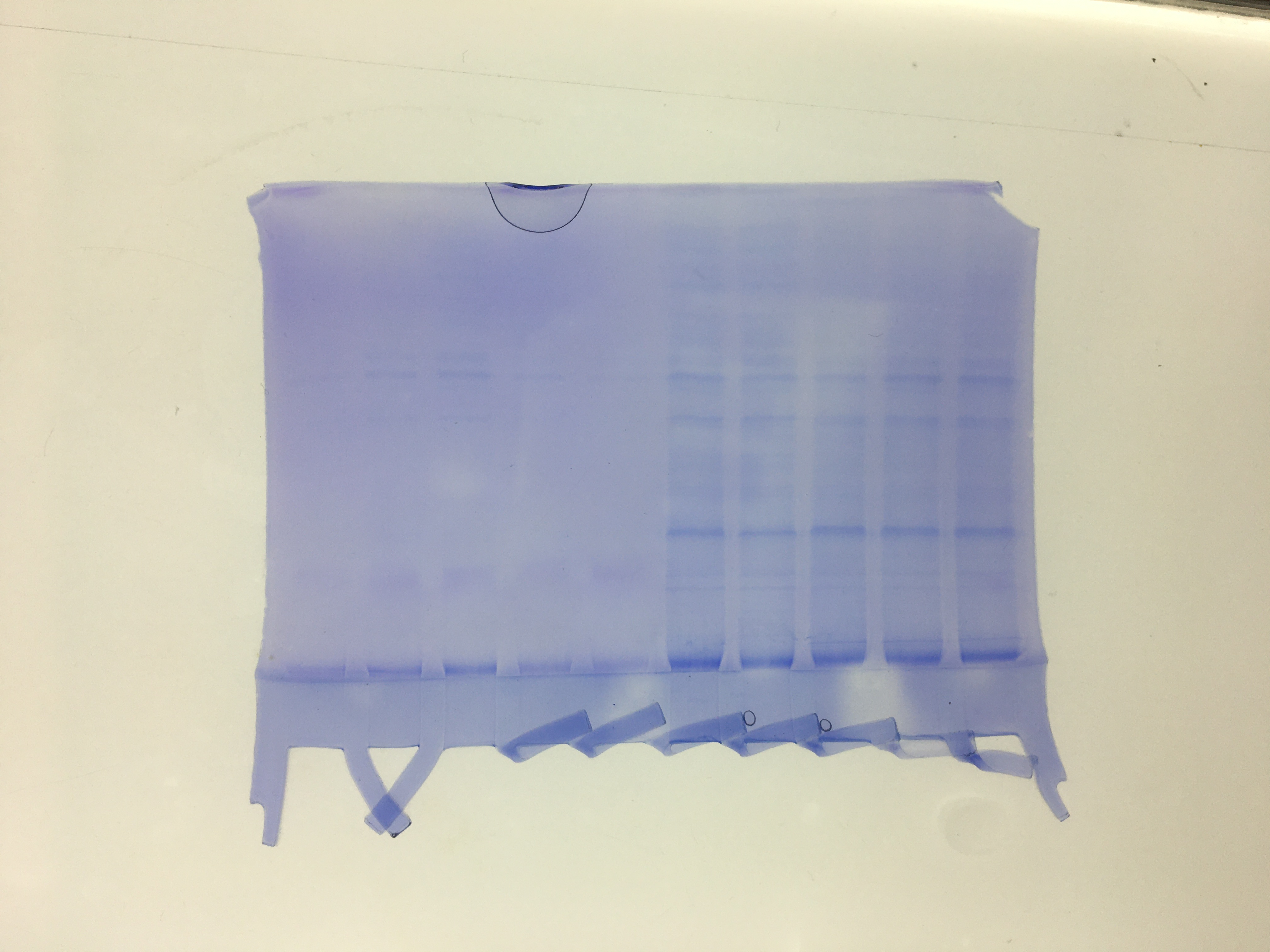

Supplement: Figure 5—source data 1. [file elife-82628-fig5-data1.zip › Figure 5-source data 1/raw unedited gels or blots/Figure 5-source data 1-6.tif]

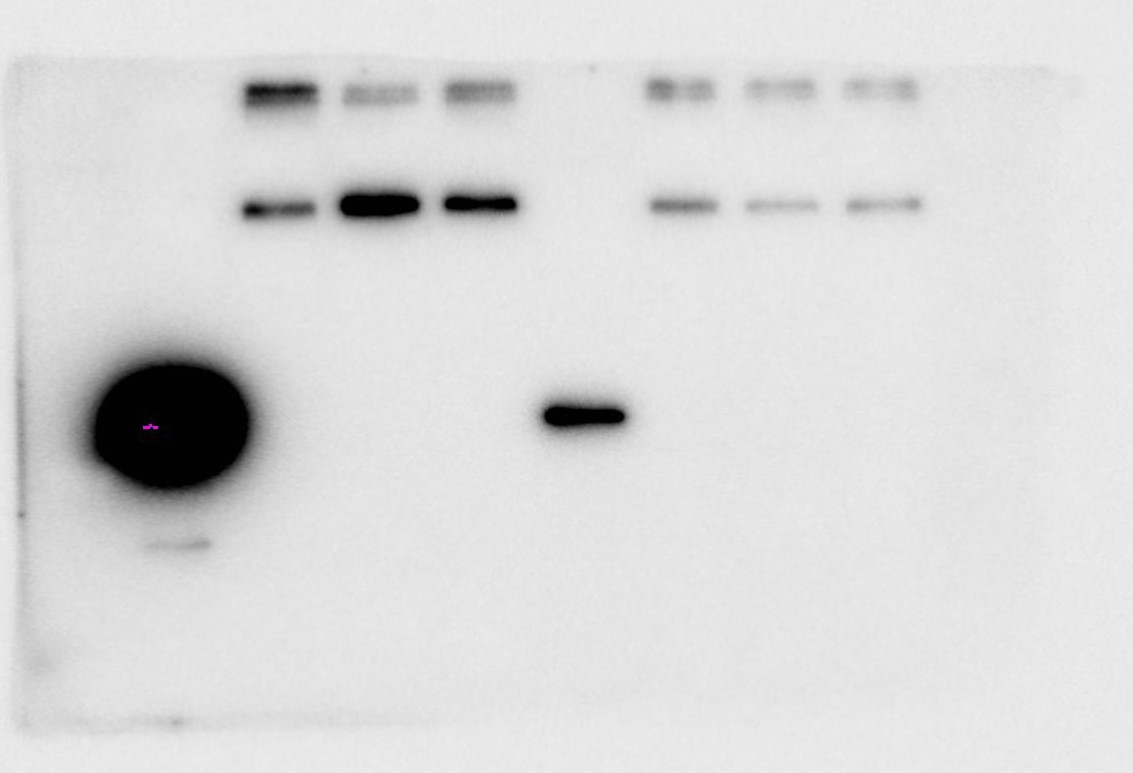

Supplement: Figure 5—source data 1. [file elife-82628-fig5-data1.zip › Figure 5-source data 1/raw unedited gels or blots/Figure 5-source data 1-7.tif]

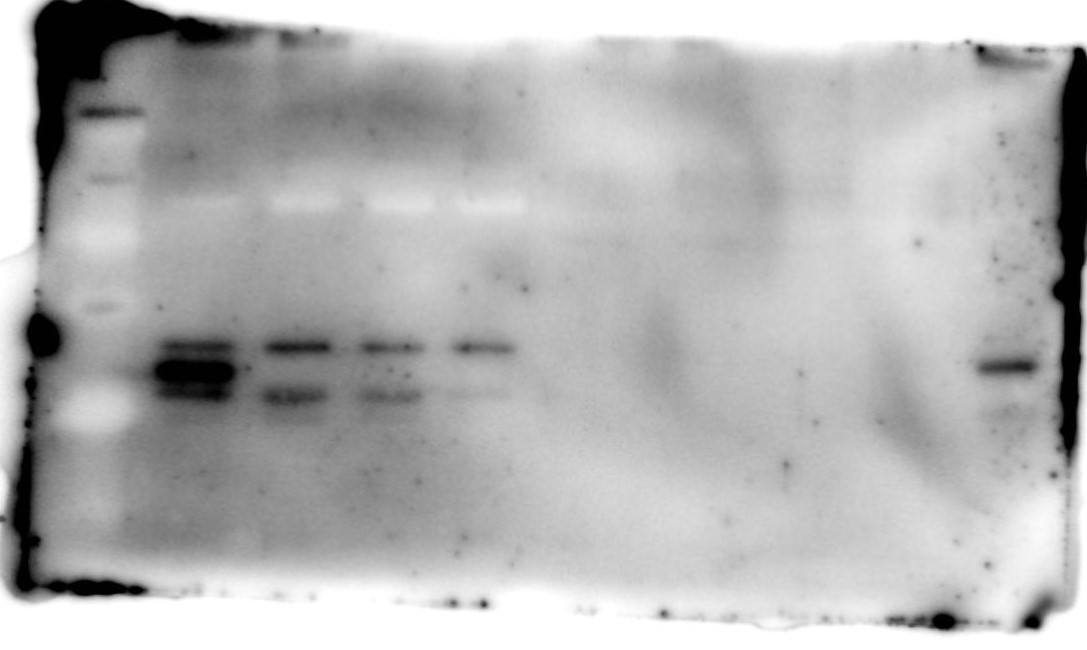

Supplement: Figure 5—source data 1. [file elife-82628-fig5-data1.zip › Figure 5-source data 1/raw unedited gels or blots/Figure 5-source data 1-8.tif]

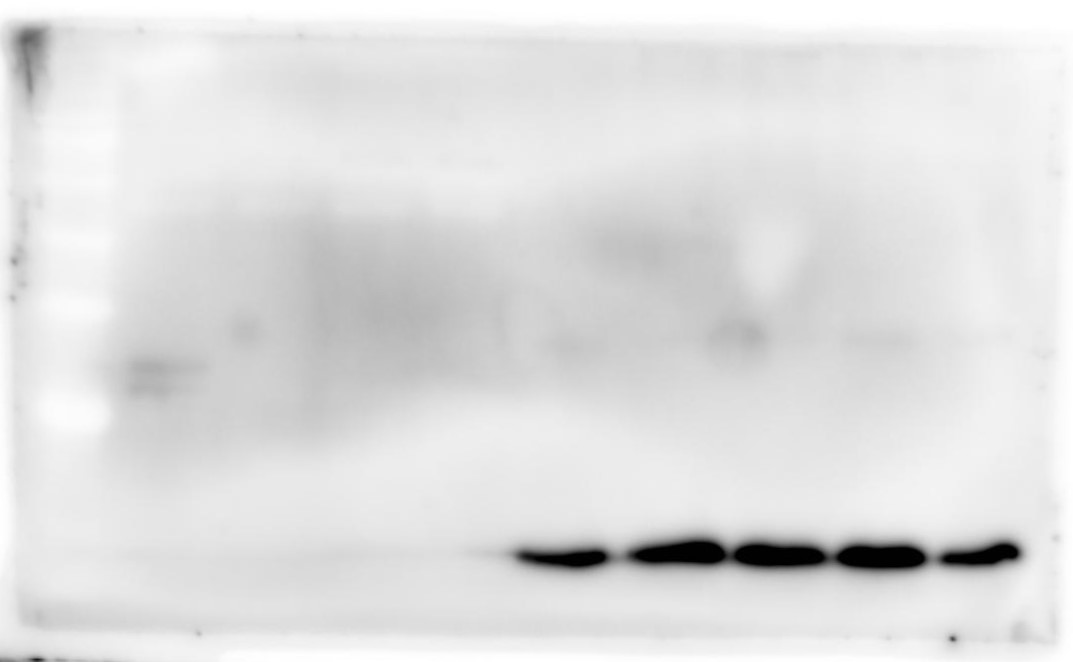

Supplement: Figure 5—source data 1. [file elife-82628-fig5-data1.zip › Figure 5-source data 1/raw unedited gels or blots/Figure 5-source data 1-9.tif]

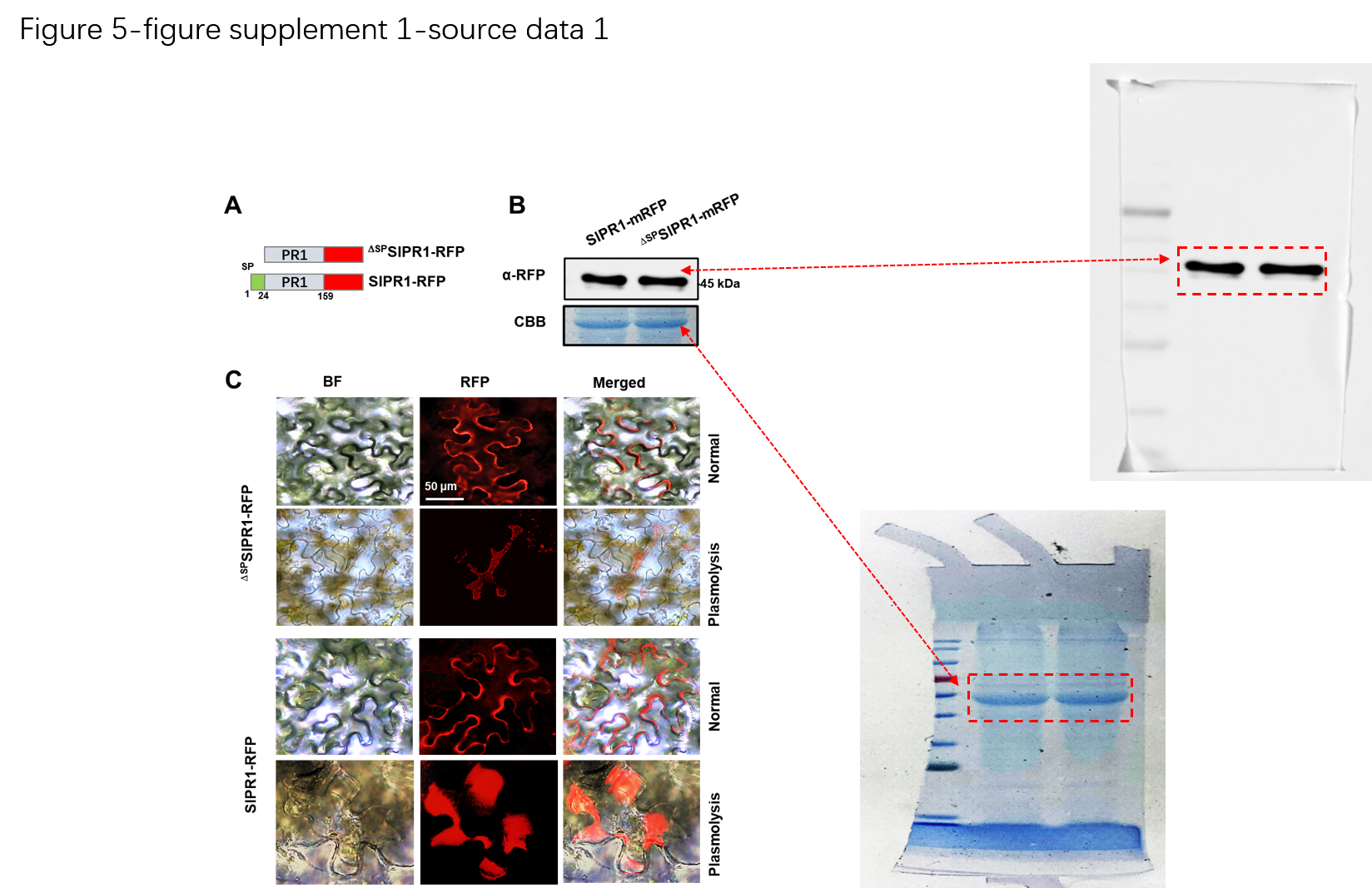

Supplement: Figure 5—figure supplement 1—source data 1. [file elife-82628-fig5-figsupp1-data1.zip › Figure 5-figure supplement 1-source data 1/figures with uncropped gels or blots.tif]

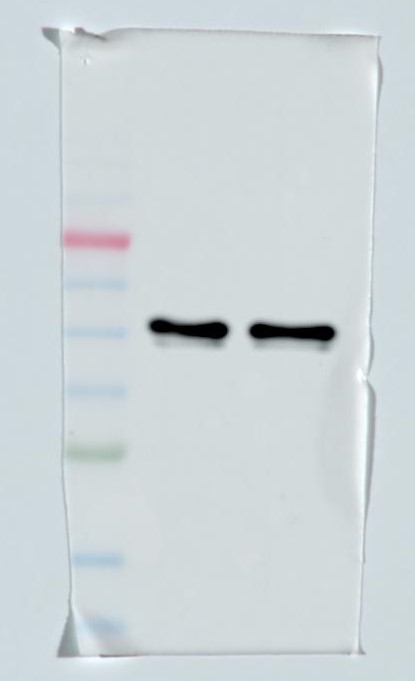

Supplement: Figure 5—figure supplement 1—source data 1. [file elife-82628-fig5-figsupp1-data1.zip › Figure 5-figure supplement 1-source data 1/raw unedited gels or blots/Figure 5-figure supplement 1-source data 1-1.tif]

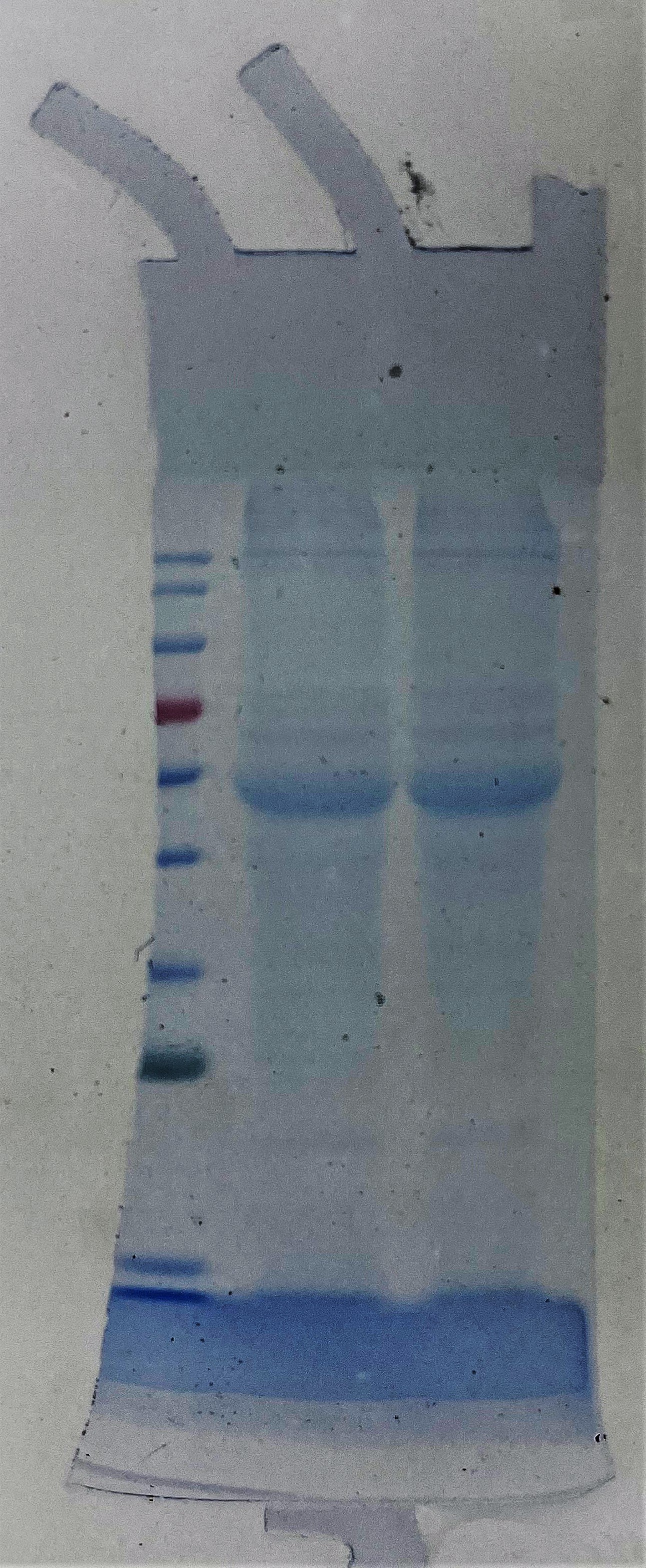

Supplement: Figure 5—figure supplement 1—source data 1. [file elife-82628-fig5-figsupp1-data1.zip › Figure 5-figure supplement 1-source data 1/raw unedited gels or blots/Figure 5-figure supplement 1-source data 1-2.tif]

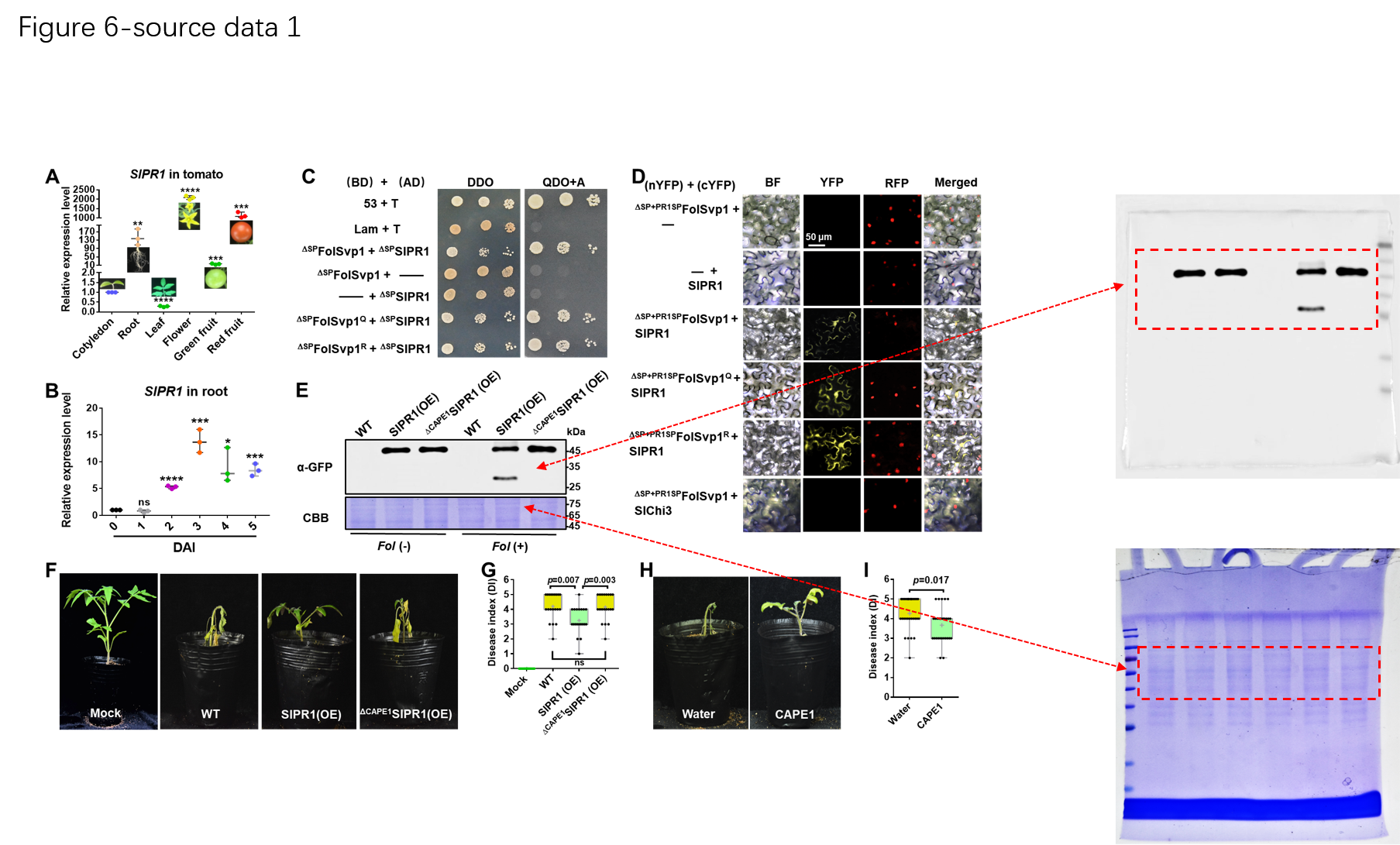

Supplement: Figure 6—source data 1. [file elife-82628-fig6-data1.zip › Figure 6-source data 1/figures with uncropped gels or blots.tif]

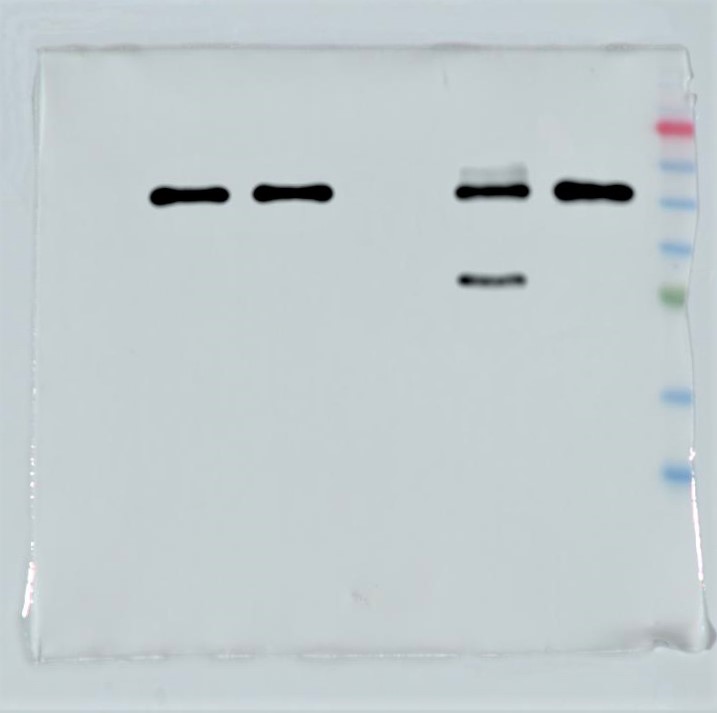

Supplement: Figure 6—source data 1. [file elife-82628-fig6-data1.zip › Figure 6-source data 1/raw unedited gels or blots/Figure 6-source data 1-1.tif]

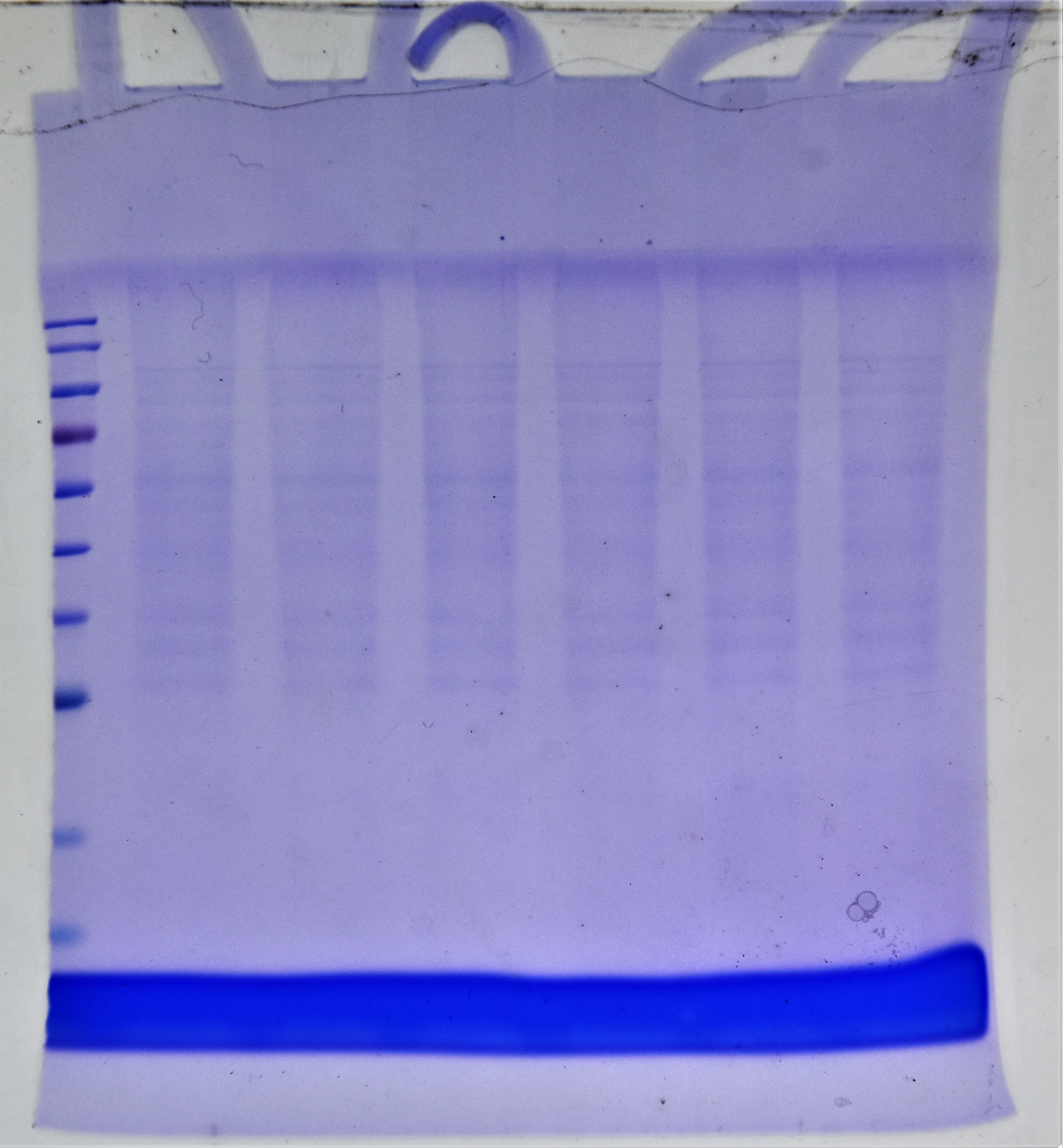

Supplement: Figure 6—source data 1. [file elife-82628-fig6-data1.zip › Figure 6-source data 1/raw unedited gels or blots/Figure 6-source data 1-2.tif]

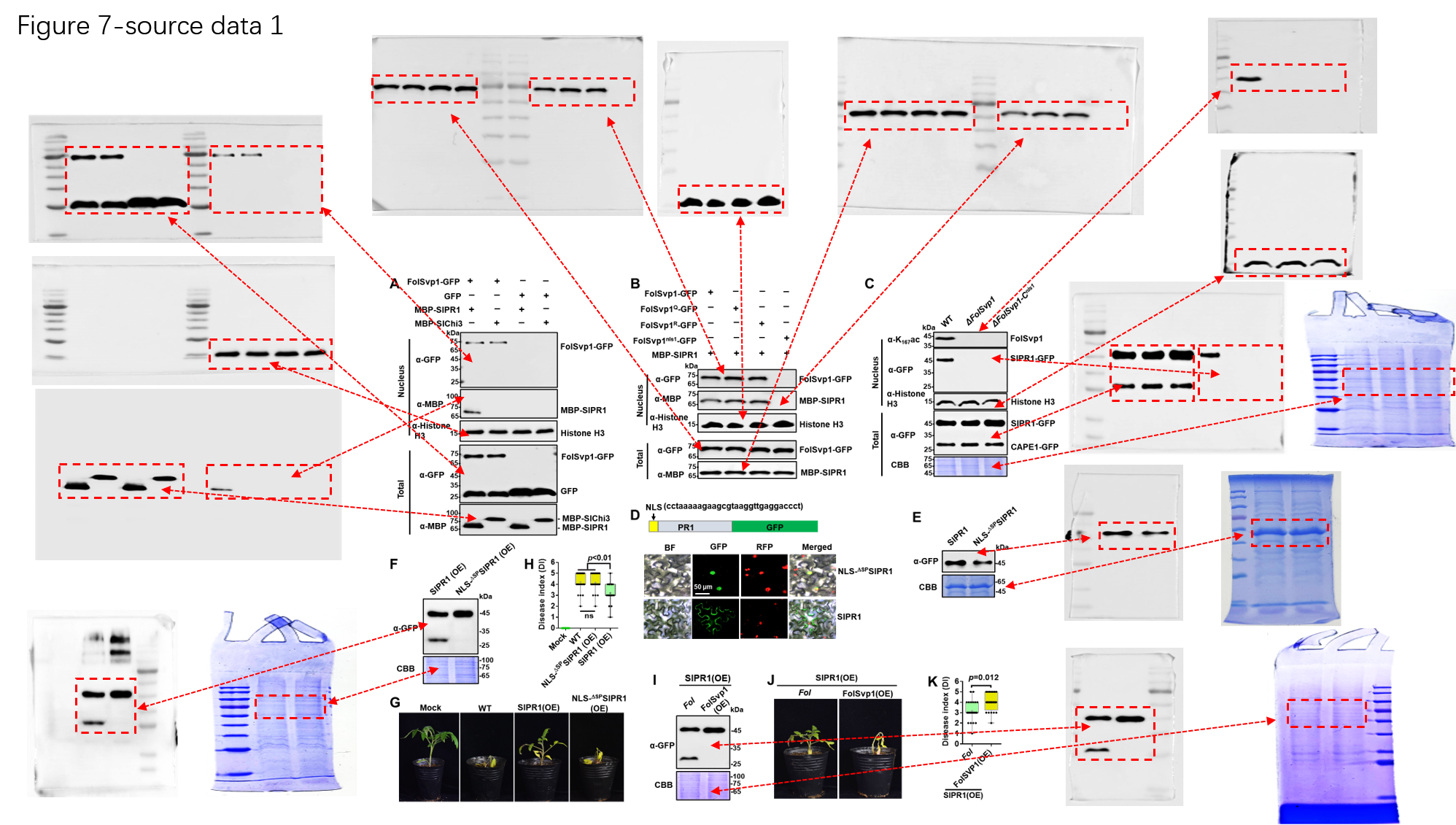

Supplement: Figure 7—source data 1. [file elife-82628-fig7-data1.zip › Figure 7-source data 1/figures with the uncropped gels or blots.tif]

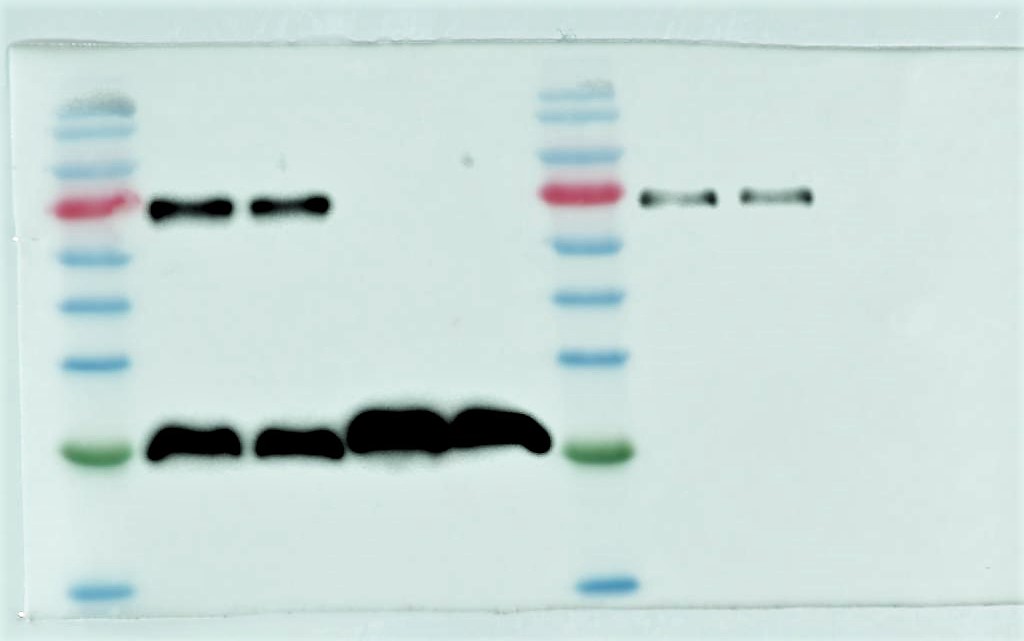

Supplement: Figure 7—source data 1. [file elife-82628-fig7-data1.zip › Figure 7-source data 1/raw unedited gels or blots/Figure 7-source data 1-1.tif]

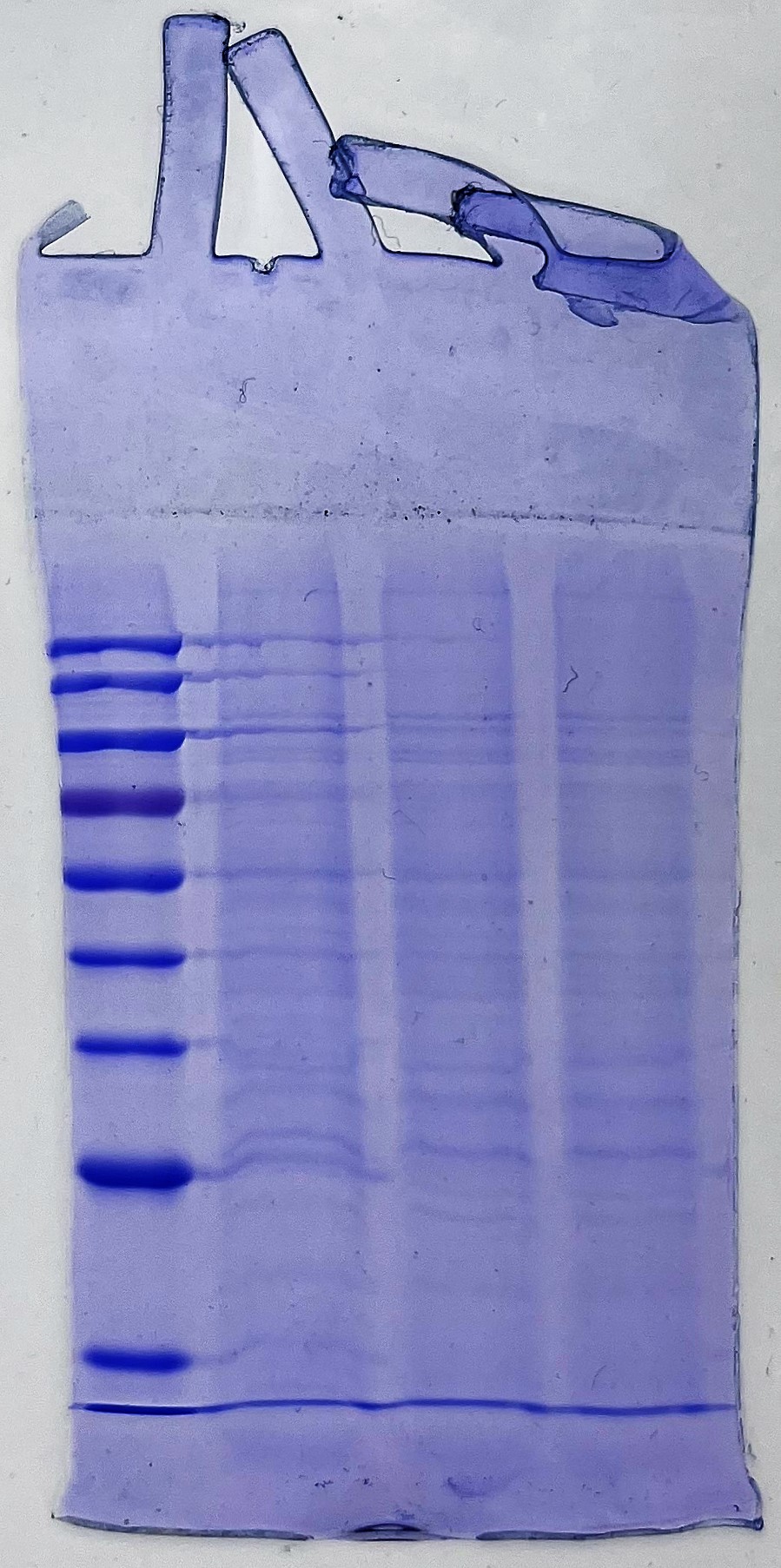

Supplement: Figure 7—source data 1. [file elife-82628-fig7-data1.zip › Figure 7-source data 1/raw unedited gels or blots/Figure 7-source data 1-10.tif]

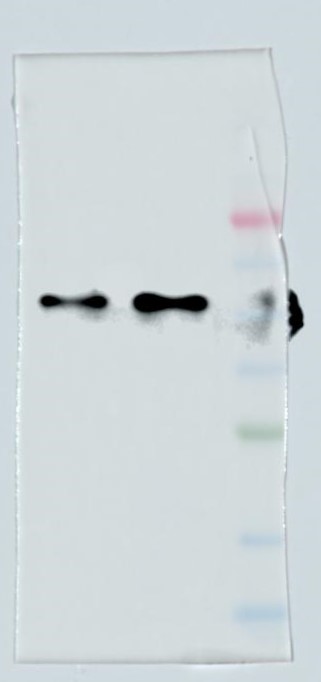

Supplement: Figure 7—source data 1. [file elife-82628-fig7-data1.zip › Figure 7-source data 1/raw unedited gels or blots/Figure 7-source data 1-11.tif]

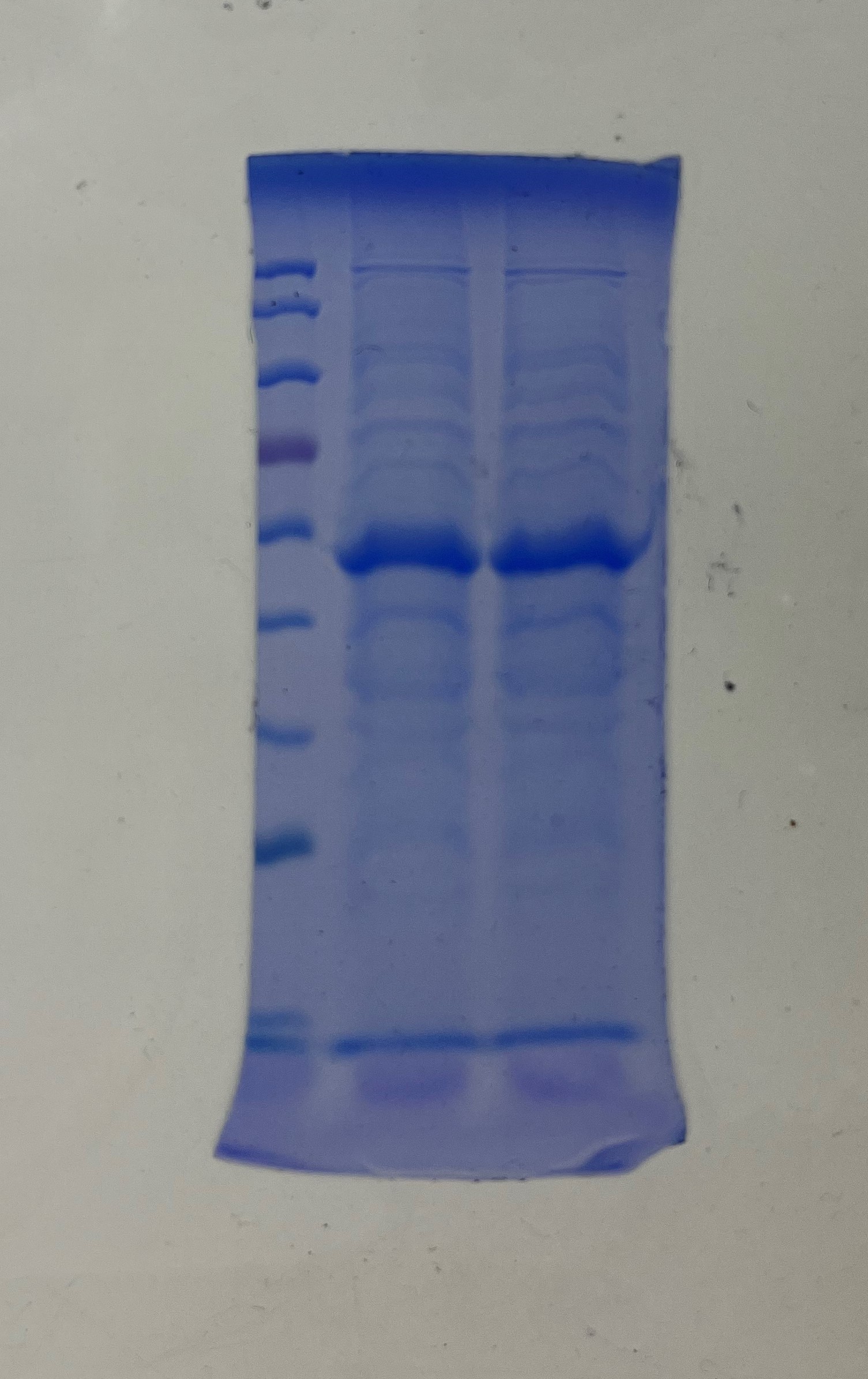

Supplement: Figure 7—source data 1. [file elife-82628-fig7-data1.zip › Figure 7-source data 1/raw unedited gels or blots/Figure 7-source data 1-12.tif]

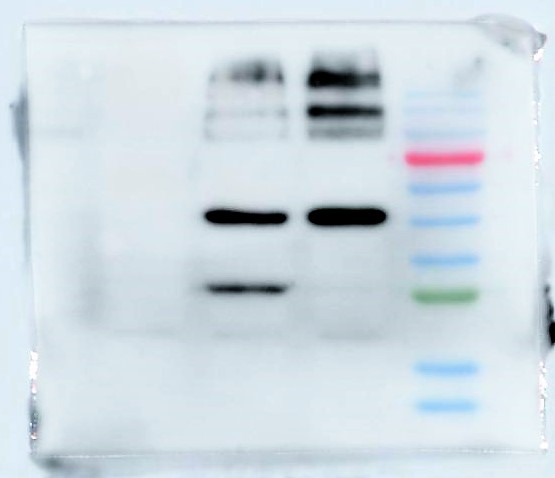

Supplement: Figure 7—source data 1. [file elife-82628-fig7-data1.zip › Figure 7-source data 1/raw unedited gels or blots/Figure 7-source data 1-13.tif]

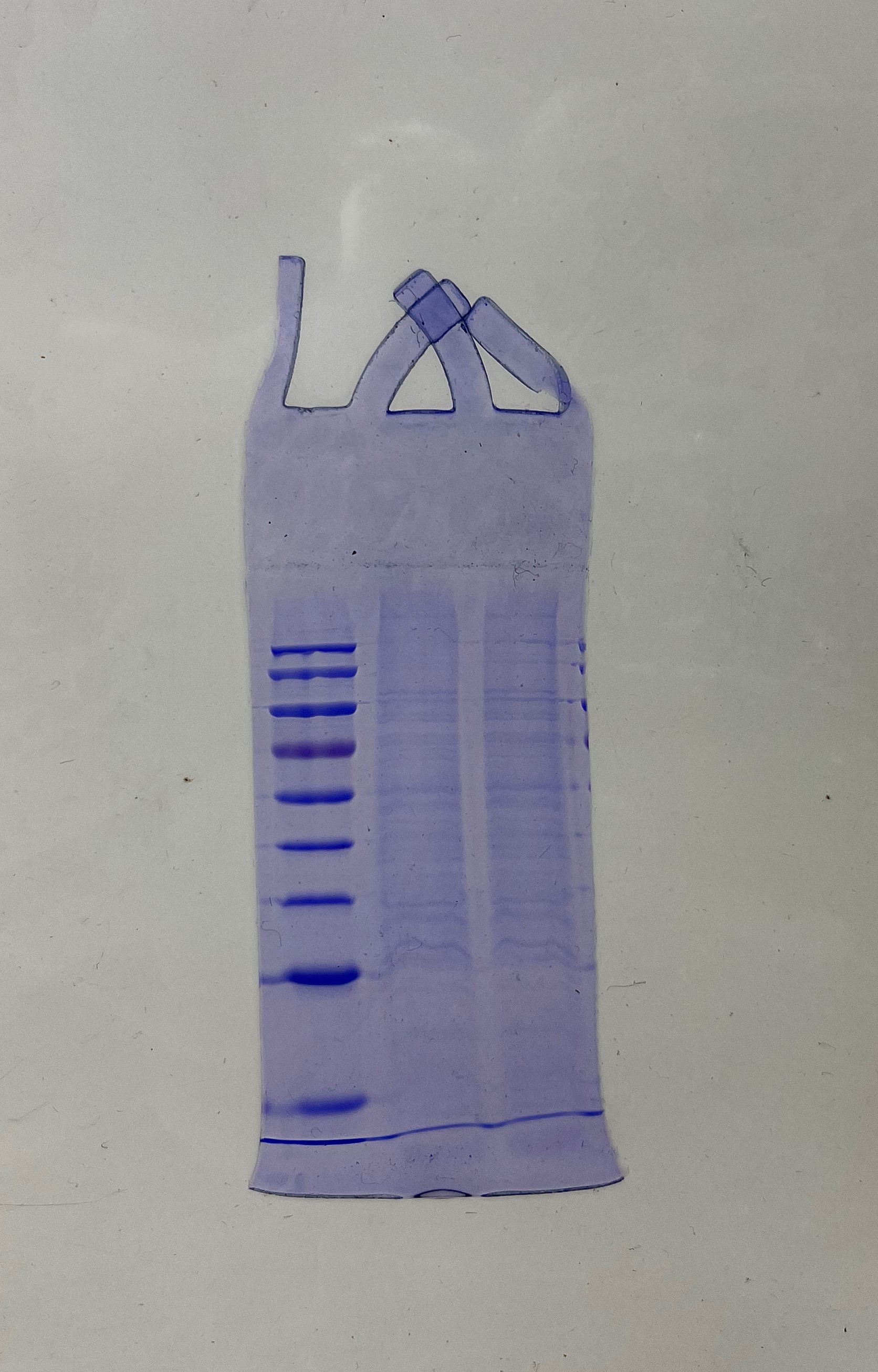

Supplement: Figure 7—source data 1. [file elife-82628-fig7-data1.zip › Figure 7-source data 1/raw unedited gels or blots/Figure 7-source data 1-14.tif]

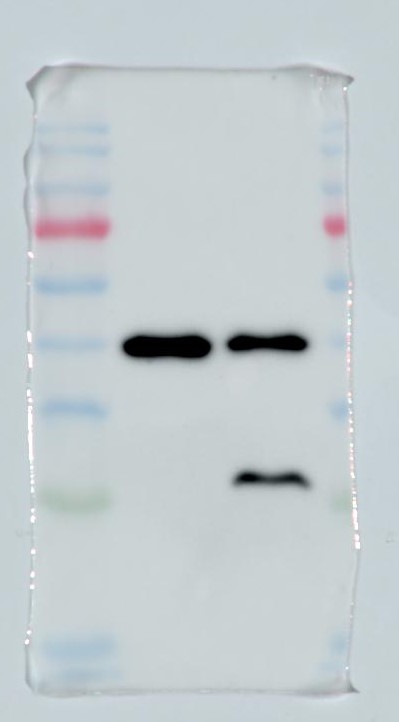

Supplement: Figure 7—source data 1. [file elife-82628-fig7-data1.zip › Figure 7-source data 1/raw unedited gels or blots/Figure 7-source data 1-15.tif]

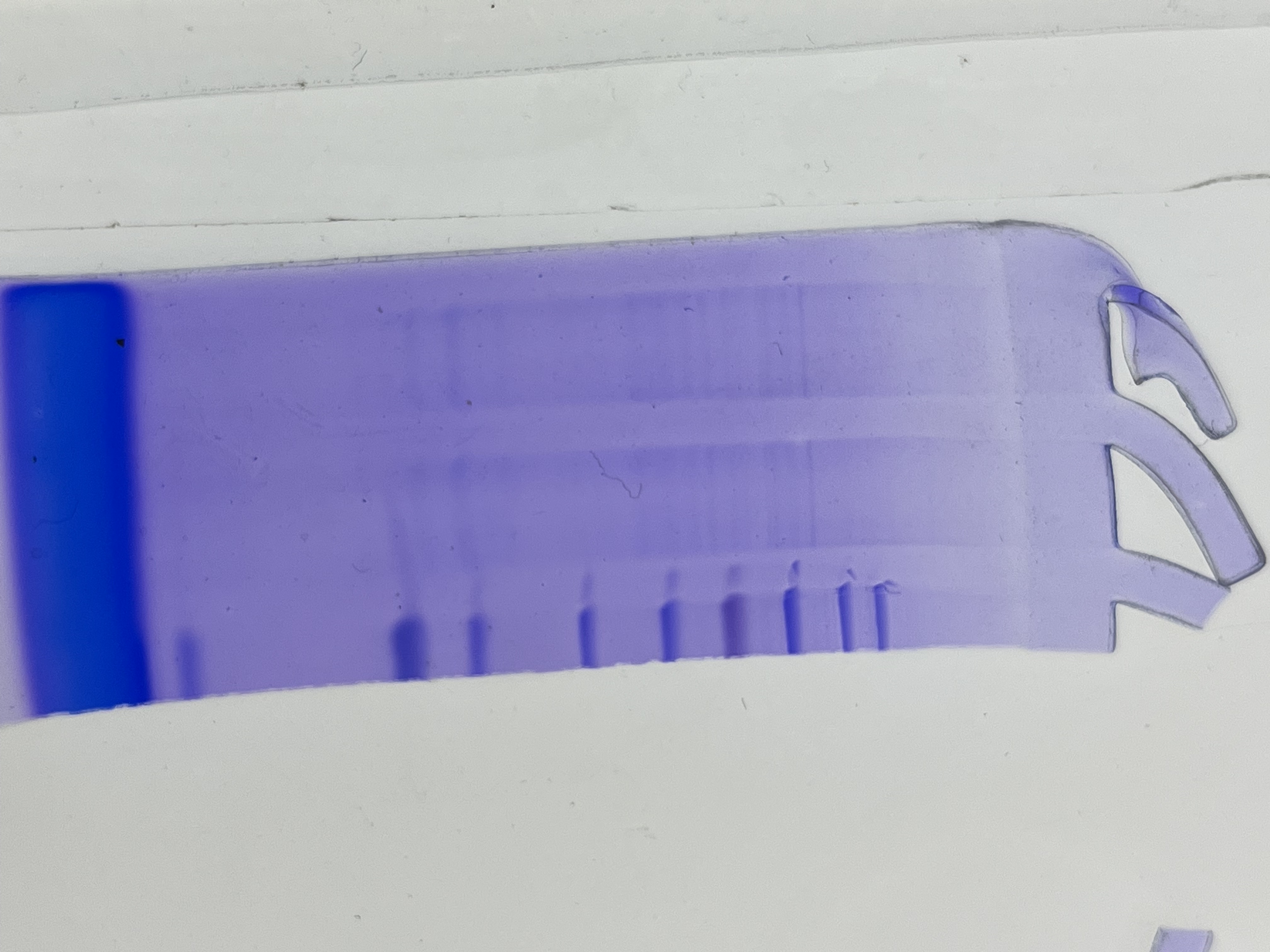

Supplement: Figure 7—source data 1. [file elife-82628-fig7-data1.zip › Figure 7-source data 1/raw unedited gels or blots/Figure 7-source data 1-16.tif]

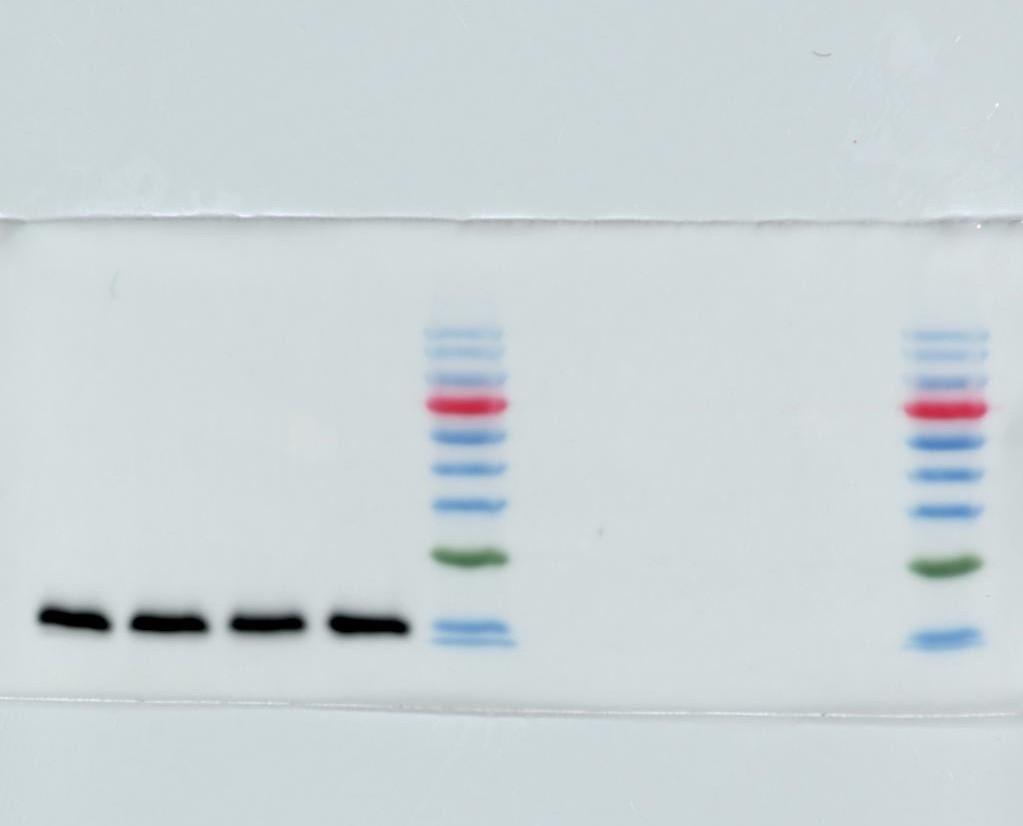

Supplement: Figure 7—source data 1. [file elife-82628-fig7-data1.zip › Figure 7-source data 1/raw unedited gels or blots/Figure 7-source data 1-2.tif]

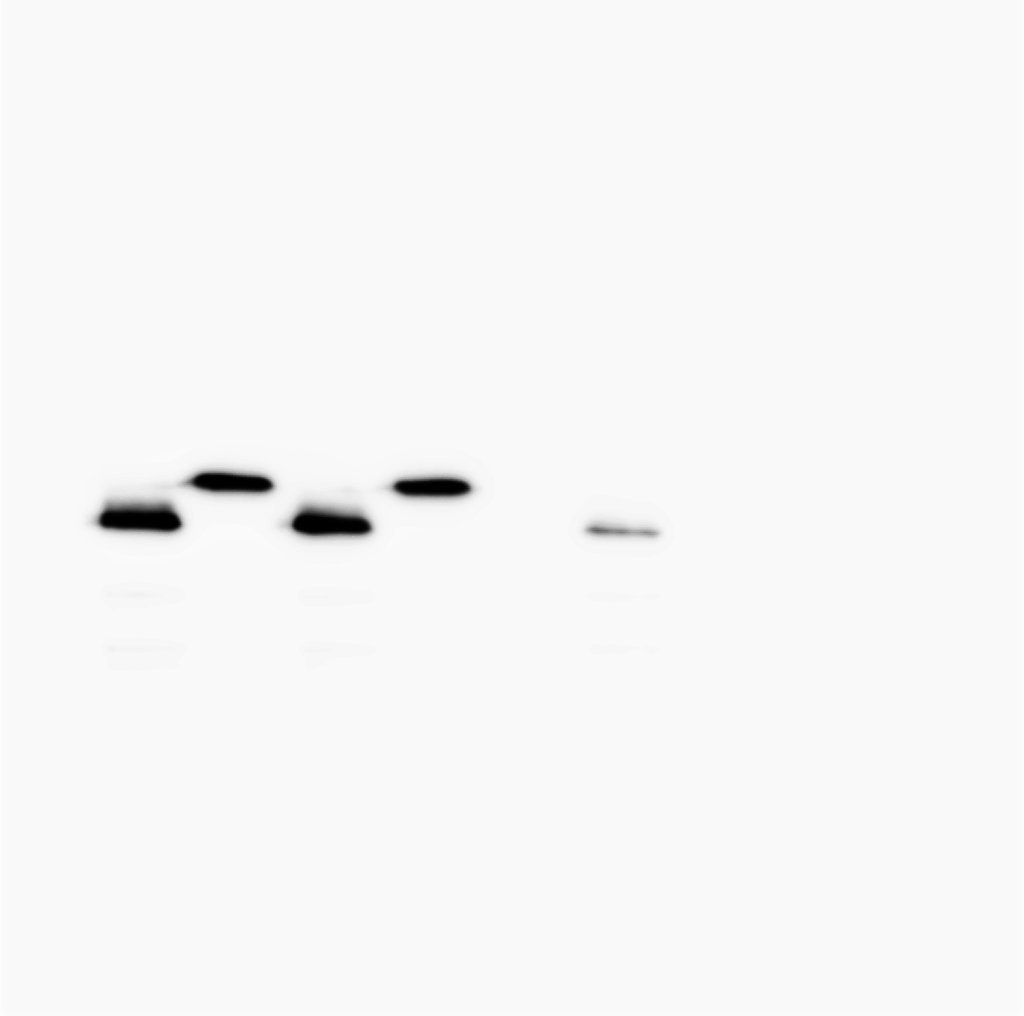

Supplement: Figure 7—source data 1. [file elife-82628-fig7-data1.zip › Figure 7-source data 1/raw unedited gels or blots/Figure 7-source data 1-3.tif]

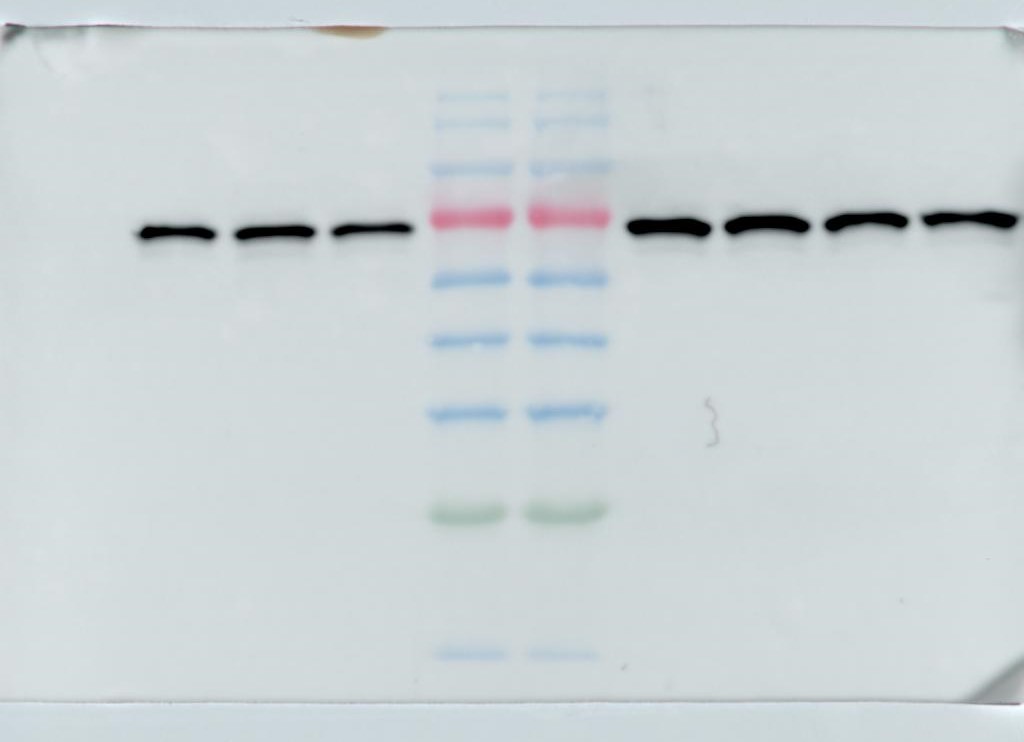

Supplement: Figure 7—source data 1. [file elife-82628-fig7-data1.zip › Figure 7-source data 1/raw unedited gels or blots/Figure 7-source data 1-4.tif]

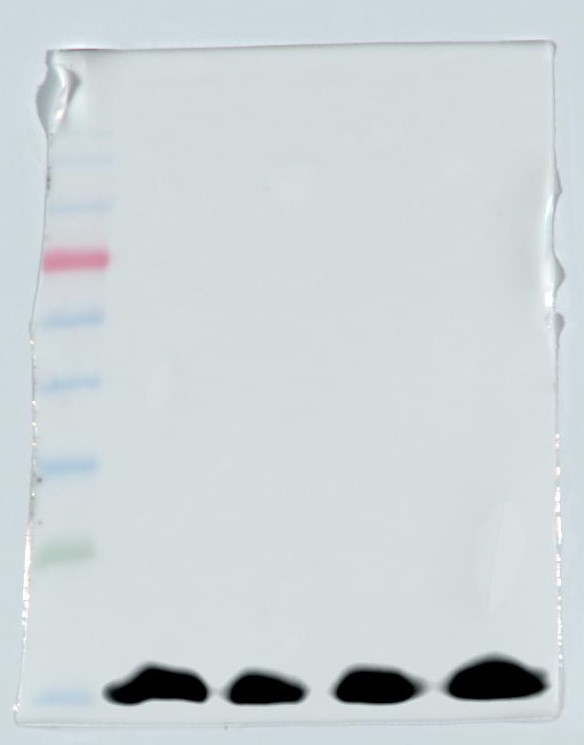

Supplement: Figure 7—source data 1. [file elife-82628-fig7-data1.zip › Figure 7-source data 1/raw unedited gels or blots/Figure 7-source data 1-5.tif]

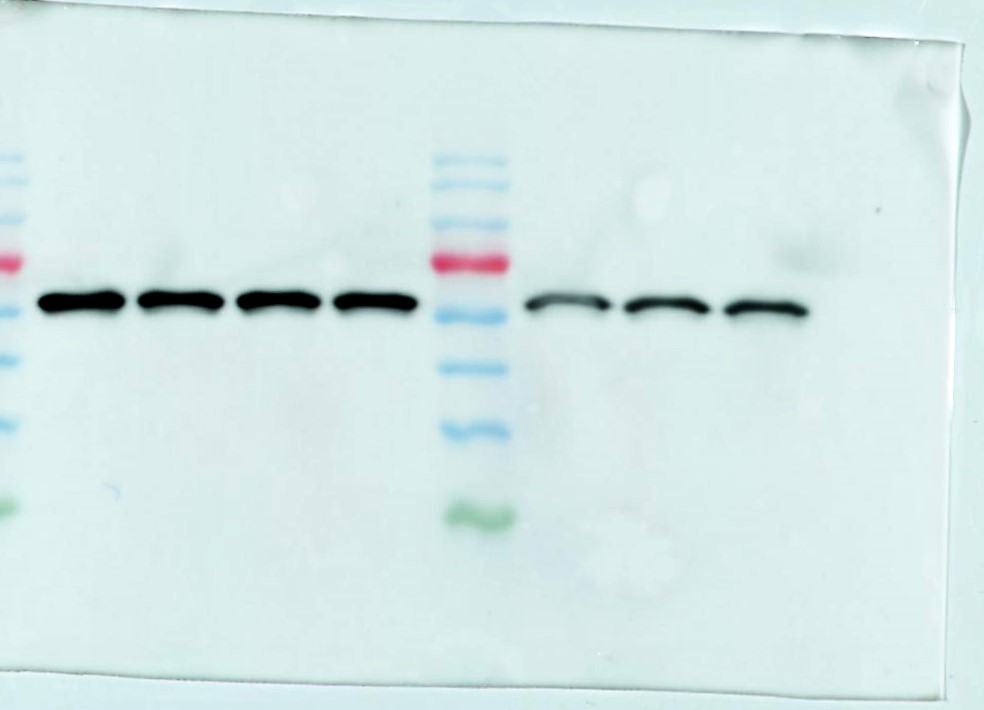

Supplement: Figure 7—source data 1. [file elife-82628-fig7-data1.zip › Figure 7-source data 1/raw unedited gels or blots/Figure 7-source data 1-6.tif]

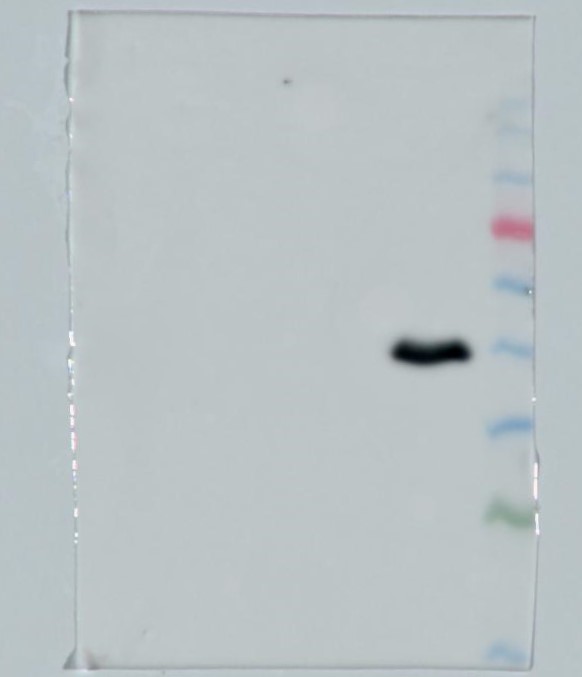

Supplement: Figure 7—source data 1. [file elife-82628-fig7-data1.zip › Figure 7-source data 1/raw unedited gels or blots/Figure 7-source data 1-7.tif]

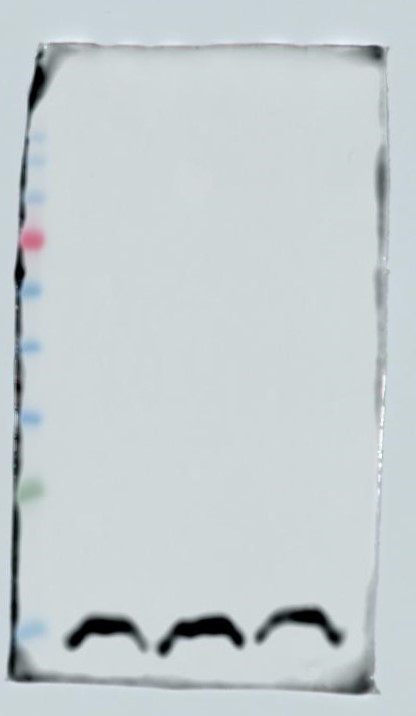

Supplement: Figure 7—source data 1. [file elife-82628-fig7-data1.zip › Figure 7-source data 1/raw unedited gels or blots/Figure 7-source data 1-8.tif]

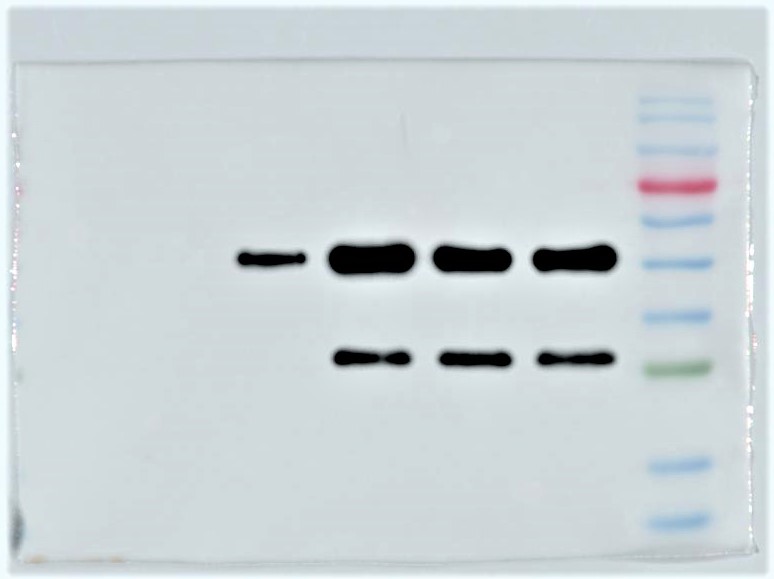

Supplement: Figure 7—source data 1. [file elife-82628-fig7-data1.zip › Figure 7-source data 1/raw unedited gels or blots/Figure 7-source data 1-9.tif]
