# Supplementary material for: Acetylation of a fungal effector that translocates host PR1 facilitates virulence
Source: eLife. 2022 Nov 14;11:e82628. doi: 10.7554/eLife.82628 (PMC9681213; doi:10.7554/eLife.82628)
Supplement: Figure 2—figure supplement 1—source data 2. [file elife-82628-fig2-figsupp1-data2.pdf]

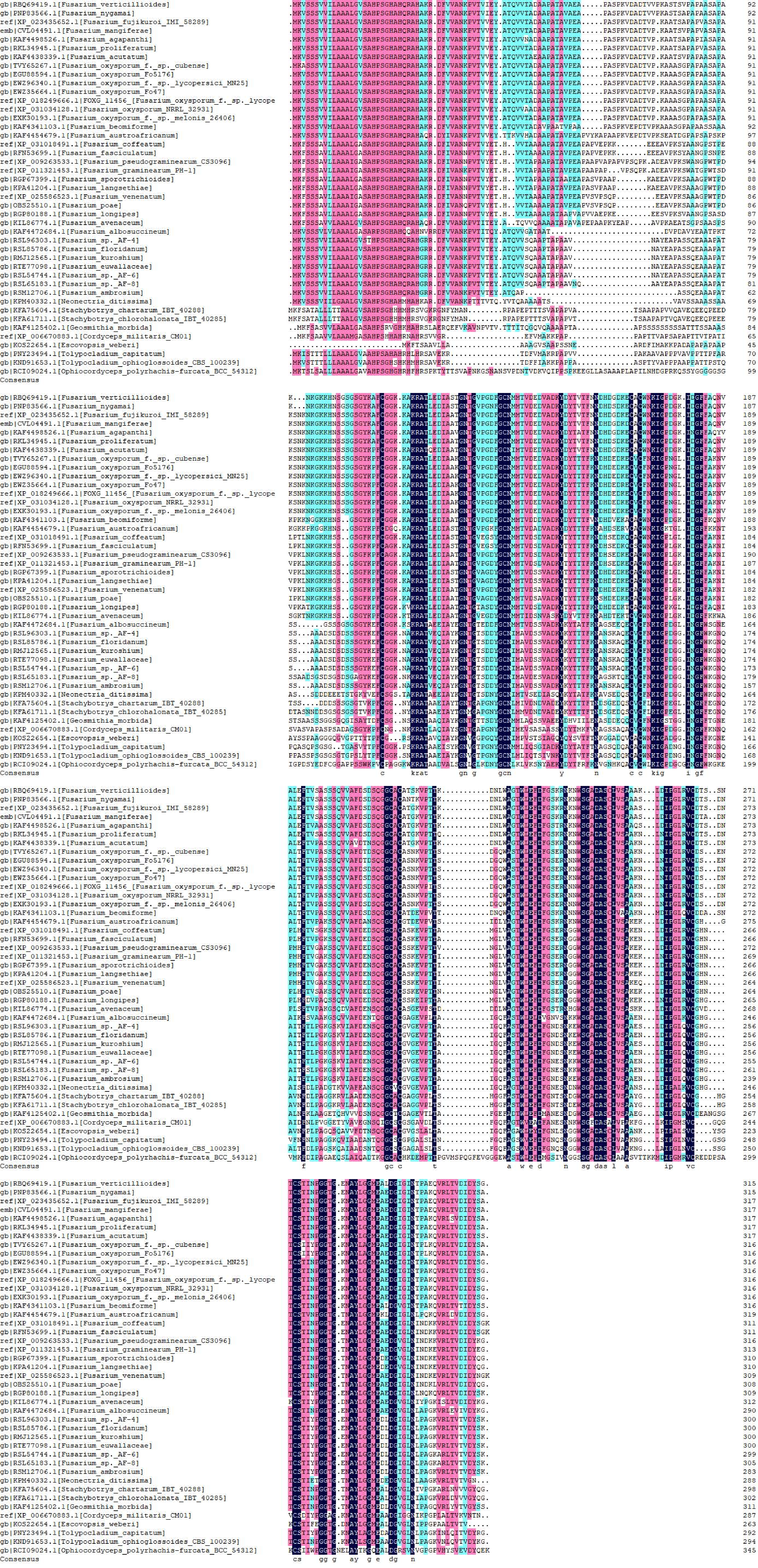
gb|RBQ69419.1[Fusarium_verticillioides]
gb|PNP83566.1[Fusarium_nygamai]
ref|XP_023435652.1[Fusarium_fujikuroi_IMI_58289]
emb|CVL04491.1[Fusarium_mangiferae]
gb|KAF4498526.1[Fusarium_agapanthi]
gb|RKL34945.1[Fusarium_proliferatum]
gb|KAF4438339.1[Fusarium_acutatum]
gb|TVY65267.1[Fusarium_oxysporum_f._sp._cubense]
gb|EGU88594.1[Fusarium_oxysporum_Fo5176]
gb|EWZ96340.1[Fusarium_oxysporum_f._sp._lycopersici_MN25]
gb|EWZ35664.1[Fusarium_oxysporum_Fo47]
ref|XP_018249666.1|FOXG_11456_[Fusarium_oxysporum_f._sp._lycope
ref|XP_031034128.1[Fusarium_oxysporum_NRRL_32931]
gb|EXK30193.1[Fusarium_oxysporum_f._sp._melonis_26406]
gb|KAF4341103.1[Fusarium_beomiforme]
gb|KAF4454679.1[Fusarium_austroafricanum]
ref|XP_031018491.1[Fusarium_coffeatum]
gb|RFN53699.1[Fusarium_fasciculatum]
ref|XP_009263533.1[Fusarium_pseudograminearum_CS3096]
ref|XP_011321453.1[Fusarium_graminearum_PH-1]
gb|RGP67399.1[Fusarium_sporotrichioides]
gb|KPA41204.1[Fusarium_langsethiae]
ref|XP_025586523.1[Fusarium_venenatum]
gb|OBS25510.1[Fusarium_poae]
gb|RGP80188.1[Fusarium_longipes]
gb|KIL86774.1[Fusarium_avenaceum]
gb|KAF4472684.1[Fusarium_albosuccineum]
gb|RSL96303.1[Fusarium_sp._AF-4]
gb|RSL85786.1[Fusarium_floridanum]
gb|RMJ12565.1[Fusarium_kuroshium]
gb|RTE77098.1[Fusarium_euwallaceae]
gb|RSL54744.1[Fusarium_sp._AF-6]
gb|RSL65183.1[Fusarium_sp._AF-8]
gb|RSM12706.1[Fusarium_ambrosium]
gb|KPM40332.1[Neonectria_ditissima]
gb|KFA75604.1[Stachybotrys_chartarum_IBT_40288]
gb|KFA61711.1[Stachybotrys_chlorohalonata_IBT_40285]
gb|KAF4125402.1[Geosmithia_morbida]
ref|XP_006670883.1[Cordyceps_militaris_CM01]
gb|KOS22654.1[Escovopsis_weberi]
gb|PNY23494.1[Tolypocladium_capitatum]
gb|KND91653.1[Tolypocladium_ophioglossoides_CBS_100239]
gb|RCI09024.1[Ophiocordyceps_polyrhachis-furcata_BCC_54312]
Consensus
