# Supplementary material for: Acetylation of a fungal effector that translocates host PR1 facilitates virulence
Source: eLife. 2022 Nov 14;11:e82628. doi: 10.7554/eLife.82628 (PMC9681213; doi:10.7554/eLife.82628)
Supplement: Figure 2—figure supplement 1—source data 3. [file elife-82628-fig2-figsupp1-data3.pdf]

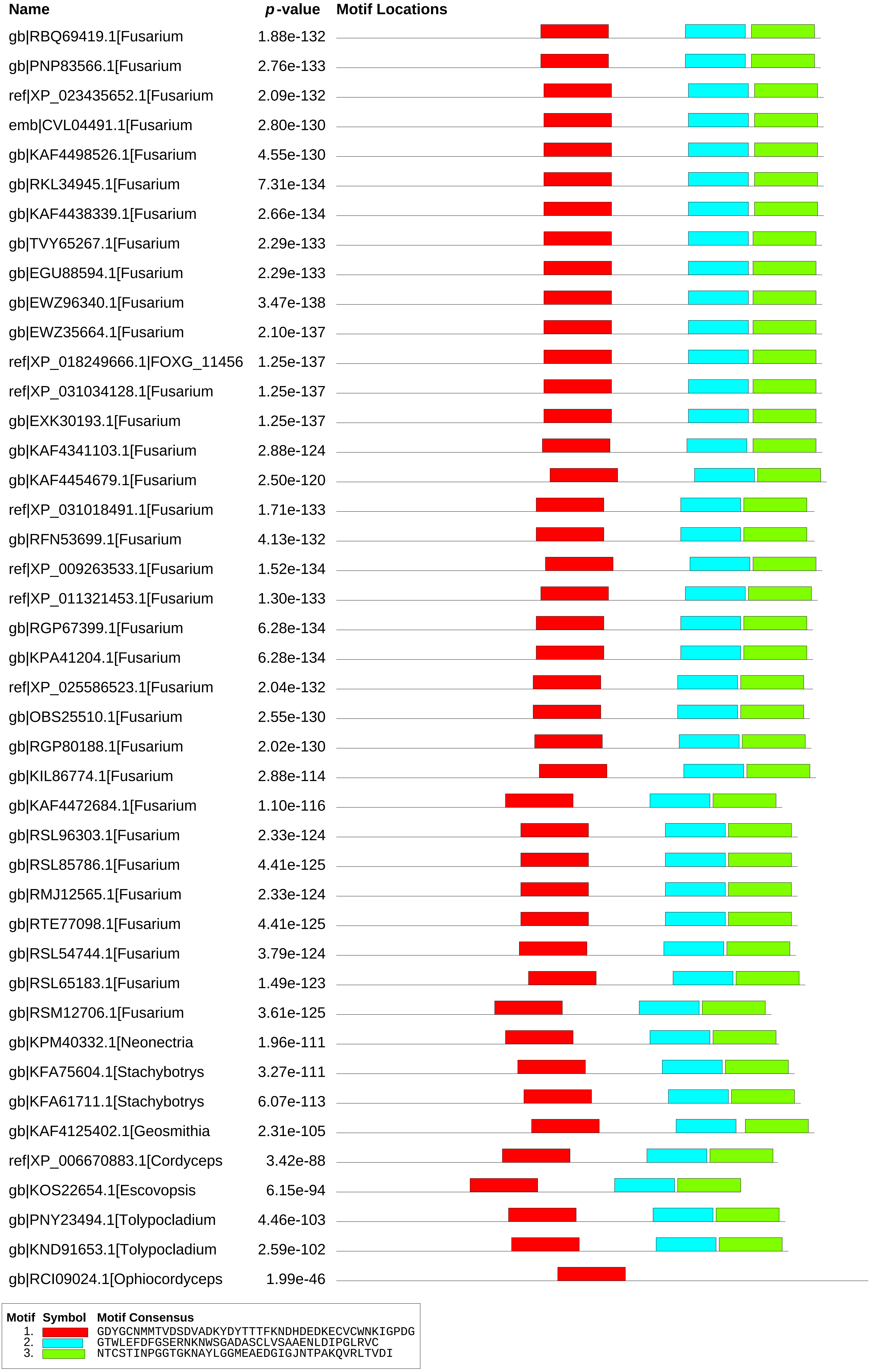
Name
p-value
Motif Locations
gb|RBQ69419.1[Fusarium 1.88e-132
gb|PNP83566.1[Fusarium 2.76e-133
ref|XP_023435652.1[Fusarium 2.09e-132
emb|CVL04491.1[Fusarium 2.80e-130
gb|KAF4498526.1[Fusarium 4.55e-130
gb|RKL34945.1[Fusarium 7.31e-134
gb|KAF4438339.1[Fusarium 2.66e-134
gb|TVY65267.1[Fusarium 2.29e-133
gb|EGU88594.1[Fusarium 2.29e-133
gb|EWZ96340.1[Fusarium 3.47e-138
gb|EWZ35664.1[Fusarium 2.10e-137
ref|XP_018249666.1|FOXG_11456 1.25e-137
ref|XP_031034128.1[Fusarium 1.25e-137
gb|EXK30193.1[Fusarium 1.25e-137
gb|KAF4341103.1[Fusarium 2.88e-124
gb|KAF4454679.1[Fusarium 2.50e-120
ref|XP_031018491.1[Fusarium 1.71e-133
gb|RFN53699.1[Fusarium 4.13e-132
ref|XP_009263533.1[Fusarium 1.52e-134
ref|XP_011321453.1[Fusarium 1.30e-133
gb|RGP67399.1[Fusarium 6.28e-134
gb|KPA41204.1[Fusarium 6.28e-134
ref|XP_025586523.1[Fusarium 2.04e-132
gb|OBS25510.1[Fusarium 2.55e-130
gb|RGP80188.1[Fusarium 2.02e-130
gb|KIL86774.1[Fusarium 2.88e-114
gb|KAF4472684.1[Fusarium 1.10e-116
gb|RSL96303.1[Fusarium 2.33e-124
gb|RSL85786.1[Fusarium 4.41e-125
gb|RMJ12565.1[Fusarium 2.33e-124
gb|RTE77098.1[Fusarium 4.41e-125
gb|RSL54744.1[Fusarium 3.79e-124
gb|RSL65183.1[Fusarium 1.49e-123
gb|RSM12706.1[Fusarium 3.61e-125
gb|KPM40332.1[Neonectria 1.96e-111
gb|KFA75604.1[Stachybotrys 3.27e-111
gb|KFA61711.1[Stachybotrys 6.07e-113
gb|KAF4125402.1[Geosmithia 2.31e-105
ref|XP_006670883.1[Cordyceps 3.42e-88
gb|KOS22654.1[Escovopsis 6.15e-94
gb|PNY23494.1[Tolypocladium 4.46e-103
gb|KND91653.1[Tolypocladium 2.59e-102
gb|RCI09024.1[Ophiocordyceps 1.99e-46
Motif Symbol Motif Consensus
1. GDYGCNMMTVDSDVADKYDYTTTFKNDHDEDKECVCWNKIGPDG
2. GTWLEFDFGSERNKNWSGADASCLVSAAENLDIPGLRVC
3. NTCSTINPGGTGKNAYLGGMEAEDGIGJNTPAKQVRLTVDI
